# Comparing large language models and search engine responses to common orthodontic questions

**DOI:** 10.1371/journal.pone.0339908

**Published:** 2026-01-02

**Authors:** Yuanyuan Ren, Jing Sun

**Affiliations:** 1 School of Nursing, Peking University, Beijing, China; 2 Department of Community Nursing, School of Nursing, Peking University, Beijing, China; Woldia University, ETHIOPIA

## Abstract

**Background:**

Large Language Models (LLMs) highlight their potential in supporting patient education and self-management. Their performance in responses to orthodontic questions has yet to be explored.

**Objectives:**

This study aims to compare the quality, empathy, readability, and satisfaction of responses from LLMs and search engines on common orthodontic questions.

**Methods:**

Forty-five common orthodontic questions (six categories) and a prompt were developed, and a self-designed multidimensional evaluation questionnaire was constructed. Questions were presented to 5 LLMs and 3 search engines on December,22,2024. The primary outcomes were the median expert-rated scores of LLMs versus search engine responses on quality, empathy, readability, and satisfaction, using 5- or 10-point Likert scales.

**Results:**

LLMs scored significantly higher than search engines in quality (4.00 vs. 3.50, p < 0.001), empathy (3.75 vs. 3.50, p < 0.001), readability (4.00 vs. 3.75, p < 0.001), and satisfaction (8.00 vs. 7.25, p < 0.001). LLM-generated responses were rated significantly higher than those from search engines in therapeutic outcomes category, appliance selection category and cost category.

**Conclusions:**

In this cross-sectional study, the LLMs, particularly GPT-4o, outperformed search engines. These results indicate the potential of LLMs as supplementary tools for orthodontic patient education and self-management.

## Introduction

Patient education and self-management improve outcomes such as medication adherence (d = 0.316), and reduce 30-day readmissions by 21% and hospitalizations by 60% [1–3]. However, limited consultation time and heavy workloads hinder communication, increasing the risk of adverse outcomes (HR = 1.27–1.75) [4–6].

The Internet has become a primary source for individuals looking for health information [7]. Search engines deliver health information by retrieving and ranking existing web content through keyword-based algorithms [8], which were used by approximately 83% of health-information seekers from 2016 to 2021, and 75% report that the results influence their self-management decisions [9]. Artificial intelligence (AI), which seeks to create machines that perform tasks requiring human-like cognition [10], made a breakthrough with deep learning advances from 2012 [11]. A national survey [12] published as a peer-reviewed Data Brief in *JAMA* reported that 63% of respondents used AI tools for information. Large Language Models (LLMs), a class of artificial intelligence (AI) systems, leverage deep neural networks to generate natural language text by processing vast datasets [13]. LLMs excel in executing complex tasks such as natural language processing and human-computer interaction, demonstrating advanced capabilities in contextual understanding and linguistic pattern recognition [14], having rapidly expanded applications in healthcare [15,16], transforming areas such as documentation, clinical diagnosis, and patient education [17].

Wang et al.[18] conducted a bibliometric analysis of 5,284 articles of generative AI in medicine and identified that “medical education” was the burst keyword (intensity: 4.58) during 2023−2024. Ma et al. [19] used GPT-4 to role-play patients and deliver personalized ICU discharge education. Hao et al. [20] developed MedEduChat (an LLM-based chatbot for prostate cancer patient education) which enables personalized and semi-structured health education interactions. The EHRTutor framework, developed by Zhang et al, enables personalized education for patient discharge instructions [21]. While Serhat Aydin et al.[22] comprehensively reviewed advancements in LLMs for patient education, emphasizing that the accuracy and readability of LLMs-generated content require further research and suggested to evaluate the performance of LLM-generated responses in patient education.

Malocclusion, with a prevalence of 43.5% to 67.2% in the general population [23], is recommended to use orthodontic treatment for intervention in the early stage [24,25]. The average treatment duration for orthodontic treatment is 27.9 months, and the number of appointments is 23.8 [26]. Although Zhao et al. [27] demonstrated that improved oral health knowledge enhances self-management (β = 0.527) and treatment outcomes (p < 0.001), the demand for health education remains high and unmet [28].

This study aims to systematically evaluate and compare the performance of LLMs and mainstream search engines in responding to common orthodontic questions, and to assess the suitability of LLMs as tools for patient education and self-management.

## Methods

This study was reviewed and approved by Peking University Institutional Review Board (Approval No: IRB00001052–24162). All procedures performed in studies involving human participants were in accordance with the ethical standards of the institutional research committee. Written informed consent was obtained from all individual participants prior to their inclusion in the study. Four expert raters provided written informed consent before participation and received no financial compensation. The participant recruitment and data collection period spanned from December 20, 2024, to January 5, 2025. This study followed the STROBE (Strengthening the Reporting of Observational Studies in Epidemiology) reporting guideline.

### Common orthodontic questions (45 questions, 6 categories) and prompt (1)

#### Question pool development (117 questions).

**Fig 1:**
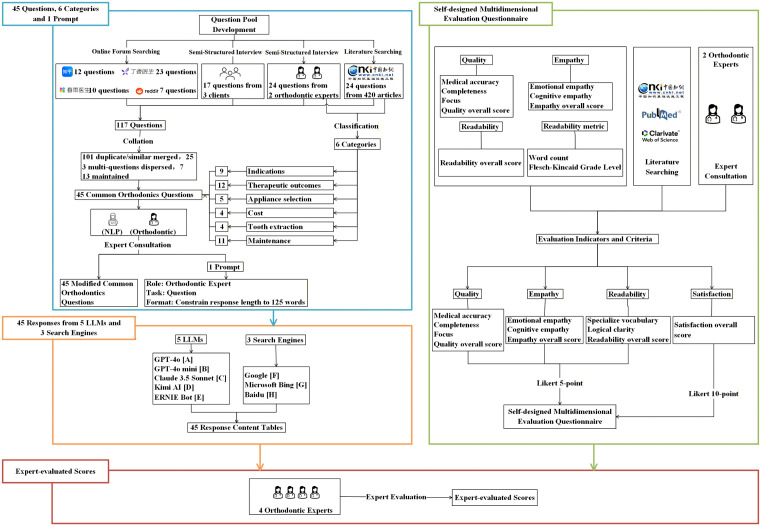
The flow chart of research design.

The China National Knowledge Infrastructure (CNKI), the largest Chinese academic database, was searched with the subject terms ‘Orthodontics’ and ‘Orthodontic Treatment’, and 420 articles were read by researchers and 24 common orthodontic questions were collected based on the principle of saturation (Fig 1).

In the four high-traffic online platforms, Zhihu (https://www.zhihu.com/), DXY (https://dxy.com/), Chunyu Health (https://www.chunyuyisheng.com/), and Reddit (https://www.reddit.com/), with “orthodontics” as the key word, and based on the principle of saturation, the researcher collected 52 common orthodontic questions.

Semi-structured interview (S1 Appendix) of two orthodontics experts and three orthodontic patients (Basic information see S2 Appendix) were conducted and 41 common orthodontic questions were collected based on the principle of saturation.

#### Common orthodontic questions (45 questions, 6 categories) and prompt (1).

Two researchers independently collated the questions, merging duplicates and splitting multi-part items. Disagreements were resolved by consulting a third researcher. The 117 questions were collated into 45 common orthodontic questions (S3 Appendix). To ensure content validity and relevance, four orthodontic experts reviewed the final 45 questions, achieving good inter-rater agreement (ICC = 0.80) and excellent overall content validity (S-CVI/Ave = 0.93). Orthodontic questions were categorized into six categories based on clinical consultation through literature search and expert consultation: indications, therapeutic outcomes, appliance selection, cost, tooth extraction, and maintenance.

Through interviews with an NLP expert (specializing in LLMs) and an orthodontic expert, as well as references [29,30], optimized response length limits (approximately 125 words), modified 45 questions, and determined one prompt(RTF: Role: “You are an orthodontic specialist.”, Task: “Please provide an accurate and comprehensible answer to the following patient question: Under what circumstances is orthodontic treatment necessary?”(Question 1), Format: “Answer in Chinese within approximately 125 words.”). All AI-generated responses were reviewed by four orthodontic experts under researcher supervision to ensure factual accuracy and compliance with AI research ethics principles.

### 45 responses from 5 LLMs and 3 search engines for 45 common orthodontic questions

Responses were collected on December 22, 2024, within a single day to ensure consistency and reduce the potential impact of subsequent system updates. We selected the five most popular LLMs and the top three search engines supporting Chinese-language interaction. All 45 questions were formulated in Chinese and presented to five LLMs—GPT-4o [A] (OpenAI; released May 2024; API version 2024-11-20), GPT-4o mini [B] (OpenAI; released July 2024; API version 2024-11-20), Claude 3.5 Sonnet [C] (Anthropic; model version claude-3–5-sonnet-20241022; released October 22, 2024), Kimi AI [D] (Moonshot AI; accessed December 2024), and ERNIE Bot [E] (Baidu; version 4.0; accessed December 2024)—and three mainstream search engines—Google [F], Microsoft Bing [G], and Baidu [H] (all accessed December 22, 2024). Each question was submitted to a new LLM chat session with a standardized prompt, while the top-ranked search engine result was used for comparison. The response content table (S4 Appendix) was obtained. All original Chinese materials was translated by two bilingual domain experts with clinical and linguistic backgrounds, following a forward-backward translation and recall process commonly adopted in international scale adaptation studies (S1 Appendix, S3 Appendix-S7 Appendix). No major discrepancies were identified between the forward and backward translations.

### Self-designed multidimensional evaluation questionnaire

A multidimensional evaluation questionnaire was developed based on existing tools, literature review, and expert input. The 4 primary indicators and 11 items were quality (medical accuracy, completeness, focus, overall quality scores), empathy (cognitive empathy, emotional empathy, overall empathy scores), readability (specialize vocabulary, logical clarity, overall readability score) and satisfaction (overall satisfaction scores). Cognitive empathy appears in phrases such as “This may be what you’re trying to understand”, while emotional empathy appears in phrases like “I understand this may worry you”. Detailed item definitions are provided in Supplementary File S5 Appenidix. A 5-point Likert scale was used for quality, empathy, and readability, and a 10-point Likert scale was used for satisfaction (S6 Appendix).

To ensure the reliability and validity of the self-designed multidimensional evaluation questionnaire, four orthodontic experts evaluated item relevance, clarity, and comprehensiveness (average CVI = 0.92). A pilot test of 20 responses was conducted, and internal consistency was assessed using Cronbach’s α after rescaling all 5-point and 10-point items to a common 5-point metric by dividing 10-point scores by two (e.g., 8 became 4). The questionnaire showed excellent overall reliability (α = 0.95), with subscale α values of 0.86 for quality, 0.86 for empathy, 0.79 for readability, and 0.64 for satisfaction.

### Expert-evaluated scores

Four experts (Basic information see S2 Appendix) were selected from the Department of Dentistry (each with >10 years of clinical experience in orthodontics) to evaluate responses generated by 5 LLMs and 3 search engines across 11 predefined evaluation indicators. Each indicator was independently rated by 4 evaluators and averaged to determine a consensus score. All evaluators were blinded to the identity of the models during scoring to minimize potential bias.

### Statistical analyses

The intraclass correlation coefficient (ICC) was calculated using a two-way mixed-effects model [ICC(C,1)] to assess overall inter-rater consistency across all evaluation items. Consensus scores for each item were computed as the mean of all raters’ scores. The strength of agreement was interpreted as poor (<0.50), moderate (0.50–0.75), good (0.75–0.90), or excellent (>0.90), following Koo and Li [31].Continuous variables were summarized as median (interquartile range, IQR), and nonparametric tests were employed due to violations of both normality and homogeneity of variance assumptions. The Kruskal-Wallis H test (two-sided) was applied to assess multi-group differences in indicator scores among five LLM models and multi-group differences in indicator scores among three search engines. To verify robustness, Friedman tests using question ID as a repeated-measures factor were additionally performed for LLMs (A-E) and search engines (F-H). For statistically significant findings (*p* < 0.05), post hoc multiple comparisons were conducted using the Mann-Whitney U test (two-sided) with Bonferroni corrections applied to adjust for multiple comparisons (p_adj < 0.05 considered significant), which was also applied to assess overall group differences between LLMs and search engines on the 11 indicators and question categories, as well as scores for each question. All statistical analyses were performed using R software (version 4.4.2), while the heatmap was generated using Manus.

### Temporal reproducibility and dynamic model behavior

Because LLMs and search engines are continuously updated, their outputs may vary over time. To reduce temporal variability, all responses were collected on a single day. However, the findings reflect model performance only at that time point (December 22, 2024), and reproducibility beyond this date may not be guaranteed. To support future replication, the full anonymized dataset is archived in Supplementary File S14 Appendix, and re-evaluation after major model updates is recommended.

## Results

ICC was calculated using a two-way mixed model and indicated moderate inter-expert agreement (ICC = 0.68, 95% CI [0.67, 0.69]), a level generally considered acceptable for studies involving subjective expert ratings.

**Fig 2:**
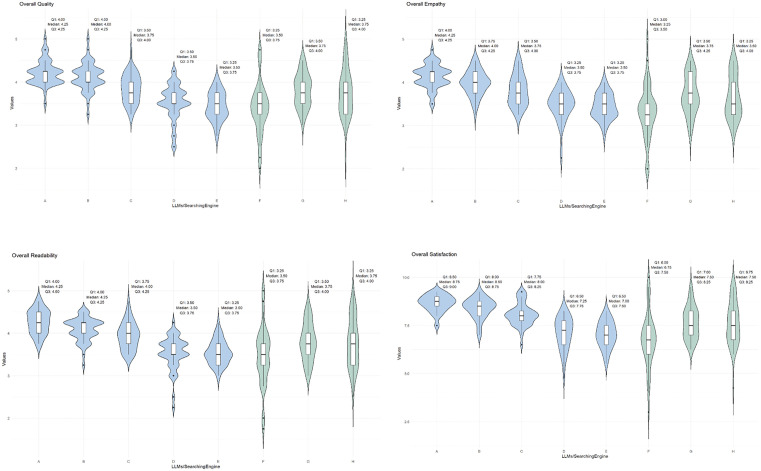
Overall Quality (A), Overall Empathy (B), Overall Readability (C), and Overall Satisfaction(D) of LLMs and search engine responses to questions. A indicates GPT-4o; B, GPT-4o mini; C, Claude 3.5 Sonnet; D, Kimi AI; E, ERNIE Bot; F, Google; G, Microsoft Bing; and H, Baidu. The midline indicates the median (50% percentile); the box, the 25% and 75% percentiles; and the density distribution plot represents the probability density of the response score distribution. Kruskal-Wallis tests were used for comparisons among LLMs and among search engines, and Mann-Whitney U tests were used for comparisons between LLMs and search engines.

Across the four dimensions (quality, empathy, readability, and satisfaction), GPT-4o[A] and GPT-4o mini[B] consistently outperformed other models, while Kimi AI, ERNIE Bot, and Google ranked lowest overall. The relative rankings across dimensions remained stable, highlighting consistent performance patterns among the models (Fig 2A-D, and S8 Appendix).

The LLMs scored significantly higher than the search engine in quality, empathy, readability, and satisfaction (all *p* < 0.001) (S9 Appendix). The best-performing LLMs, GPT-4o[A], achieved the highest median scores with relatively narrow IQRs compared with other LLMs in overall measures of quality (median: 4.25, IQR: 4.00 ~ 4.25), empathy (median: 4.25, IQR: 4.00 ~ 4.25), readability (median: 4.25, IQR: 4.00 ~ 4.50), and satisfaction (median: 8.75, IQR: 8.50 ~ 9.00) (Table 1, Bonferroni-adjusted p-values in S10 Appendix). Among LLMs, effect sizes were in the large range (η² ≈ 0.40–0.57) across all indicators.

**Table 1:** Quality, Empathy, Readability, and Satisfaction Scores of LLMs and search engine responses to questions.

	Response, median (IQR)										
	A	B	C	D	E	F	G	H	p-value1	p-value2	p-value3
Rater-evaluated quality score											
Medical accuracy	4.50(4.25, 4.75)	4.25(4.00, 4.50)	4.00(3.75, 4.25)	3.50(3.25, 4.00)	3.50(3.25, 4.00)	3.50(3.00, 3.75)	3.75(3.25, 4.25)	3.75(3.50, 4.00)	<0.001	<0.001	<0.001
Completeness	4.25(4.00, 4.50)	4.00(3.75, 4.25)	4.00(3.75, 4.00)	3.50(3.25, 3.75)	3.50(3.25, 3.75)	3.25(2.75, 3.50)	3.75(3.50, 4.25)	3.75(3.25, 4.00)	<0.001	<0.001	<0.001
Focus	4.25(4.00, 4.50)	4.00(4.00, 4.25)	4.00(3.75, 4.25)	3.75(3.50, 4.00)	3.50(3.25, 3.75)	3.50(3.00, 3.75)	3.75(3.50, 4.00)	3.75(3.25, 4.25)	<0.001	<0.001	0.068
Quality overall score	4.25(4.00, 4.25)	4.00(4.00, 4.25)	3.75(3.50, 4.00)	3.50(3.50, 3.75)	3.50(3.25, 3.75)	3.50(3.25, 3.75)	3.75(3.50, 4.00)	3.75(3.25, 4.00)	<0.001	<0.001	<0.001
Rater-evaluated empathy score											
Emotional empathy	4.00(4.00, 4.25)	4.00(4.00, 4.25)	4.00(3.75, 4.00)	3.50(3.50, 3.75)	3.50(3.25, 3.50)	3.50(3.00, 3.75)	3.75(3.25, 4.00)	3.75(3.50, 4.00)	0.004	<0.001	0.007
Cognitive empathy	4.25(4.00, 4.50)	4.00(4.00, 4.25)	3.75(3.75, 4.25)	3.75(3.50, 3.75)	3.50(3.25, 3.75)	3.50(3.00, 3.75)	4.00(3.50, 4.00)	3.75(3.50, 4.00)	0.007	<0.001	<0.001
Empathy overall score	4.25(4.00, 4.25)	4.00(3.75, 4.25)	3.75(3.50, 4.00)	3.50(3.25, 3.75)	3.50(3.25, 3.75)	3.25(3.00, 3.50)	3.75(3.50, 4.25)	3.50(3.25, 4.00)	<0.001	<0.001	0.002
Rater-evaluated readability score											
Specialize vocabulary	1.75(1.50, 2.00)	1.75(1.75, 2.00)	2.00(1.75, 2.25)	2.50(1.75, 2.25)	2.50(2.25, 2.50)	2.50(2.25, 2.75)	2.25(2.00, 2.50)	2.25(2.00, 2.75)	<0.001	<0.001	0.038
Logical clarity	4.25(4.00, 4.25)	4.00(4.00, 4.25)	4.00(3.75, 4.25)	3.50(3.25, 3.75)	3.50(3.25, 3.75)	3.50(3.25, 3.75)	3.75(3.50, 4.00)	3.75(3.50, 4.25)	<0.001	<0.001	0.006
Readability overall score	4.25(4.00, 4.50)	4.25(4.00, 4.25)	4.00(3.75, 4.25)	3.50(3.50, 3.75)	3.50(3.25, 3.75)	3.50(3.25, 3.75)	3.75(3.50, 4.00)	3.75(3.25, 4.00)	<0.001	<0.001	0.038
Rater-evaluated satisfaction score											
Satisfaction overall score	8.75(8.50, 9.00)	8.50(8.00, 8.75)	8.00(7.75, 8.25)	7.25(6.50, 7.75)	7.00(6.50, 7.50)	6.75(6.00, 7.50)	7.50(7.00, 8.25)	7.50(6.75, 8.25)	<0.001	<0.001	<0.001

Sample size per cell: n = 45 × 4 raters. p-value1: conducting statistical significance tests on the score differences between LLMs and search engines; p-value2: conducting statistical significance tests on the score differences among different LLMs; p-value3: conducting statistical significance tests on the score differences among search engine platforms. Effect sizes were calculated to supplement p-values and are summarized in S11 Appendix. Effect sizes were interpreted according to Cohen’s benchmarks (r ≈ 0.10, 0.30, 0.50 and η² ≈ 0.01, 0.06, 0.14 for small, medium, and large effects, respectively). Friedman tests aligned with Kruskal-Wallis for 9 of the 11 indicators; only specialized vocabulary and overall readability differed for search engines (S12 Appendix), and the overall performance patterns remained consistent.

As shown in Table 2, LLMs significantly outperformed search engines (*p* < 0.05) in most dimensions for “Cost” questions and in medical accuracy, completeness, focus, readability, and satisfaction for the “Appliance Selection” category. For the “Therapeutic Outcomes” category, LLMs also showed significant advantages in medical accuracy, quality, and satisfaction (*p* < 0.05). No significant differences were observed in other categories.

**Table 2:** Comparisons between LLMs and search engines scores on the indicators for the different question categories.

	Therapeutic Outcomes			Appliance Selection			Cost		
	LLMs	Search Engine	p-value	LLMs	Search Engine	p-value	LLMs	Search Engine	p-value
Rater-evaluated quality score									
Medical accuracy	4.075	3.670	0.006	4.100	3.580	0.036	4.000	3.290	0.029
Completeness	3.850	3.750	0.154	4.050	3.500	0.021	3.775	3.210	0.029
Focus	3.900	3.875	0.434	4.050	3.580	0.036	3.825	3.170	0.029
Quality overall score	3.875	3.790	0.045	4.000	3.830	0.114	3.750	3.295	0.028
Rater-evaluated empathy score									
Emotional empathy	3.825	3.750	0.339	3.900	3.500	0.056	3.750	3.250	0.059
Cognitive empathy	3.800	3.710	0.486	3.900	3.670	0.036	3.800	3.250	0.029
Empathy overall score	3.850	3.710	0.087	3.850	3.420	0.020	3.700	3.125	0.027
Rater-evaluated readability score									
Specialize vocabulary	2.075	2.250	0.077	2.100	2.330	0.059	2.100	2.670	0.028
Logical clarity	3.850	3.830	0.562	3.950	3.750	0.114	3.800	3.335	0.028
Readability overall score	3.925	3.750	0.077	3.900	3.670	0.059	3.900	3.330	0.028
Rater-evaluated satisfaction score									
Satisfaction overall score	8.100	7.455	0.019	8.350	7.420	0.012	7.525	6.335	0.027

**Fig 3:**
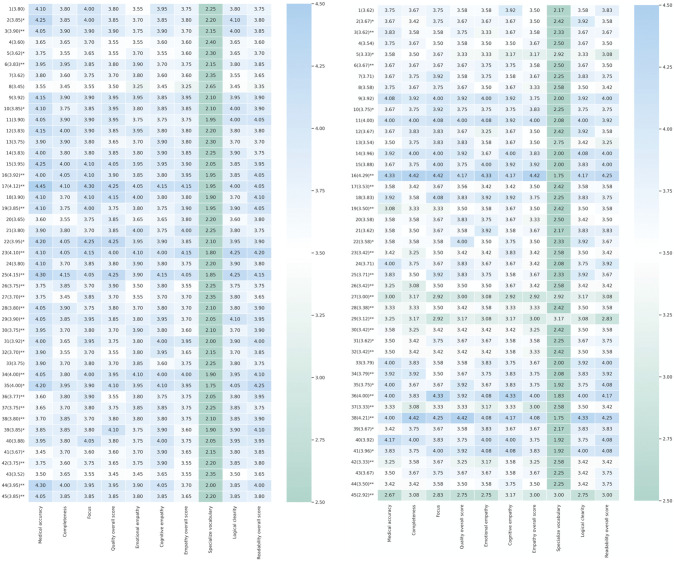
(A) Heat map of evaluation indicator scores for the LLMs. (B) Heat map of assessment evaluation indicator for the search engines. The horizontal axis lists the evaluation indicators, including medical accuracy, completeness, and so on; the vertical axis is the question number. Color shades indicate high or low scores, and color bars on the right side mark the range of scores. Median scores are shown in parentheses. Numerical data underlying this figure are provided in S13 Appendix.

As shown in Fig 3A, among the 45 questions addressed by LLMs, Question 17 (“Does orthodontic treatment cause dental caries?”) achieved the highest score of 4.12. The second highest-scoring question was Question 25 (“What types of orthodontic appliances are available?”) with 4.15, followed by Question 23 (“What preparations are needed before starting orthodontic treatment?”) scoring 4.10. Compared with search engine responses, the differences in scores for these questions were all statistically significant (p < 0.01).

As shown in Fig 3B, for the 45 search engine responses, search engines achieved higher scores than LLMs (4.29) on Question 16 (“How can orthodontic relapse be prevented after treatment?”). Question 36 (“How should oral hygiene be maintained during orthodontic treatment?”) ranked second with 4.00. The lowest score of 2.9 was observed for Question 45 (“Can orthodontic treatment cause speech difficulties?”). Question 27 (“What is the cost of orthodontic treatment?”) received the second lowest score of 3.00. Questions 27–30, which fall under the cost category, all received significantly lower scores than those provided by LLMs (p < 0.01).

## Discussion

This study systematically compiled 45 common orthodontic questions across six categories through literature search, online forums, and semi-structured interviews. Utilizing a self-designed multidimensional evaluation questionnaire (quality, empathy, readability, satisfaction), we comparatively analyzed responses from 5 LLMs and 3 search engines.

The high expert ratings obtained by GPT-4o and GPT-4o mini in this study indicate their potential for generating high-quality responses to patient education questions. This aligns with prior research, GPT-4o outperformed ERNIE Bot and other models in answering health questions [32]. The results suggest that LLMs outperform search engines in response quality, empathy, readability, and satisfaction. Prior research found that LLMs achieved significantly higher scores in comprehensiveness and quality compared to search engines (*p* < 0.05), when evaluated on patient queries about common chronic conditions [33]. LLMs have also outperformed human physicians on critical care questions, achieving accuracy scores as high as 93.3%, compared to an average physician score of 61.9% (*p* < 0.001) [34]. The higher expert ratings received by GPT-4o suggest its potential to provide comparatively higher-quality responses than the other LLMs evaluated in this study. In nutrition-related tasks, GPT-4o achieved an accuracy of 94.5%, outperforming Claude 3.5 Sonnet (92.7%) [35]. In radiology report evaluations, GPT-4o showed significantly higher agreement with expert radiologists (κ = 0.72 vs. κ = 0.15; *p* < 0.05), compared to other LLMs [36].

In contrast to traditional retrieval-based search engines, LLMs are generative, allowing them to synthesize information and produce logically consistent and highly readable responses [37]. LLMs’ deep learning architecture supports contextual understanding and empathetic expression, and is typically trained using vast corpora of human-generated text that include clinical guidelines [38], which may explain their relatively strong performance in providing accurate and complete responses. Observed performance differences between models may stem from differences in training datasets (including size, diversity, and specificity) as well as differences in algorithm design [39]. The superior performance of GPT-4o could be attributed to an end-to-end multimodal architecture [40], which seamlessly integrates text, image, and audio inputs within a unified Transformer framework, enhancing cross-modal alignment to generate more accurate, complete, and readable responses.

The expert-rated results indicate that the LLMs evaluated in this study have the potential to generate higher-quality responses for analytical reasoning questions (appliance selection and therapeutic outcomes) and for several cost-related questions. A benchmarking study [41] reports that LLMs achieve 100% appropriateness in responding to critical migraine treatment-related questions (e.g., “How is migraine treated?”, “I have a migraine, what will happen if I don’t treat it?”), demonstrating high accuracy in delivering guideline-aligned therapeutic information. According to research [42] published in *Mayo Clinic Proceedings: Digital Health* in 2024, LLMS showed high appropriateness compared to information site when answering questions in the category of ophthalmic appliance selection (e.g., “How should I decide which intraocular lens to choose?”). The significantly lower performance of search engines in answering cost-related orthodontic questions compared to LLMs may be attributed to their inability to model interdependent variables, which transformer-based LLMs effectively link through a non-linear attention mechanism [43]. Unlike the fragmented outputs of search engines, LLMs appear capable of integrating treatment time, aligner type, and geographic pricing to generate more realistic costing results.

Incorporating principles from Self-Determination Theory, LLMs could potentially support patients in setting personalized health goals and providing stage-based guidance to enhance autonomy and motivation [44]. Future health educators may increasingly shift their core responsibilities to “human-machine collaboration”, guiding patients from passively receiving information to actively using LLMs for self-management.

During expert review, occasional factual inaccuracies were identified, typically involving minor explanatory details, and occurred in fewer than 4% of the evaluated responses. Although LLM responses show high readability and apparent accuracy, they may still contain factual or contextual inaccuracies [45]. Professional oversight remains essential to ensure the reliability of health information.

The limitations of this study include the lack of patient raters to measure response empathy and satisfaction, and the reliance on expert-only validation may introduce potential scoring bias in these indicators. Future studies should integrate patient-reported evaluations to better capture subjective experiences and preferences, which are critical for evaluating health education tools in real-world contexts. Our study demonstrated moderate inter-rater consistency, reflecting variability in subjective expert evaluations. Despite blinding and standardized scoring, future studies should involve more diverse raters to enhance reliability. The rapid evolution of LLMs, with data collected in December 2024, limits temporal validity. Future replication using updated, version-controlled APIs is needed to verify model stability. A combined API–UI approach may better balance reproducibility with ecological validity in subsequent studies.

## Conclusion

In this cross-sectional study, LLMs, particularly GPT-4o, demonstrated superior performance compared to search engines in expert evaluations, suggesting potential usefulness for orthodontic patient education and self-management.

## Supporting information

S1 Appendix Expert interview protocol. (PDF)

S2 Appendix Basic Information for Clients and Experts. (PDF)

S3 Appendix 45 Common Orthodontic Questions. (PDF)

S4 Appendix Response Content Table (Sample). (PDF)

S5 Appendix Evaluation Indicators and Criteria. (PDF)

S6 Appendix Self-designed multidimensional evaluation questionnaire. (PDF)

S7 Appendix Original Chinese-language model outputs generated by five LLMs and three search engines. (PDF)

S8 Appendix Medical accuracy (A), Completeness (B), Focus (C), Emotional Empathy (D), Cognitive Empathy (E), specialized vocabulary (F), and Logical Clearity (G) of LLMs and search engine responses to questions. A indicates GPT-4o; B, GPT-4o mini; C, Claude 3.5 Sonnet; E, Kimi AI; F, ERNIE Bot; F, Google; G, Microsoft Bing; and H, Baidu. The midline indicates the median (50% percentile); the box, 25% and 75% percentile; the whiskers, 5% and 95% percentile; and the density distribution plot represents the probability density of the response score distribution.
(PDF)

S9 Appendix Quality, Empathy, Readability, and Satisfaction Scores of LLMs and search engine responses to questions. p-value: conducting statistical significance tests on the score differences between LLMs and search engines.
(PDF)

S10 Appendix Post hoc pairwise comparisons with Bonferroni correction following Kruskal–Wallis tests. (XLSX)

S11 Appendix Effect sizes for Kruskal-Wallis and Mann-Whitney U tests. (XLSX)

S12 Appendix Friedman tests. (XLSX)

S13 Appendix Numerical data underlying figures 3, including all heatmap cell values used for figure visualization. (XLSX)

S14 Appendix The minimal anonymized data set. (XLSX)

S15 Appendix R script used for data analysis. (R)

S16 Appendix Summary of Supplementary Files. (PDF)
