# Supplementary material for: Comparing large language models and search engine responses to common orthodontic questions
Source: PLoS One. 2026 Jan 2;21(1):e0339908. doi: 10.1371/journal.pone.0339908 (PMC12758715; doi:10.1371/journal.pone.0339908)
Supplement: S1 Appendix — (PDF) [file pone.0339908.s001.pdf]

Expert Interview Protocol

Client Version

1. Why did you decide to have orthodontic treatment in the first place? What were your biggest concerns?
2. What things were different from what you expected during the treatment? (e.g., pain, frequency of follow-up appointments, difficulty of care)
3. Looking back now, what do you wish you had known in advance that you know now?

Expert Version

1. What are the most common questions asked by patients during counselling? (in order of frequency)
2. What do you find to be the biggest misconceptions patients have about orthodontic results?
3. Which oral care recommendations are the most difficult for patients to follow?
4. If you could give patients only one piece of advice, what would you emphasize?
