# Supplementary material for: Comparing large language models and search engine responses to common orthodontic questions
Source: PLoS One. 2026 Jan 2;21(1):e0339908. doi: 10.1371/journal.pone.0339908 (PMC12758715; doi:10.1371/journal.pone.0339908)
Supplement: S2 Appendix — (PDF) [file pone.0339908.s002.pdf]

Basic Information for Clients and Experts

Clinical Professionals	Age	Title	Experience	Patient Advocate	Age	Have done/ Doing
C1(interviewee and evaluator)	52	Associate Chief Physician	25 years	P1	22	Have done orthodontic treatment
C2(interviewee and evaluator)	45	Senior Registered Nurse	20 years	P2	20	Have done orthodontic treatment
C3(evaluator)	22	Registered Nurse	21 years	P3	19	Doing orthodontic treatment
C4(evaluator)	19	Senior Registered Nurse	18 years			

AI Practitioners	Age	Affiliation	Domain	Experience
A1	38	Academia	LLMs	14 years

Note: C1 and C2 were interviewees, and C1, C2, C3, and C4 were evaluators.
