# Supplementary material for: Comparing large language models and search engine responses to common orthodontic questions
Source: PLoS One. 2026 Jan 2;21(1):e0339908. doi: 10.1371/journal.pone.0339908 (PMC12758715; doi:10.1371/journal.pone.0339908)
Supplement: S3 Appendix — (PDF) [file pone.0339908.s003.pdf]

45 Common Orthodontic Questions

1. Indications
1. Under what circumstances is orthodontic treatment necessary?
2. What are the factors influencing facial morphology?
3. What is the principle behind orthodontic treatment?
4. Are there age restrictions for orthodontic treatment? What is the optimal age for orthodontic treatment?
5. Can adults undergo orthodontic treatment?
6. Is it necessary to wait until all permanent teeth have erupted before starting orthodontic treatment?
7. How does orthodontic treatment differ between adults and adolescents?
8. What are the advantages and disadvantages of orthodontic treatment for adults?
9. Does orthodontic treatment affect a child's height development?
2. Therapeutic Outcomes
10. What are the benefits of orthodontic treatment?
11. Is orthodontic treatment solely for aesthetic purposes?
12. What are the risks associated with orthodontic treatment?
13. What are some common misconceptions about orthodontic treatment?
14. What are the consequences of delaying necessary orthodontic treatment?
15. Can orthodontic relapse occur after treatment?
16. How can orthodontic relapse be prevented after treatment?
17. Does orthodontic treatment cause dental caries?
18. Can orthodontic treatment lead to tooth mobility?
19. Is orthodontic treatment painful?
20. What should be done if gaps remain after orthodontic treatment?
21. What changes in facial appearance can be expected after orthodontic treatment?
3. Appliance Selection
22. What methods are available for orthodontic treatment?
23. What preparations are needed before starting orthodontic treatment?
24. Is it possible to perform orthodontic treatment on only one arch?
25. What types of orthodontic appliances are available?
26. Which is better for orthodontic treatment: clear aligners or traditional braces?
4. Cost
27. What is the cost of orthodontic treatment?
28. Is a higher cost indicative of better orthodontic treatment?
29. How long does orthodontic treatment typically take?
30. What are the consequences of discontinuing orthodontic treatment midway due to lack of time for follow-up visits?
5. Tooth Extraction
31. Is tooth extraction necessary for orthodontic treatment?
32. What are the implications of not extracting teeth when recommended by the orthodontist?
33. What risks are associated with tooth extraction for orthodontic treatment?
34. Can tooth extraction affect other teeth?

6. Maintenance
35. What precautions should be taken during orthodontic treatment?
36. How should oral hygiene be maintained during orthodontic treatment?
37. What are the cleaning methods for orthodontic appliances during treatment?
38. How should toothpaste and brushing frequency be chosen for effective oral hygiene during orthodontic treatment?
39. How can enamel demineralization be avoided during orthodontic treatment?
40. What dietary considerations should be taken during orthodontic treatment?
41. How should retainers be properly worn?
42. How long should retainers be worn?
43. What precautions should be taken when wearing retainers?
44. What are the implications of pregnancy during orthodontic treatment?
45. Can orthodontic treatment cause speech difficulties?
