# Supplementary material for: Comparing large language models and search engine responses to common orthodontic questions
Source: PLoS One. 2026 Jan 2;21(1):e0339908. doi: 10.1371/journal.pone.0339908 (PMC12758715; doi:10.1371/journal.pone.0339908)
Supplement: S4 Appendix — (PDF) [file pone.0339908.s004.pdf]

Response Content Table (Sample)

3.What is the principle behind orthodontic treatment?	
A	The principle of orthodontic treatment is to apply appropriate and continuous force through orthodontic devices, triggering biomechanical reactions in teeth and surrounding alveolar bone. Under external force, the alveolar bone on the pressure side gradually resorbs, while new bone tissue forms on the tension side, allowing teeth to move to ideal positions. Additionally, the periodontal ligament plays a crucial role by cushioning force and transmitting signals to promote bone remodeling. Orthodontists must design treatments precisely to ensure safe, effective movement direction, force intensity, and speed while avoiding side effects like root resorption or periodontal damage.
B	The principle of orthodontic treatment involves applying gentle, continuous force to move teeth into correct positions. Appliances (e.g., braces) use components like metal brackets, archwires, and elastic bands to adjust force direction and intensity regularly, gradually shifting teeth. Under constant pressure, surrounding bone undergoes remodeling—a process called bone reconstruction. During movement, alveolar bone resorbs on one side while new bone forms on the opposite side, securing teeth in place. Regular check-ups ensure progressive alignment.
C	Orthodontics primarily relies on bone remodeling principles. Continuous force causes osteoclasts to resorb alveolar bone on the pressure side and osteoblasts to form new bone on the tension side. Appliances (e.g., braces, clear aligners) generate sustained force to slowly move teeth within the alveolar bone. This process involves periodontal ligament reorganization, gradually positioning teeth correctly. Treatment typically takes 1-2 years to ensure stable movement and prevent tissue damage.
D	By applying external force via archwires to push/pull teeth in specific directions, pressure alters blood supply in the periodontal ligament. This triggers a biological response: bone forms via osteoblasts on one side while being resorbed by osteoclasts on the other, enabling bone remodeling.
E	Orthodontic treatment primarily uses appliances to apply force—similar to physical action-reaction principles. Brackets bonded to teeth transmit force, guiding movement toward an aligned dental arch. The maxilla and mandible are uniquely capable of lifelong remodeling. Pre-treatment X-rays assess overall dental health; severe periodontal disease may affect tooth mobility during treatment, requiring case-specific evaluation.
F	Orthodontics applies sustained, gentle force to gradually align teeth within the alveolar bone. Between teeth and bone lies tissue that can be resorbed and rebuilt. Force from appliances causes alveolar bone resorption on one side and reconstruction on the other, facilitating movement. This process takes months to years.
G	The principle involves using external force to guide tooth and alveolar bone movement/remodeling, adjusting tooth positions and occlusion. Appliances apply force; tooth movement occurs via periodontal ligament deformation and bone remodeling, achieving proper alignment and bite.
H	Orthodontic correction relies on force interaction and alveolar bone remodeling. Teeth are harder than alveolar bone. When misaligned, external pressure can move them directionally within the bone. Force must be controlled: insufficient force fails to move teeth, while excessive force risks bone resorption. Seek treatment at certified hospitals for safety. Maintain oral hygiene—brush and rinse after eating.
