# Supplementary material for: Comparing large language models and search engine responses to common orthodontic questions
Source: PLoS One. 2026 Jan 2;21(1):e0339908. doi: 10.1371/journal.pone.0339908 (PMC12758715; doi:10.1371/journal.pone.0339908)
Supplement: S5 Appendix — (PDF) [file pone.0339908.s005.pdf]

Evaluation Indicators and Criteria

Primary Indicator	Secondary Indicator	Definition	Likert 5-Point Scale Scoring Instructions
**Quality**	Medical accuracy	The correctness and consistency of the information provided with scientific knowledge and clinical guidelines.	• 1: Completely inaccurate<br>• 2: Inaccurate<br>• 3: Moderate<br>• 4: Accurate<br>• 5: Completely accurate
	Completeness	Measures whether the response covers all relevant aspects of the question.	• 1: Very incomplete<br>• 2: Incomplete<br>• 3: Moderate<br>• 4: Complete<br>• 5: Very complete
	Focus	The extent to which the response addresses all parts of the patient's question and includes only relevant information.	• 1: Completely irrelevant<br>• 2: Irrelevant<br>• 3: Moderate<br>• 4: Relevant<br>• 5: Completely relevant
	Overall Quality Score	The overall score reflects the evaluator's preference for the response.	• 1: Very poor quality<br>• 2: Poor quality<br>• 3: Moderate quality<br>• 4: High quality<br>• 5: Very high quality
**Empathy**	Emotional Empathy	The extent to which the response shares the patient's emotional experience.	• 1: Very poor emotional empathy<br>• 2: Poor emotional empathy<br>• 3: Moderate emotional empathy<br>• 4: High emotional empathy<br>• 5: Very high emotional empathy
	Cognitive Empathy	The extent to which the response represents the patient's mental state and their views on the proposed plan.	• 1: Very poor cognitive empathy<br>• 2: Poor cognitive empathy<br>• 3: Moderate cognitive empathy<br>• 4: High cognitive empathy<br>• 5: Very high cognitive empathy
	Overall Empathy Score	Evaluates whether the response demonstrates compassion, addresses the patient's concerns, and respects their values and preferences.	• 1: Very poor empathy<br>• 2: Poor empathy<br>• 3: Moderate empathy<br>• 4: High empathy<br>• 5: Very high empathy

**Readability**	Specialize vocabulary	Evaluates the amount of professional terminology used in the response.	• 1: Very little specialize vocabulary<br>• 2: Little specialize vocabulary<br>• 3: Moderate specialize vocabulary<br>• 4: Much specialize vocabulary<br>• 5: Very much specialize vocabulary
	Logical Clarity	Evaluates the logical structure of the response.	• 1: Very unclear logic<br>• 2: Unclear logic<br>• 3: Moderate<br>• 4: Relatively clear logic<br>• 5: Very clear logic
	Overall Readability Score	Evaluates the clarity of the response, including language usage and readability for the target audience.	• 1: Very poor readability<br>• 2: Poor readability<br>• 3: Moderate readability<br>• 4: High readability<br>• 5: Very high readability
**Satisfaction**	Overall Satisfaction Score	Experts' overall satisfaction score for the response.	1-10: 1 represents very dissatisfied, 10 represents very satisfied.
