# Supplementary material for: Comparing large language models and search engine responses to common orthodontic questions
Source: PLoS One. 2026 Jan 2;21(1):e0339908. doi: 10.1371/journal.pone.0339908 (PMC12758715; doi:10.1371/journal.pone.0339908)
Supplement: S6 Appendix — (PDF) [file pone.0339908.s006.pdf]

Self-designed Multidimensional Evaluation Questionnaire

**Q**	**XXXXXXXX**						
**A**	**Medical accuracy**	**Completeness**	**Focus**	**Overall Quality**	**Emotional Empathy**	**Cognitive Empathy**	**Overall Empathy**
	1 2 3 4 5	1 2 3 4 5	1 2 3 4 5	1 2 3 4 5	1 2 3 4 5	1 2 3 4 5	1 2 3 4 5
	**Specialize vocabulary**	**Logical clarity**	**Overall Readability**	**Overall Satisfaction**			
	1 2 3 4 5	1 2 3 4 5	1 2 3 4 5	1 2 3 4 5 6 7 8 9 10			
**B**	**Medical accuracy**	**Completeness**	**Focus**	**Overall Quality**	**Emotional Empathy**	**Cognitive Empathy**	**Overall Empathy**
	1 2 3 4 5	1 2 3 4 5	1 2 3 4 5	1 2 3 4 5	1 2 3 4 5	1 2 3 4 5	1 2 3 4 5
	**Specialize vocabulary**	**Logical clarity**	**Overall Readability**	**Overall Satisfaction**			
	1 2 3 4 5	1 2 3 4 5	1 2 3 4 5	1 2 3 4 5 6 7 8 9 10			
**C**	**Medical accuracy**	**Completeness**	**Focus**	**Overall Quality**	**Emotional Empathy**	**Cognitive Empathy**	**Overall Empathy**
	1 2 3 4 5	1 2 3 4 5	1 2 3 4 5	1 2 3 4 5	1 2 3 4 5	1 2 3 4 5	1 2 3 4 5
	**Specialize vocabulary**	**Logical clarity**	**Overall Readability**	**Overall Satisfaction**			
	1 2 3 4 5	1 2 3 4 5	1 2 3 4 5	1 2 3 4 5 6 7 8 9 10			
**D**	**Medical accuracy**	**Completeness**	**Focus**	**Overall Quality**	**Emotional Empathy**	**Cognitive Empathy**	**Overall Empathy**
	1 2 3 4 5	1 2 3 4 5	1 2 3 4 5	1 2 3 4 5	1 2 3 4 5	1 2 3 4 5	1 2 3 4 5
	**Specialize vocabulary**	**Logical clarity**	**Overall Readability**	**Overall Satisfaction**			
	1 2 3 4 5	1 2 3 4 5	1 2 3 4 5	1 2 3 4 5 6 7 8 9 10			
**E**	**Medical accuracy**	**Completeness**	**Focus**	**Overall Quality**	**Emotional Empathy**	**Cognitive Empathy**	**Overall Empathy**
	1 2 3 4 5	1 2 3 4 5	1 2 3 4 5	1 2 3 4 5	1 2 3 4 5	1 2 3 4 5	1 2 3 4 5
	**Specialize vocabulary**	**Logical clarity**	**Overall Readability**	**Overall Satisfaction**			
	1 2 3 4 5	1 2 3 4 5	1 2 3 4 5	1 2 3 4 5 6 7 8 9 10			
**F**	**Medical accuracy**	**Completeness**	**Focus**	**Overall Quality**	**Emotional Empathy**	**Cognitive Empathy**	**Overall Empathy**
	1 2 3 4 5	1 2 3 4 5	1 2 3 4 5	1 2 3 4 5	1 2 3 4 5	1 2 3 4 5	1 2 3 4 5
	**Specialize vocabulary**	**Logical clarity**	**Overall Readability**	**Overall Satisfaction**			
	1 2 3 4 5	1 2 3 4 5	1 2 3 4 5	1 2 3 4 5 6 7 8 9 10			
**G**	**Medical accuracy**	**Completeness**	**Focus**	**Overall Quality**	**Emotional Empathy**	**Cognitive Empathy**	**Overall Empathy**
	1 2 3 4 5	1 2 3 4 5	1 2 3 4 5	1 2 3 4 5	1 2 3 4 5	1 2 3 4 5	1 2 3 4 5
	**Specialize vocabulary**	**Logical clarity**	**Overall Readability**	**Overall Satisfaction**			
	1 2 3 4 5	1 2 3 4 5	1 2 3 4 5	1 2 3 4 5 6 7 8 9 10			
**H**	**Medical accuracy**	**Completeness**	**Focus**	**Overall Quality**	**Emotional Empathy**	**Cognitive Empathy**	**Overall Empathy**
	1 2 3 4 5	1 2 3 4 5	1 2 3 4 5	1 2 3 4 5	1 2 3 4 5	1 2 3 4 5	1 2 3 4 5
	**Specialize vocabulary**	**Logical clarity**	**Overall Readability**	**Overall Satisfaction**			
	1 2 3 4 5	1 2 3 4 5	1 2 3 4 5	1 2 3 4 5 6 7 8 9 10			
