# Supplementary material for: Comparing large language models and search engine responses to common orthodontic questions
Source: PLoS One. 2026 Jan 2;21(1):e0339908. doi: 10.1371/journal.pone.0339908 (PMC12758715; doi:10.1371/journal.pone.0339908)
Supplement: S7 Appendix — (PDF) [file pone.0339908.s007.pdf]

1.什么情况下需要做牙齿矫正？	
A	牙齿矫正适用于以下情况：第一，牙列不齐，如龅牙、地包天、牙缝过大等，影响外观和咬合功能；第二，咬合异常，可能导致咀嚼效率降低或咬合紊乱，引发颞下颌关节问题；第三，牙齿拥挤或排列不整齐，难以清洁，易导致龋齿和牙周疾病；第四，儿童时期发现不良口腔习惯（如咬指甲、吐舌）造成牙颌畸形，应及时干预。此外，有些成人希望通过矫正改善面部轮廓或提升自信。总之，牙齿矫正不仅为美观，更为口腔健康服务。<br>牙齿矫正通常在牙齿排列不齐、咬合不正（如地包天、龅牙）、间隙过大或过密、影响咀嚼或发音、导致面部不对称等情况下需要。此外，矫正有助于预防牙龈疾病和牙齿磨损，改善口腔健康。具体是否需要矫正，需由正畸医生根据个人口腔情况评估后决定。建议尽早咨询，以获得最佳治疗效果。
B	牙齿矫正通常适用于以下情况：首先，当牙齿排列不整齐，出现拥挤或间隙过大时，需要矫正。其次，牙齿咬合不正常，如上牙或下牙前突、咬合深或开合等，也需要矫正。此外，牙齿不整齐可能影响口腔卫生，导致清洁困难，增加蛀牙和牙龈疾病的风险。矫正还可以改善咀嚼功能，减少牙齿磨损及其他口腔问题。最后，严重的牙齿问题可能影响面部美观和自信心，因此，适时矫正能够改善外貌并提升自信。
C	需要进行牙齿矫正的主要情况包括：牙齿拥挤或间隙过大，导致咀嚼和发音受影响；牙齿排列不齐，影响美观和自信；上下颌咬合不当，可能引起颞下颌关节疾病；前牙反咬或深覆盖，影响正常咀嚼功能；龅牙（俗称"地包天"）或突牙，影响面部外观和发音。此外，某些先天性或后天性的颌面部发育异常，也需要通过正畸来改善。建议在青少年时期进行矫正效果最佳，但成年人同样可以进行，只是疗程可能相对更长。
D	1、牙齿不整齐、拥挤：前牙拥挤使人不敢开怀大笑，矫正牙齿能从美观、功能和身心健康上带来积极改变。 2、牙缝过大：大牙缝不仅影响美观，还易导致食物嵌塞和牙周病。 牙齿矫正能有效“关闭”牙缝，恢复健康咬合。 3、上颌前突、下颌后缩、龅牙：会使人觉得牙齿和嘴巴凸显，矫正治疗能显著改善外观和功能。
E	通常情况下牙齿稀疏、前后龅牙、反颌以及牙列拥挤等情况，需要进行牙齿矫正等治疗。如患者发生牙列拥挤等情况，患者牙齿的排列不但不容清洁，而且极其影响口腔卫生及美观。需要及时进行牙齿矫正。龅牙、反颌、偏颌等情况不但影响患者日常饮食咀嚼，也会造成患者面部畸形严重影响美观的问题的发生。需及时到专业口腔医院进行矫正治疗。矫正牙齿是一个长期的治疗过程，需要患者积极配合医嘱，保持自身口腔卫生，及时复诊已达到最好的牙齿矫正效果。
F	需要做牙齿矫正的情况主要包括：牙齿拥挤、牙列不齐、深覆合、反颌、开颌、偏颌、牙齿稀疏、龅牙等。这些情况不仅影响美观，还可能导致口腔健康问题，如牙龈出血、龋病和塞牙，以及影响咬合功能。
G	当出现牙齿错位、排列异常、牙弓形态异常、龅牙、偏颌，以及地包天、深覆合、锁颌等错颌畸形时，需要做牙齿矫正。牙齿矫正可以恢复牙齿的正常排列和功能，提升美观度，同时也有助于预防牙齿疾病。
H	以下两种情况需要牙齿矫正。<br>第一，错颌畸形，常见的有地包天，天包地，多生牙等。如果出现这些情况，需要进行牙齿矫正治疗。这些情况当中的70%需要进行治疗。另外30%可能畸形并不明显，只是有一些轻微的牙颌畸形，这种情况可以不用调整。<br>第二，牙列拥挤，这种情况表现出牙齿不齐，牙弓呈卵圆形的弧度。牙齿不齐的原因是由于颌骨退化，还有就是饮食精细导致牙齿的磨耗减少，以及摄糖量增加引起龋齿发生率增高，也可以导致牙齿牙列拥挤。

2.面型的影响因素有什么?	
A	面型的影响因素主要包括遗传、环境和功能因素。**遗传因素**决定了骨骼的基本形态，如颌骨发育和面部比例。**环境因素**包括饮食习惯、生活方式以及不良口腔习惯（如长期口呼吸、咬唇等），这些会影响面部肌肉和骨骼的发育。**功能因素**则涉及咀嚼、吞咽和语言习惯，这些动作能塑造面部肌肉和骨骼的动态平衡。此外，生长发育阶段的疾病或外伤也可能导致面型异常。面型的形成是多种因素综合作用的结果，需要综合考量来分析具体原因和影响。
B	面型的影响因素主要包括遗传、年龄、性别、生活习惯和环境因素。遗传因素决定了面部的骨骼结构和皮肤特征。随着年龄增长，皮肤弹性下降，面部轮廓可能发生变化。性别也会影响面型，男性通常拥有更宽的下颌骨和较为刚毅的面部线条，而女性则面部较为圆润。生活习惯如饮食、睡眠以及是否吸烟、饮酒等，都会影响面部的健康状况。此外，环境因素如气候变化和紫外线辐射等，也可能导致面部皮肤老化和松弛。
C	面型的影响因素主要包括以下几个方面：首先是遗传因素，父母的基因会决定面部骨骼结构和软组织特征；其次是年龄因素，随着年龄增长，面部脂肪、胶原蛋白流失会导致面型变化；再次是生活习惯，如饮食结构、作息时间、运动习惯等都会影响面部肌肉和脂肪分布；此外，内分泌水平、疾病状况也会影响面型；最后，面部表情习惯和长期的压力状态也会在一定程度上改变面型。
D	第一个方面，是我们面部的骨骼。它本身体积的大小以及它的空间位置决定了我们的脸型。比如说颧骨的大小是否过大？是否外突？有没有内后缩？颧弓是否外凸？下颌骨的整个形态是否好看？下颌骨的位置决定了我们的下巴是向前还是向后等等各方面。第二个方面，是我们面部的肌肉大小。比如咬肌，它就是影响我们面部宽度的一个重要因素。第三个方面，是面部的胖瘦。俗话说人一胖毁所有，这句话用在面部也是正确的。因为如果面部很胖，那不管多好看的脸型，都看不出来了，我们只看到脸上一堆肉。
E	这些都是影响脸型的重要因素。 颌骨 起了很重要的作用，决定了脸型的 基础结构。脸型可以通过不同的 分类方法 进行分类，正看和侧看脸型也是有区别的。 脸型的 分类方法 很多。 在我国古代的 绘画理论 和面相书中就有各种各样的分类法，并对脸型赋予了人格的内容。 下面是几种常见的脸型分类法：
F	面型受遗传、生活习惯、生长发育和环境因素影响。遗传因素决定了面部骨骼的基本形态；生活习惯如饮食、呼吸方式会影响肌肉和脂肪分布；生长发育阶段的营养状况和健康状况也会影响面型；环境因素如气候、紫外线照射等也有一定影响。
G	面型的影响因素主要包括面部骨骼的形状和大小、附着在骨骼上的软组织分布及其形状。其中，骨骼形成了面型的框架，而软组织则对面型起到了充实和修饰作用，如面部脂肪的分布和咬肌的形态等。此外，遗传、生活习惯等也可能对面型产生一定影响。
H	面型的影响因素主要包括遗传、骨骼结构和软组织三个方面。<br>遗传因素：面型的形成和变化在很大程度上受到遗传的影响。父母的面部特征会通过基因传递给子女，决定面部的基本轮廓和形态<br>骨骼结构：骨骼结构是面型的基础，特别是颌骨的形态对脸型有决定性作用。上颌骨、颧骨、颞骨、额骨和顶骨等构成了面部上半部的轮廓，而下颌骨则决定了面部下半部的形态。骨骼的发育和形态变化会直接影响面型<br>软组织：软组织的分布和厚度也会对面型产生影响。例如，面部的脂肪、肌肉和皮肤等软组织的变化可以改变面型的外观。随着年龄的增长，软组织的变化尤为明显，如脂肪减少、皮肤松弛等，这些都会导致面型的改变<br>生活习惯和环境因素：生活习惯和环境因素也会对面型产生一定影响。例如，咀嚼习惯、睡眠姿势等都会对面部的发育和形态产生微妙的影响。此外，牙齿的排列和咬合

	关系也会影响面型的整体美观和功能。
**3.牙齿矫正的原理是什么?**	
A	牙齿矫正的原理是通过正畸装置对牙齿施加适当而持续的力，促使牙齿及其周围的牙槽骨发生生物力学反应。牙齿受到外力后，压力侧的牙槽骨会逐渐吸收，而张力侧则新生骨组织，牙齿因此移动到理想位置。此外，牙齿周围的牙周膜也在矫正中起重要作用，通过缓冲力和信号传递促进骨改建。正畸需要医生精确设计，以确保牙齿移动的方向、力度和速度均安全有效，同时避免副作用如牙根吸收或牙周损伤。
B	牙齿矫正的原理是通过持续施加轻微的力量，使牙齿移动到正确的位置。矫正器（如牙套）通过金属托槽、弓丝和橡皮圈等组件，定期调整力的方向和强度，逐步推动牙齿。牙齿在不断的压力下，其周围的骨质会发生重塑，称为骨重建过程。在牙齿向目标位置移动的过程中，牙齿周围的牙槽骨会被溶解，而另一侧的骨组织则会重新形成，从而固定牙齿的位置。矫正过程中需要定期复诊，以确保牙齿逐步达到预期的排列效果。
C	牙齿矫正的原理主要基于骨组织重塑原理。当对牙齿施加持续的力时，压力端的牙槽骨会被破骨细胞吸收，而张力端会有成骨细胞形成新的骨组织。通过正畸装置(如牙套、隐形矫正器等)产生的持续性力量，牙齿在牙槽骨内缓慢移动。这个过程同时涉及牙周韧带的重组，使牙齿逐渐到达理想位置。整个过程需要时间，通常持续 1-2 年，以确保牙齿稳定移动并避免组织损伤。
D	借由牙弓线施加外力将牙齿推、拉向特定方向，并将压力加诸牙周韧带，牙周供血的改变所引发的生物性反应，透过一侧的骨头因造骨细胞产生，另一侧的骨头则被噬骨细胞再吸收，即可造成骨头的重塑。
E	牙齿矫正主要通过矫正器进行，此方法和物理上的作用力、反作用力相同，把托槽放在牙齿上，将力作用在牙齿上，使牙齿向着整齐排列的曲线移动。上颌骨和下颌骨是人体中唯一可以终身改建的骨头，通过推拉可以使其正常移动，符合正常、健康的移动。进行正畸之前应该拍摄 X 光片，对全口牙齿进行评判。如果患有比较严重的牙周疾病，在正畸的过程中可能会对牙齿的松动度造成一定的影响，需根据具体情况判断是否可以进行正畸处理。
F	牙齿矫正的原理是通过施加持续、温和的力量，使牙齿在牙槽骨中移动，逐渐排齐。牙齿与牙槽骨之间有一层可被吸收和重建的组织，矫正器施加的力量导致牙齿一侧的牙槽骨吸收，另一侧重建，牙齿随之移动。这个过程需要时间，通常几个月到几年不等。
G	牙齿矫正的原理是通过外力作用，引导牙齿和牙槽骨发生移动和改建，调整牙齿位置和咬合关系。外力由矫正器施加，牙齿移动通过牙周膜弹性形变和牙槽骨改建实现，最终达到牙齿排列整齐、咬合良好的目的。
H	校正牙齿的原理主要是与力的相互作用以及牙槽骨的改建。<br>牙齿的硬度是要高于牙槽骨的，如果牙齿出现了排列不齐的现象，通过外界施加压力的方式，牙齿是可以在牙槽骨内向各个方向进行移动的，通过施压的力量控制生理性移动的力量，但是要保证施加力量的力度，小了牙齿有可能不动，如果力度过大很可能会造成牙槽骨出现吸收的现象。在校正牙齿的时候需要到正规的医院操作，能够使安全性更高。<br>日常还应保持口腔的清洁卫生，在吃过食物之后及时的刷牙漱口。

4.牙齿矫正有年龄限制吗？牙齿矫正的最佳年龄是几岁?	
A	牙齿矫正并没有严格的年龄限制，儿童、青少年和成年人都可以进行矫正。不过，最佳年龄通常是 11 至 14 岁左右，因为此时恒牙已经长齐，颌骨仍在生长，矫正效果更快、更稳定。儿童在 7 岁左右可以首次检查牙齿发育情况，部分问题如严重咬合不正需及早干预。成年人虽然矫正时间可能较长，但同样可以通过隐形矫正器等技术获得理想效果。年龄不是决定因素，牙齿健康状况和正畸需求才是关键。<br>牙齿矫正没有严格的年龄限制，但最佳年龄通常在 12-16 岁，此时恒牙已基本长齐，骨骼仍具可塑性，矫正效果较好。成年人也可矫正，但时间可能更长。具体方案需由专业牙医评估。
B	牙齿矫正没有严格的年龄限制，但最佳矫正年龄通常是在青少年时期，约为 12 至 16 岁左右。这是因为此时牙齿和颌骨的生长尚未完全结束，矫正效果较好，治疗周期也较短。对于成年人，虽然矫正依然有效，但由于骨骼发育已基本完成，矫正可能需要更长时间，并可能需要配合其他治疗手段，如牙齿拔除或手术。不过，随着现代矫正技术的进步，成年人矫正牙齿的效果也有了很大改善。总之，任何年龄段的人都可以进行牙齿矫正，关键在于个体情况。
C	牙齿矫正没有严格的年龄限制，任何年龄段都可以进行正畸治疗。但最佳矫正时间是在 12-18 岁的青少年时期，因为这个阶段牙齿和颌骨还在生长发育，骨骼和牙周组织较为柔软，更容易移动和塑形，治疗效果更好，时间也相对较短。成年后也可以进行牙齿矫正，只是治疗周期可能会更长，且费用相对更高。建议在 6-9 岁时进行口腔检查，及早发现问题并制定治疗方案。需要注意的是，老年人由于牙周病风险增加，矫正时要更加谨慎。
D	通常最佳牙齿矫正年龄：儿童矫正建议 5-10 岁；青少年矫正 12-14 岁；成人矫正建议 18 岁以后矫正是比较理想的。
E	牙齿矫正是没有年龄限制的，但牙齿矫正最合适的时间就是青少年期，因为这个时候牙齿骨骼都没有发育完成，牙槽骨是代谢最活跃的时候，矫正起来相对来说比较容易，时间比较短，矫正效果也比较好。 但是 18 岁之后，随着牙齿和颌面部骨骼已经发育完成，矫正起来时间可能会相对长一些，尤其是成年人，可能口腔里的条件更复杂一些，会出现很多缺失牙、坏牙的情况，那么矫正起来难度也会大一些。
F	牙齿矫正没有严格的年龄限制，任何年龄都可以进行矫正。牙齿矫正的最佳年龄是 7 岁到 12 岁之间。
G	牙齿矫正通常没有严格的年龄限制，牙周健康允许即可矫正。牙齿矫正的最佳年龄因个体差异而异，但一般认为在乳牙期（3-6 岁）、混合牙期（7-11 岁）和恒牙期（女孩 10-12 岁，男孩 11-13 岁）进行矫正效果最佳。
H	一般情况下做牙齿矫正是没有年龄限制的，如果牙齿不存在大量缺损或是口腔内不存在严重牙周疾病，那么年龄大于 12 岁的任何年龄段基本都是可以进行牙齿矫正的，不过不同年龄段做牙齿矫正时，在矫正时间、费用、牙齿保持等多种情况上都会存在差异。通常年龄越小矫正效果也就会越好，牙齿矫正的最佳年龄是 12-18 岁，此时是人体牙槽骨的最佳发育年龄，进行牙齿矫正能够达到最好的效果，而且后期佩戴保持器的时间也会相对较短，不会花费太多时间。任何年龄段都是可以进行牙齿矫正的，患者在矫正之前可以先前往正规医院咨询专业的口腔科医生，了解相关事项以及流程之后在医生指导下选择治疗方式。

5.成年人可以做牙齿矫正吗?	
A	成年人可以做牙齿矫正，而且如今的技术为成人提供了更多选择。尽管儿童和青少年时期是矫正牙齿的最佳阶段，但成年人同样可以通过矫正改善牙齿排列和咬合问题。常见的成人矫正方法包括传统金属牙套、陶瓷牙套和隐形矫正器。成年人矫正的时间可能略长，因骨骼发育已稳定，但效果依然显著。此外，矫正不仅提升美观，还能改善咀嚼功能和口腔健康。矫正前应咨询专业牙医，根据自身需求选择合适方案。健康的牙齿无关年龄，关键在于行动和坚持。
B	成年人是可以进行牙齿矫正的。尽管成年人已经完成了牙齿和颌骨的发育，但通过现代牙齿矫正技术，如隐形矫正、传统托槽等，仍然能够达到理想的矫正效果。成年人做牙齿矫正的优势是配合度较高，矫正过程中的口腔卫生和护理往往更为细致。但需要注意的是，成人的牙齿和颌骨可能会比青少年更为坚硬，因此矫正的时间可能会相对较长。牙医会根据个人的口腔状况设计最合适的矫正方案。
C	成年人完全可以进行牙齿矫正。虽然最佳矫正时间是在青少年期，但随着医疗技术的发展，成年人矫正牙齿已经非常普遍和安全。不过，成年人的牙齿矫正时间可能会比青少年更长，一般需要 1-3 年不等。目前有多种矫正方案可供选择，包括传统金属托槽、陶瓷托槽、舌侧矫正和隐形矫正等。选择合适的方案时，需要考虑个人的牙齿状况、预算和生活方式。建议先到正规医院进行专业检查，让医生根据具体情况制定个性化的矫正方案。<br>另外，成年人矫正时要特别注意口腔卫生，保持规律复查，遵医嘱进行调整。
D	牙齿矫正没有年龄限制。 接受这种牙科治疗的唯一要求是良好的口腔健康。
E	患者成年以后，还可以进行矫正牙齿，目前随着我国科学技术的发展，其成人正畸已经越来越成熟，其各种成人正畸的技术，也在不断地更新和发展。随着矫治器的发明和发展，对于成年人的正畸，目前比较趋于完善和成熟，因为目前很多成年人爱美，并且工作需要以及相关的各种原因，而导致成年人可能需要进行牙齿的排列，从而很好地改善其成年人的口腔颌面部的功能以及美观等。因此，患者在成年以后，还可以进行矫正牙齿，甚至对有些患者，必须要进行矫正牙齿以后，从而可以适应现在的生活及工作等。对于成年人矫正，建议患者尽早地到正规医院的口腔科，或者是口腔医院的口腔正畸科，找专业的口腔正畸的医生，进行仔细的检查和术前的评估，根据患者的错颌畸形的程度以及类型，而给予正确的正畸方案。
F	成年人完全可以做牙齿矫正。牙齿矫正并没有严格的年龄限制，成年人的牙齿矫正除了时间可能会长一些，效果上并没有太大影响。
G	成年人可以做牙齿矫正。只要牙周健康，成年人就可以进行牙齿矫正，只是所需时间可能比青少年长，因为成年人的牙槽骨改建速度相对较慢。成年人矫正牙齿时，可以根据自身情况选择适合的矫正方法，如隐形牙套、金属牙套等。
H	成人一般可以牙齿矫正，但若牙齿的健康状况较差，一般不能做牙齿矫正。建议去医院就诊，根据检查明确诊断，在确诊病因后，配合医生针对性治疗。<br>1、可以：如果牙齿的健康状况较好，牙齿排列不整齐的症状较轻，通常是可以做牙齿矫正的，但是需要选择正规的口腔科机构。2、不可以：如果成人的身体健康状况较差，患有多种基础疾病和严重的口腔疾病，做牙齿矫正的风险系数是相对比较高的，这种状态一下可能会影响做牙齿矫正的效果。<br>做牙齿矫正之前建议保持乐观的心态，尽量避免过度焦虑或者是紧张，还需要选择正规的医疗机构。

6.牙齿矫正一定要等恒牙长齐后进行吗？	
A	牙齿矫正不一定要等恒牙完全长齐后进行，但需视具体情况而定。对于严重的咬合不正或功能性问题，儿童可在乳牙阶段接受早期矫正（一般为6-12岁），有助于改善骨骼发育，避免问题加重。恒牙完全萌出后（约12岁以上），是进行全面矫正的最佳时期，此时牙齿位置相对稳定，矫正效果更佳。建议根据牙齿发育情况，咨询专业正畸医生制定合适的矫正方案。
B	牙齿矫正不一定非要等恒牙长齐后进行。一般来说，矫正治疗的最佳时机是在孩子的恒牙开始长出并且部分长齐时，通常是12岁左右。此时，牙齿和颌骨的发育尚未完全完成，矫正效果较好。然而，某些情况下，如果存在明显的牙齿畸形或其他问题，矫正治疗可以提前开始。特别是对于部分乳牙尚未掉落的儿童，医生会根据具体情况进行判断，采取适当的治疗方式。因此，是否需要等恒牙长齐，主要依据个体的牙齿发育情况和矫正需求。
C	不一定要等恒牙完全长齐后才能进行牙齿矫正。正确的矫正时间应根据患者的具体情况来决定。一般来说，6-12岁是早期矫治的黄金时期，这时可以利用儿童颌骨发育的特点进行干预性治疗，能够预防和控制错颌畸形的发展。但如果是单纯的牙齿排列不齐，通常建议等到12-13岁恒牙基本萌出后再进行矫正。同时，成年后也可以进行牙齿矫正，只是疗程可能会相对更长。具体是否需要早期干预，建议咨询正规的口腔医院进行专业评估。
D	专家表示，有一些牙齿不齐、咬合不好的问题，可能需要等到牙齿都替换完十二三岁再做，但是对于某一些特殊的问题就不需要等到。如果等到牙齿都替完了，治疗就晚了。以下4种情况需要尽快矫治。1. 地包天。2. 下颌偏斜。3. 不良习惯导致的下颌偏斜。4. 乳牙早掉。
E	首都医科大学附属北京口腔医院医生刘博闻 提示，孩子在六七岁换牙后新长的牙齿不齐，家长不用过度焦虑，因为此时恒牙开始萌出，但颌骨发育还不完全，造成了“暂时性错颌”，最好等到恒牙都出完再考虑矫正。 若只是轻微的牙齿拥挤，随着发育，牙齿排列还会有所调整，改变孩子的不良习惯，比如咬嘴唇、咬笔头、啃手指甲等习惯，可以避免牙齿排列不齐情况加剧。
F	牙齿矫正并非一定要等恒牙长齐后进行。对于影响颌面发育的问题，如地包天等，建议早期干预。而对于单纯的牙齿不齐，可以等到恒牙替换完成后再进行正畸。因此，是否需要在恒牙长齐后进行牙齿矫正，应根据个体的具体情况和牙齿问题的性质来决定。
G	牙齿矫正是否需要等待恒牙长齐取决于具体情况。轻微错颌畸形可通过早期干预矫正，无需等恒牙长齐。严重错颌畸形则需恒牙长齐后矫正。外伤导致恒牙早失或缺失的情况，也需在恒牙长齐前采取措施。因此，是否等待恒牙长齐进行矫正应由专业医生评估后决定。
H	牙齿矫正是要在恒牙全部长出来后再进行的。牙齿矫正通常在青少年时期进行，此时为混合牙列期，恒牙逐渐替换掉乳牙。恒牙完全长出后，再开始进行牙齿矫正可以确保矫治效果。对于个别儿童需提前矫正的情况，应在专业医生评估下决定。如果存在严重的口腔疾病或全身性疾病，可能需要先治疗原发病，待病情稳定后再考虑牙齿矫正。需要注意的是，在牙齿矫正过程中，应定期复诊以调整矫正器，并保持良好的口腔卫生习惯，避免食用过硬或粘性食物，以免影响矫正效果。

7.成年人牙齿矫正和青少年有什么不同？	
A	成年人牙齿矫正与青少年有以下不同：首先，成年人的牙槽骨已经发育完成，牙齿移动速度较慢，矫正时间可能更长。其次，成年人可能面临牙周问题或缺失牙，需要综合治疗。第三，成年人的美观需求更高，通常选择隐形矫正器等低调方案。最后，成年人对疼痛和护理要求更加敏感，需要更精细的矫正方案和专业指导。因此，成年矫正需综合考虑口腔健康和个性化需求。
B	成年人和青少年在牙齿矫正上的主要区别在于骨骼的发育情况。青少年的颌骨和牙齿尚在发育阶段，因此矫正效果通常较为显著，治疗时间较短。而成年人骨骼发育完成，牙齿的移动会更慢，治疗过程可能需要更长时间。此外，成年人可能面临更多的牙齿问题，如牙周病、缺牙等，这可能需要额外的治疗。虽然矫正难度增加，但成年人依然可以通过现代矫正技术，如隐形矫正和微型牙齿矫正器，达到理想效果。
C	成年人牙齿矫正与青少年相比有以下主要区别：成年人的牙槽骨和颌骨发育已经完全，骨质较硬，牙齿移动速度较慢，矫正周期通常需要 2-3 年。而青少年正处在生长发育期，骨组织较软，牙齿移动更容易，矫正时间约 1-2 年。成年人可能伴有牙周疾病、缺牙等口腔问题，矫正前需要进行相应治疗。另外，成年人的牙齿移动过程中疼痛感较明显，对生活和工作的影响也更大，需要有更强的意志力坚持治疗。而青少年的适应能力更强，不良反应相对较轻。
D	总的来说，青少年矫正与成年人矫正的差异可以分为以下 3 点：1、治疗难易程度：成人正畸与儿童正畸相比更为复杂、成人的错颌畸形常伴随有其它口腔疾患。在开始正畸治疗前，要全面操控牙体牙周疾病，否则会影响矫治器的戴用和牙齿的移动。2、治疗时间：由于成年人生长发育已经基本完成，颌面部骨缝已变为骨性融合，可生物反应性降低，因此，牙槽骨改建比较缓慢，牙齿移动相对慢一些，须要的治疗时间较长。3、正畸方式：成人由于社会交往等因素的影响，在正畸治疗过程中对美丽度的要求较高。因此，不少成人在正畸时往往选择美丽度较高的矫治器，如：陶瓷托槽矫治器、无托槽隐形矫治器。
E	青少年的牙齿矫正通常只需要解决牙齿排列不整齐的问题，而成年人的牙齿矫正则可能涉及到更多的复杂情况，如牙齿磨损、缺失、牙周疾病等。 这些问题可能会增加矫正的难度，需要更复杂的矫正方案和更长的治疗时间。 此外，矫正方法的选择也是一个重要的区别。 对于青少年，正畸医生通常会选择传统的金属托槽矫正器，因为青少年的牙齿和骨骼在矫正过程中有较好的塑形能力。
F	成年人牙齿矫正与青少年相比，主要区别在于牙齿移动速度和治疗时间。成年人牙齿和颌骨已经发育成熟，牙齿移动速度相对较慢，因此治疗周期通常较长。另外，成年人可能存在更多口腔问题，如牙周病、牙齿磨损等，需要在矫正前先进行相应治疗。青少年矫正牙齿时，由于颌骨仍在发育，治疗效果往往更理想，治疗时间也相对较短。
G	成年人牙齿矫正和青少年相比，主要差异在于生长潜力、口腔内疾患、美观意愿及矫治力应用。青少年牙齿移动快，适应性强；而成年人矫正难度大，时间长，且常伴随其他口腔问题，对美观要求高，矫治力需轻柔。
H	1.成人牙齿矫正的前提：成人矫正牙齿与于青少年对比不一样，其矫正牙齿更为复杂。成人的错牙合畸形常伴随着别的口腔问题：如牙周病、牙齿磨耗、残冠、残根还有就是口腔颞下颌关节病症等。在进行矫正牙齿诊治前，要完整的检查诊治牙体牙周病症，拆除口腔内修复体，不然会干扰牙齿矫正器的戴用。<br>2.成人牙齿矫正的优势：成人矫正牙齿的效率和青少年比起来都是要慢一些的，不过在当代牙齿正畸诊治中，成人早就变成的正畸的一个大的受众群体。但成人牙齿矫正只要经过医生详细检查、分析，做出明确诊断，选择适当的正畸方法进行矫治，更能达到比较满意的效果。

	3.现代医疗的优势：很多朋友担心佩戴那种金属牙套难看，出席正式场合会很尴尬，现在有合适成人的牙齿矫正器，比如隐形牙齿矫正器、舌侧牙齿矫正器等，更为好看，只不过成本也较高，这时就可以选择陶瓷托槽，性价比比较高。

8.成年人牙齿矫正的利弊有什么？	
A	成年人牙齿矫正的优势包括：改善牙齿排列，提升咬合功能，增强口腔健康，同时提高个人形象和自信心。但也存在一定弊端：矫正周期较长，可能需要 1-2 年；费用较高；矫正初期可能带来不适感，如疼痛或口腔溃疡。此外，成年人骨骼生长已定型，矫正效果可能不如青少年显著，且需更严格的口腔清洁，以避免牙周病的风险。因此，矫正前需权衡利弊，并咨询专业医生。
B	成年人牙齿矫正有一定的利弊。利方面，矫正后能改善咬合关系，提升外貌自信，避免长期牙齿不齐导致的口腔健康问题（如龋齿、牙周炎）。此外，矫正有助于提高咀嚼功能和语言发音。弊方面，成年人的骨骼发育已完成，矫正时间较长，可能需要 2-3 年；矫正过程中可能出现不适或疼痛，也需要定期复诊。治疗费用相对较高，且矫正后需要长期保持，以防复发。总的来说，成年人矫正牙齿可以带来显著的健康和美观改善，但也需要考虑治疗的时间与经济成本。
C	成年人牙齿矫正的优点包括：能改善牙齿排列和咬合问题，提升面部美观，增加自信心，有助于口腔卫生的维护，减少未来出现牙周疾病的风险。同时可以改善发音问题，提高生活质量。缺点主要有：治疗周期较长，通常需要 1-2 年；费用相对较高；治疗过程中可能出现不适感和疼痛；需要定期复诊调整，对生活作息有一定影响；部分人可能出现牙齿松动或根部吸收的情况；佩戴牙套期间需要特别注意饮食习惯和口腔清洁。建议在进行牙齿矫正前，先咨询专业医生，根据个人具体情况来决定是否进行治疗。
D	一、成人牙齿矫正的优点：1. 提高口腔健康：矫正后的牙齿更容易清洁，进一步保护口腔健康。2. 改善咀嚼功能：整齐的牙齿排列能够让牙齿更好地咬合，提高咀嚼效率，使消化系统更好地运作。3. 提升自信心：整齐的牙齿能够改善面部外观，增强自信心。4. 改善口腔问题：牙齿矫正可以改善开合、下颚关节紊乱等问题，进一步改善发音和呼吸等功能。<br>二、成人牙齿矫正的弊端：1. 时间较长：相对于青少年，成年人矫正牙齿所需时间更长。这是因为成年人的颌骨已经发育完全，牙齿移动速度较慢。2. 费用较高：成人牙齿矫正的费用比青少年高出很多，主要是因为成年人矫正难度较大，所需矫治器材料和技术要求更高。3. 不适感增强：由于成年人口腔环境较为复杂，矫正过程中可能出现牙齿酸痛、口腔溃疡等不适症状。同时，成年人的自我控制能力较弱，需要更强的适应和调整能力。4. 社交影响：在矫正过程中，可能需要佩戴矫治器如牙套等，对社交产生一定影响。此外，定期复诊也会给工作和生活带来一定的不便。
E	成年人的牙齿已经发育成熟，骨密度较高，牙齿移动速度较慢，因此矫正过程可能需要较长时间。 同时，矫正过程中可能会出现牙齿松动、牙根吸收等问题，甚至可能出现牙龈肿痛、化脓等并发症。 这些都需要患者在矫正过程中做好心理准备，并严格遵医嘱进行。 其次，矫正牙齿可能对个人的生活和饮食产生一定影响。 矫正牙齿通常需要佩戴牙套或保持器等装置，这些装置可能会给日常生活带来不便，如影响进食、说话等。
F	成年人牙齿矫正的好处包括改善面部外貌、提升咀嚼功能和预防口腔疾病。弊端则涉及矫正周期较长、费用较高以及可能存在的不适感。
G	成年人牙齿矫正的利在于可改善美观、促进口腔健康、恢复咀嚼功能；弊在于可能导致牙齿松动、牙根吸收，且治疗周期长，需要耐心和配合度，还可能引发牙周组织疾病等。矫正前需全面评估，选择正规医院进行。
H	成人矫正牙能够起到恢复美观度、解除牙列拥挤、方便清洁、促进口腔健康、提高自信心等好处，但是会引起引起牙齿松动、牙齿损伤，牙龈出血，牙龈萎缩或者是矫正效果欠佳等弊端，建议到正规的医院进行检查诊断。

9.牙齿矫正会影响孩子的身高发育吗?	
A	牙齿矫正通常不会直接影响孩子的身高发育。身高主要受遗传、营养和激素水平的影响，而牙齿矫正针对的是牙齿排列和咬合问题。然而，如果孩子咀嚼功能因牙齿问题受限，可能间接影响营养吸收，从而对生长发育产生一定影响。选择合适的矫正方法并确保均衡饮食，可以最大程度减少潜在影响。牙齿矫正过程需由专业口腔医生评估，家长也应定期带孩子检查，关注整体健康。矫正期间若发现孩子生长发育异常，应及时咨询儿科医生或内分泌医生，以确保问题得到妥善处理。
B	牙齿矫正通常不会直接影响孩子的身高发育。身高的增长主要与骨骼生长、营养、遗传等因素有关，而牙齿矫正主要集中在牙齿排列和咬合的改善。矫正过程中，可能需要拔牙或使用扩展器，但这些操作不会影响骨骼的生长板或整体身高发育。通常来说，牙齿矫正对儿童和青少年的身高发育不会产生不良影响。但如果有特殊的骨骼问题，如颌面畸形，可能需要进一步的医学评估。总的来说，牙齿矫正是安全的，不会影响身高发育。
C	牙齿矫正本身不会直接影响孩子的身高发育。但值得注意以下几点:<br>首先，矫正牙齿的最佳时期恰好是青春期生长发育阶段，此时孩子正处于快速生长期。牙齿矫正可能会引起短期的不适感，影响进食，如果因此导致营养摄入不足，可能会间接影响身高发育。解决方案是:在矫正期间注意调整饮食结构，选择富含蛋白质、钙质等营养的软食，保证充足的营养摄入。同时保持规律作息，确保充足睡眠，这样就不会对身高发育造成影响。总的来说，只要注意饮食营养均衡，规律作息，牙齿矫正是不会影响身高发育的。
D	矫正是牙槽骨改建的过程，治疗结果会与上下颌骨的生长发育有关，但矫正本身不会影响儿童身高的生长。
E	目前的研究表明，牙齿矫正治疗不会对孩子的身高发育产生直接影响。牙齿矫正主要是通过调整牙齿和颌骨的位置来改善咬合功能和美观度，与孩子的骨骼生长过程并没有直接关联。牙齿矫正治疗主要适用于儿童和青少年时期，这个阶段骨骼和牙齿正处于发育和生长阶段。牙齿矫正通过施加轻微的力量来改变牙齿和颌骨的位置，以使它们更加对称和协调。这些力量是适度的，不会对整体的骨骼生长造成重大影响。然而，需要注意的是，牙齿矫正可能会对孩子的咬合和口腔功能产生积极的影响，从而带来身体其他方面的改变。例如，更好的咬合功能可以改善食物咀嚼和消化，有助于营养摄入和身体健康。此外，矫正后的美观牙齿也可能会增强孩子的自信心和社交能力，进而对整体的身心发育产生积极的促进作用。值得一提的是，在进行牙齿矫正治疗时，孩子的整体身体健康和营养摄入也是重要的因素。确保孩子摄入充足的营养物质，维持健康的生活方式，对于正常的身体发育至关重要。<br>总的来说，牙齿矫正治疗不会直接影响孩子的身高发育。这项治疗主要关注改善口腔健康和美观度，对整体的身体发育并没有负面影响。然而，保持良好的营养摄入和健康的生活方式对孩子的整体发育仍然非常重要。如果您有任何关于牙齿矫正治疗的疑虑或问题，最好咨询专业的牙医和矫正专家。
F	牙齿矫正不会影响孩子的身高发育。牙齿矫正主要通过外力调整牙齿位置，与全身生长激素、骨骼发育无直接关联。身高发育受遗传、营养、激素等多种因素影响，而矫正器施加的力仅作用于牙齿和牙槽骨，对生长激素的分泌和全身骨骼发育没有影响。因此，家长不必过于担心牙齿矫正会影响孩子的身高发育。
G	牙齿矫正通常不会影响孩子的身高发育。牙齿矫正是通过矫正器对牙齿施加轻微力量，逐渐移动牙齿以改善排列和咬合，不会对骨骼和身体其他部位产生影响。身高发育主要受遗传、营养、运动等因素影响，与牙齿矫正无直接关联。
H	孩子的身高发育主要受遗传、营养、运动等因素的影响，牙齿矫正通常不会影响孩子

	的身高发育。牙齿矫正是通过矫正器对牙齿施加轻微的力量，逐渐移动牙齿，达到矫正牙齿排列不齐、咬合不正等问题的目的。矫正器的力量非常轻微，不会对骨骼和身体其他部位产生影响。有些家长担心牙齿矫正会影响孩子的咀嚼功能，从而影响营养吸收和生长发育。实际上，牙齿矫正并不会影响孩子的咀嚼功能，相反，通过矫正可以改善牙齿的咀嚼功能，促进营养吸收，有助于孩子的生长发育。当然，如果孩子在矫正期间出现了口腔问题，如牙龈炎、龋齿等，需要及时治疗，以免影响孩子的口腔健康和生长发育。总之，牙齿矫正不会影响孩子的身高发育，家长不必过于担心。如果孩子需要进行牙齿矫正，建议选择正规的口腔医院或诊所，由专业的医生进行诊断和治疗。

10.牙齿矫正的好处是什么？	
A	牙齿矫正不仅可以改善牙齿排列和咬合问题，还能显著提升口腔健康。整齐的牙齿更易清洁，有助于预防龋齿、牙龈炎等口腔疾病。此外，矫正后的牙齿能改善面部美观，增强自信心，对个人形象有积极影响。功能上，正确的咬合关系提高咀嚼效率，促进消化吸收，对整体健康有益。牙齿矫正也能纠正发音问题，让言语表达更清晰。综合来看，牙齿矫正带来的不仅是外在美，更是内在健康与自信的全面提升。
B	牙齿矫正的好处主要包括改善口腔健康、美化外观和增强自信心。通过矫正，错牙齿可以减少龋齿、牙周病和咬合不正带来的牙齿磨损问题，提升咀嚼和发音功能。整齐的牙齿有助于口腔清洁，降低食物残留，减少细菌滋生。此外，矫正后牙齿排列更加美观，有助于改善个人形象，提升自信心，尤其在社交场合中更加自如。总体而言，牙齿矫正不仅仅是为了美观，也有助于长期的口腔健康。
C	牙齿矫正具有多方面的积极意义:从美观角度来看,整齐的牙齿能让面部轮廓更加协调,提升个人形象和自信心。从功能角度而言,正确的咬合能够改善发音问题,使咀嚼更有效率,预防牙周疾病。此外,良好的牙齿排列可以减少食物残渣堆积的机会,降低蛀牙和口腔炎症的风险。矫正还能缓解颞下颌关节疾病带来的不适,预防面部肌肉疲劳和头痛等症状。从长远来看,及早进行牙齿矫正有助于维持口腔健康,减少未来可能出现的并发症。不过矫正过程需要保持耐心,认真做好口腔卫生。
D	为什么牙齿矫正很重要？ 牙齿和颌骨在错误的位置上生长发育，是正确口腔卫生护理的一大障碍。 因此，牙齿矫正的目的不仅仅是为了获得完美的牙齿，而是更进一步。对于各个年龄段的人来说，这是一种越来越常见的治疗方法，因为它可以解决由于牙齿拥挤或颌骨移位等原因造成的咀嚼功能障碍等并发症。
E	这个问题其实要根据每个病人自己的要求。很多病人来矫正，肯定是带着目的来的，每个人有不同的目的，比如最常见的一个目的是龅牙，很多病人来了以后是觉得自己龅牙、嘴巴突、没有下巴等情况，对于他的诉求就是美观，为了矫正他的美观要求，他愿意接受长达 2-3 年的矫正时间，从这个角度来说，对他肯定是有利的。还有些病人，是为了健康因素来的，比如有牙周炎的，在牙周炎治疗以后，可能会要求做牙齿的角度调整、牙齿排列调整，更有利于牙周的维护。这种情况也是需要正畸科来进行矫正的。还有些比如因为牙周原因，有些牙齿不整齐也会影响到牙周的维护、口腔卫生的清洁，这种情况，病人从美观的要求上，觉得牙齿不齐、不好看。另外从健康的角度、从口腔卫生维护的角度等，这种情况也都是需要矫正的。所以如果病人有需求，在正规的正畸医生的操作之下，矫正肯定是对他有利的。
F	牙齿矫正可以改善咬合，提升口腔功能，预防牙齿磨损和牙周病。同时，整齐的牙齿更易清洁，有助于维护口腔卫生。美观的牙齿也能增强自信，改善社交和职业形象。
G	牙齿矫正可以带来多方面的好处。它能改善牙齿排列，使口腔更健康，减少牙齿拥挤导致的清洁难题和蛀牙风险。同时，整齐的牙齿能提升个人形象，增强自信。此外，牙齿矫正还有助于改善咀嚼功能，减轻牙齿磨损和关节压力，对长期口腔健康及面部美观都有积极影响。
H	整牙的好处在于可以改善外观、矫正咬合关系不正、保护口腔卫生。<br>1、如果牙齿存在排列不整齐或牙缝过大的情况，往往会对外观造成一定影响，甚至有可能会导致面部畸形，通过整牙也就是牙齿矫正，能够让牙齿排列整齐，让牙齿更加美观，患者也可以恢复自信。2、矫正咬合关系不正：如果牙齿排列不整齐，往往会导致咬合关系不正，从而影响到咀嚼的功能，整牙之后会让牙齿恢复整齐，咬合也可以恢复到良好的状态。3、保护口腔卫生：当牙齿排列不整齐时，不利于清洁口腔，整牙之后牙齿排列整齐，更有利于清洁牙齿，保护口腔卫生。如果存在牙齿排列不整齐，一定要及时到医院口腔科就诊，由医生检查诊断后制定合适的诊疗方案。

11.牙齿矫正仅仅是为了美观吗?	
A	牙齿矫正不仅仅是为了美观，还具有重要的健康意义。首先，它能改善咬合关系，帮助更有效地咀嚼食物，促进消化系统健康。其次，整齐的牙齿更易清洁，降低蛀牙和牙周病风险。此外，矫正能纠正因牙齿排列异常引发的颞下颌关节问题，缓解因不良咬合导致的头痛或面部疼痛。在心理层面，整齐的牙齿提高个人自信心和社交能力。因此，牙齿矫正不仅关乎美观，还涉及口腔功能与整体健康。
B	牙齿矫正不仅仅是为了美观，还具有重要的功能性意义。通过矫正牙齿，可以改善咬合关系，减少因牙齿排列不整齐而导致的咀嚼困难和消化问题。同时，正确的咬合有助于减少牙齿磨损，降低口腔健康风险，如牙龈炎和牙齿龋坏等。牙齿矫正还可以缓解因牙齿不齐引起的面部不对称，提升面部美观。综合来看，牙齿矫正是改善口腔健康和提升个人自信的重要手段。
C	牙齿矫正不仅仅是为了美观。从功能角度来看,牙齿不齐可能导致咀嚼功能障碍,影响进食效率和消化功能。不整齐的牙齿还容易藏污纳垢,增加龋齿和牙周疾病的风险。某些牙齿排列问题还会影响发音清晰度,给日常交流带来不便。从长远健康角度看,错牙合可能引起颞下颌关节疾病,造成头痛、耳鸣等不适。因此,牙齿矫正不仅能改善外观,更重要的是能预防口腔疾病,维护口腔和全身健康。
D	牙齿矫正是常见的口腔护理，拥有 11 年牙医经验的 Zenyum 牙科副总裁刘于萍接受《联合早报》访问时说：“矫正牙齿不仅是为了美观，还有很多好处，可以改善口腔健康，因为可以用牙线，也能刷到口腔中难以触及的部位。长远来看，有助于保持牙龈健康。”
E	误区一：正畸只是为了牙齿变得漂亮.有些前来就诊的成年人（尤其是男士）常会感到不好意思，甚至因为害怕被周围的同事或朋友笑话而无法下定决心治疗。 会有这种想法，可以理解但并不可取。 正畸治疗除了美化笑容外，其作用还有很多，如：有利于牙周健康的维护，不整齐的牙齿自然难于清洁，易发牙石沉积，牙龈红肿增生（会有刷牙出血、口臭等），时间长了可能发生牙周病变（牙齿松动，牙龈退缩等），就更加难于治疗！
F	牙齿矫正不仅仅是为了美观，它还有助于改善口腔功能和健康。矫正可以解决咬合问题，减少牙齿磨损，预防牙周病，改善发音，提高咀嚼效率，同时也能增强自信心。
G	牙齿矫正并不仅仅是为了美观。虽然整齐的牙齿能提升个人形象，但更重要的是，矫正可以解决牙齿排列不齐、咬合不良等问题，有助于维护口腔健康，预防牙齿疾病和龋齿，减轻牙齿磨损和牙周压力。同时，良好的咬合还能提高咀嚼效率，促进消化，对整体健康产生积极影响。
H	牙齿矫正不仅仅是为了美观，还具有多方面的健康和功能上的益处。牙齿矫正可以改善口腔健康、增强口腔功能、预防口腔疾病，并提升个人的自信心。<br>首先，牙齿矫正有助于改善口腔健康。不整齐的牙齿难以清洁，容易滋生细菌和食物残渣，导致龋齿、牙周炎和口臭等问题。通过矫正，牙齿排列整齐，更容易清洁，减少了菌斑和龋齿的风险。此外，矫正还能调整咬合关系，减少咬合不紧或深覆合等问题，预防颞下颌关节紊乱等疾病。其次，牙齿矫正对口腔功能有显著影响。不整齐的牙齿会影响咀嚼、发音和吞咽功能，导致消化不良和营养吸收不良。通过矫正，可以提高咀嚼效率，改善发音清晰度，使食物更容易被消化吸收。整齐的牙齿还能增强自信心，尤其是对于那些因为牙齿问题而感到自卑的人来说，矫正可以显著提升他们的外貌美观和心理健康。最后，牙齿矫正的方法多种多样，包括佩戴传统金属牙套、陶瓷牙套和隐形矫正器等。每种法都有其优缺点，选择合适的矫正方法应根据个人需求和经济能力来决定。

12.牙齿矫正的风险有哪些？	
A	牙齿矫正虽能改善牙齿排列和咬合功能，但也存在一定风险。首先是牙齿和牙根吸收，可能导致牙根缩短。其次，矫正期间口腔卫生难以维持，易引发龋齿或牙龈炎。此外，部分患者可能出现矫正后牙齿反弹或复发现象。矫正器还可能引起口腔溃疡或不适感。少数患者对矫正材料过敏，导致过敏反应。长时间矫正可能对咀嚼功能短期内造成影响。因此，矫正需在专业医生指导下进行，密切监控治疗进程以降低风险。
B	牙齿矫正虽然可以改善咬合和外观，但也存在一些风险。首先，牙齿矫正可能引起口腔不适，如疼痛和牙龈炎症。其次，矫正过程中可能出现牙根吸收、牙齿松动或牙齿不均匀的情况。另一个风险是矫正器可能对口腔内软组织造成磨损或损伤，特别是没有定期检查时。长期佩戴矫正器还可能影响口腔卫生，增加蛀牙和牙龈疾病的风险。因此，在矫正过程中，需定期复诊并保持良好的口腔卫生习惯，以确保治疗顺利进行。
C	矫正过程中可能出现牙齿酸痛和不适，这是正常现象。但也存在一些潜在风险:牙根吸收会导致牙齿松动,牙齿过度移动可能损伤牙周组织。矫正器可能刺激口腔黏膜,引起溃疡。如果卫生习惯不好,容易形成牙菌斑和蛀牙。个别病例会出现颞下颌关节紊乱。极少数情况下会出现过敏反应。此外,如果方案设计不当或操作不规范,可能影响咬合和面型。建议选择正规医院和具备资质的医生进行治疗。
D	传统的正畸技术无法根治造成牙齿不齐的根本原因，同时还存在其他一些风险或局限性，包括手术、牙釉质和牙根损伤以及高复发可能，除非长期佩戴固定或活动保持器。
E	1、颞下颌关节紊乱。主要临床表现为关节区疼痛、运动时关节弹响、下颌运动障碍等。疼痛部位可在关节区或关节周围；并可伴有轻重不等的压痛。关节酸胀或疼痛尤以咀嚼及张口时明显。弹响在张口活动时出现。响声可发生在下颌运动的不同阶段，可为清脆的单响声或碎裂的连响声。常见的运动阻碍为张口受限，张口时下颌偏斜，下颌左右侧运动受限等。此外，还可伴有颞部疼痛、头晕、耳鸣等症状。2、骨开裂。缺损的牙槽骨和黏膜组织范围到达牙槽嵴顶部，就像是开了一扇“门”，这扇门打开之后，随之而来的就是牙周组织被严重破坏，继而牙槽骨萎缩，牙龈萎缩。形成骨开裂。3、骨开窗。因为牙槽骨局部缺损，导致部分牙根在牙槽骨甚至牙龈上暴露出来，就像是开了一扇“窗”，然后就会引起牙痛，严重的可能会引发牙髓坏死。4、牙根吸收。从 CT 上看起来，牙齿的根部出现缩小、萎缩等情况。
F	牙齿矫正的风险主要包括：颞下颌关节紊乱、骨开窗、骨开裂、牙根吸收等。此外，还可能对牙槽骨高度产生影响，导致牙槽骨降低；牙髓在矫正初期可能会有暂时性和轻微的炎症反应；牙齿在矫正过程中可能会出现松动；以及牙釉质脱矿及龋病、牙周组织损坏等。这些风险在专业医生的指导下是可控的，因此选择正规医疗机构和经验丰富的医生是非常重要的。
G	牙齿矫正的风险包括牙齿疼痛与不适、牙龈炎与牙周疾病、牙齿松动、牙根吸收、牙釉质脱矿与磨损、软组织损伤、面部肌肉改变（如牙套脸）及心理压力等。选择正规医院和专业医生，遵循医嘱并保持良好的口腔卫生习惯，可降低这些风险。
H	1、牙齿疼痛与不适：在矫正初期，由于矫治器对牙齿施加力量，可能会引起牙齿疼痛或不适感，这种疼痛通常会在一段时间后逐渐减轻。2、牙龈炎与牙周疾病：矫正过程中，由于矫治器的存在，口腔卫生可能难以维护，食物残渣和细菌容易在口腔内滋生，从而增加患牙龈炎、牙周炎等牙周疾病的风险。3、牙齿松动：矫治器对牙齿施加的力量可能导致牙齿松动，特别是在矫正力度过大或调整过于频繁的情况下。牙齿松动可能会影响正常咀嚼功能，如果松动症状严重，则需及时就医。4、牙根吸收：牙根吸收是牙齿矫正过程中可能出现的并发症之一，通常与矫治力过大或牙齿移动过快有关。牙根吸收可能导致牙齿变得脆弱，甚至增加牙齿脱落的风险。5、牙釉质脱矿与磨损：矫治器的使用可能会增加牙釉质脱矿的风险，使牙齿变得脆弱易碎。同时，矫治器还可能导致牙齿表面出现磨损或划痕。6、软组织损伤：在矫正过程中，如果矫治器部件如钢丝、托槽等变形或脱落，可能会对舌头、嘴唇等软组织造成损伤

13.牙齿矫正中常见的误区有什么？	
A	牙齿矫正中的常见误区包括：①**矫正只适合青少年**：实际上成年人也可以矫正，矫正效果与年龄无绝对关系；②**牙齿矫正会导致松动或脱落**：正规操作不会损伤牙齿根基，松动是生理性调整；③**隐形矫正效果不如传统矫正**：隐形矫正同样能达到理想效果，具体选择需视情况而定；④**矫正后效果永久**：若不坚持佩戴保持器，牙齿可能反弹；⑤**牙齿矫正费用昂贵且没必要**：矫正不仅改善美观，还能优化咬合和口腔健康，是长远投资。
B	牙齿矫正中常见的误区包括：1. **矫正只为美观**：很多人误认为牙齿矫正仅是为了外观，忽略了其改善咬合、预防牙齿疾病的作用。2. **矫正年龄有限**：认为只有青少年适合矫正，事实上成年人也可以进行矫正，只要口腔健康。3. **佩戴时间越短越好**：有些人追求快速结果，忽视了矫正过程的耐心与专业性，可能导致效果不持久。4. **矫正后不需要护理**：矫正结束后，部分人忽略保持器的使用，导致牙齿反弹。<br>正确的矫正应根据专业建议，注重长远效果。
C	关于牙齿矫正，有以下常见误区需要澄清:年龄限制方面，很多人认为只有青少年才能矫正，但实际上成年人同样可以进行牙齿矫正。在治疗时间上，不是越快越好，正常需要 1-2 年，过快的矫正反而可能带来并发症。还有人觉得戴牙套后就不能吃任何硬物，其实只要注意避免过硬、过粘的食物即可。有些人担心矫正会损伤牙齿，但只要选择正规医院和合格医生，遵医嘱进行治疗是安全的。不要盲目相信价格越高效果越好，合适的方案比价格更重要。
D	一：年龄大了不能矫正。二：牙齿矫正后会松动。三：牙齿矫正老了会掉牙。四：龅牙或牙不齐，寻求七天快速矫正。五：矫正只是为了美观。六：矫正一定要拔牙。七：拔牙矫治后会留有牙缝。八：牙齿没换完 不能开始矫正。九：牙齿矫正一定很疼。十：矫正只能戴牙套，影响美观。十一：矫治力越大，牙齿移动越快；矫治力小，不疼，牙齿移动慢或不移动。十二：矫正期间只要缩短复诊间隔，就诊频繁，就能加快进度继而缩短疗程。十三：严重的牙周炎 不能矫正。十四：戴牙套会腐蚀牙面。十五：牙齿矫正只能解决牙齿问题。十六：牙齿矫正可以瘦脸或者改变脸型。十七：牙齿矫正会影响孩子的身高发育。误区十八：成年人牙齿矫正容易反弹。十八：成年人牙齿矫正容易反弹。十九：摘下牙套后，正畸治疗就结束了吗？二十：很多患者喜欢和别人比较治疗方案、疼痛感受、牙齿移动快慢、疗程甚至费用等。
E	误区一：年龄大了不能矫正 牙齿移动是生命体内都会存在的骨改建过程，因此牙齿矫正老少皆宜。 但不同个体存在的问题不一样，开始矫正的时机也不尽相同，是否可以矫正，应从专业的正畸医师处寻求答案。 误区二：牙齿矫正后会松动 牙齿本身也不会一直待在原地不动，而是处于稳定与移动的平衡中。 矫正是通过外力暂时打破这个平衡，为牙齿“搬家”。
F	牙齿矫正误区包括：认为矫正只适合儿童；矫正会导致牙齿松动；矫正后效果是永久的；矫正过程无痛；拔牙矫正会影响健康。实际上，矫正适合任何年龄，成人也可矫正；牙齿松动是因牙周病；矫正效果需长期保持；矫正初期会有不适；拔牙矫正是为腾出空间，不会影响健康。
G	牙齿矫正中常见的误区包括：认为年龄大了不能矫正、矫正后牙齿会松动或老了会掉牙、寻求快速矫正方法、矫正只是为了美观、矫正一定要拔牙、拔牙矫治后会留有牙缝、成年人牙齿矫正容易反弹等。
H	牙齿矫正的五个误区分别是以后牙齿容易脱落、拔牙有害、矫正牙齿特别疼痛等。牙齿矫正属于比较常见的治疗方法，对于各种因素引起的牙齿排列不整齐或者是缝隙增大都有很好的作用，在矫正过程中不会导致牙齿松动以及牙齿损伤，而且不会出现特别疼痛。做牙齿矫正之前最好到医院口腔科做检查，可以根据牙齿畸形原因和严重程度针对性治疗。在这个期间还要改善饮食，最好不要吃太硬的食物，以免出现矫正受到影响。

14.如果需要进行牙齿矫正，治疗不及时会有哪些影响？	
A	牙齿矫正治疗不及时可能导致多种不良影响。首先，不整齐的牙齿会加重咬合不正，影响咀嚼功能，甚至引发颞下颌关节紊乱。其次，不良的牙齿排列容易导致清洁困难，增加龋齿和牙周病的风险。此外，错位的牙齿可能影响面部美观，进而对患者的自信心和心理健康造成负面影响。儿童时期若错过生长发育黄金期，矫正难度会增加，治疗周期更长，效果可能受限。因此，应及早咨询专业正畸医生并采取治疗。<br>治疗不及时可能导致以下影响：<br>1. **牙齿健康问题**：龋齿、牙龈炎风险增加。<br>2. **咬合问题**：咀嚼困难，可能引发颞下颌关节紊乱。<br>3. **面部发育**：影响面部对称和美观。<br>4. **心理影响**：因牙齿不齐产生自卑，影响社交。<br>5. **治疗难度增加**：后续矫正更复杂，费用更高。<br><br>建议尽早咨询正畸医生。
B	如果牙齿矫正治疗不及时，可能会导致一系列口腔问题。首先，牙齿错位可能加重，影响咬合功能，导致咀嚼困难。长时间的咬合不正还可能引发颞颚关节（TMJ）问题，造成头痛、面部疼痛等症状。其次，错位的牙齿更容易积累食物残渣和细菌，增加龋齿和牙周病的风险。此外，牙齿不齐还可能影响外观和自信心，给心理带来负担。因此，及时矫正牙齿有助于改善口腔健康和整体生活质量。
C	牙齿矫正若不及时进行治疗，可能会带来以下影响：首先，错牙合畸形会逐渐加重，导致咬合问题更难纠正，增加后期治疗难度和时间。其次，不整齐的牙齿容易藏污纳垢，增加龋齿和牙周疾病的风险。再者，错位的牙齿可能造成咀嚼功能障碍，影响消化和营养吸收。从美观角度来看，牙齿不齐会影响面部轮廓和笑容美感，可能给人带来心理压力。此外，某些严重的错牙合还可能引起颞下颌关节紊乱，造成头痛、耳鸣等不适症状。
D	牙齿不整齐造成咬合关系紊乱，咀嚼功能下降，可出现面条咬不断、肉嚼不碎的现象，增加肠胃负担，直接影响消化系统，进而影响全身健康。 二是影响美观。 牙齿拥挤、前突、“地包天”等，可能会影响脸型，笑起来非常难看，引发个体自卑的心理情绪，产生容貌焦虑，严重影响社交。
E	如果错过了最佳矫治时间，比如很多成年人选择进行矫治也是一个不错的时间。只是随着年龄推移，新陈代谢、颌骨牙齿改建能力和适应能力都会逐渐下降，矫治效果、稳定性会下降，牙龈萎缩等不良并发症的发生率可能相对更高，付出代价可能更多。牙列不齐、矫正不及时一般不会影响生命安全，牙列矫治最佳时机一般在青少年时期，因为此时进行矫正，新陈代谢和生理改建最活跃，尽早破除这种功能干扰的情况下，建立一个和谐、美观的咬合状况，对将来生活质量也有很大帮助。
F	牙齿矫正若不及时，可能会影响口腔及颜面部发育，导致牙齿不齐不利于口腔卫生健康，增加龋坏、牙石、牙龈炎和牙周炎等口腔疾病的风险。同时，还可能影响口腔功能，如咬合功能紊乱、发音不清，以及影响美观和社交，对心理发育造成不良影响。
G	如果牙齿矫正治疗不及时，可能会导致牙齿畸形、影响美观，还可能导致咬合不良、咀嚼功能下降，甚至引发牙周疾病，如牙龈炎、牙周炎等，出现牙龈出血、牙齿松动等症状。此外，还可能影响颞下颌关节功能，导致关节疼痛、弹响等问题。
H	如牙齿不及时进行矫正，可能会导致牙齿炎症、咬合创伤、以及影响脸型等<br>1、牙齿炎症：如果牙齿不整齐，可能在刷牙时无法彻底地清洁，容易导致食物嵌塞在牙缝里。时间长了可能会引起菌斑和牙结石，通常表现为口臭、牙周病、龋病等牙齿炎症。2、咬合创伤：如上下颌骨前突或后缩或牙列不齐，导致咬合关系不正常，或咬

	合力量不协调，会引起的咀嚼肌系统的损伤，出现咬合创伤、牙齿松动或者牙根囊肿等情况。3、影响脸型：牙齿畸形还可能影响脸型，如引起脸歪、颞下颌关节紊乱综合症等，如果长期偏侧咀嚼、一侧咬合不适等，容易引起张口弹响、疼痛，或者张不开嘴、脸歪等情况。通常有开颌、偏颌或者地包天的情况时，咀嚼效率较低，导致食物在口腔内难以磨碎，吞咽时可能会增加胃肠道的负担。如果出现牙齿排列不齐、上下颌的牙弓间的咬合关系异常、颌骨的形态、大小和位置异常应及时就医。牙齿矫正治疗是通过口腔正畸专科检查后，对错颌的程度进行分析，明确治疗目标，制定个性化的矫治方案，选择合理的矫治装置进行治疗。

15.牙齿矫正后会复发吗？	
A	牙齿矫正后可能会出现复发的情况，这主要与个体差异、治疗方法和后续维护有关。矫正过程中牙齿受到的移动力量需要时间稳定，而牙周组织的重建较为缓慢，如果未坚持佩戴保持器，牙齿可能逐渐恢复至原始位置。此外，咬合力、口腔习惯（如咬唇、咬笔）以及牙齿生理性移动也可能导致复发。为了尽量减少复发风险，患者应严格遵循医生建议，定期复诊，长期坚持佩戴保持器，并保持良好的口腔卫生习惯和健康的生活方式。
B	牙齿矫正后是否复发主要取决于矫正后的维护情况。矫正治疗完成后，牙齿仍然可能受到“回弹效应”，即逐渐向原来的位置移动。为避免复发，患者需要佩戴保持器，通常需要在较长时间内坚持使用，以帮助牙齿稳定在新的位置。此外，保持器的佩戴时间、口腔习惯和口腔健康状况也会影响矫正效果。复发的风险较高的人群，通常需要长期关注和定期复查，以确保矫正效果持久。因此，积极的后期维护非常关键。
C	牙齿矫正后确实存在复发的可能性。主要原因包括:第一，牙齿有"记忆"，会有回到原位置的自然倾向；第二，如果没有按医嘱佩戴保持器，更容易导致复发；第三，年龄增长带来的生理变化也会影响牙齿位置。预防复发的关键是:严格遵医嘱佩戴保持器，保持良好的口腔卫生习惯，定期复查。即使做好这些，仍有小概率出现轻微复发，但通常不会影响整体效果。若发现复发迹象，应及时就医检查。
D	是的，隐适美矫正治疗后牙齿会复发，就像传统牙套矫正后牙齿会复发一样。 复发的风险与所使用的正畸治疗类型无关，而是牙齿想要移动回原来位置的自然趋势。 这就是为什么在隐形矫正治疗后按照医嘱佩戴保持器与佩戴牙套一样重要的原因。
E	牙齿矫正完之后，牙齿必然会复发，只是时间和程度问题而已。没有一个人的牙齿矫正完会一直稳定在刚刚矫正后的位置上，人的牙齿一生都是在进行微小的移动的。每个人做完牙齿矫正之后，医生都会建议患者去戴保持器，帮助牙齿能更长时间地稳定在矫正后的位置上，但即便是戴保持器，也无法保证牙齿一生不变。所以牙齿在后半生通过不断的微小的移动，再次出现不齐甚至牙间隙的情况很常见。如果轻度出现，可以不管，如果比较严重，出现比较大的牙间隙，甚至比较严重的牙列不齐，可以通过二次矫正去治疗。戴保持器是很有效的防止牙齿复发的一种方法，因此建议患者在刚结束第一年最好全天都能戴保持器，即便是结束多年以后，也建议患者每周挑两个晚上，偶尔戴一下保持器，这样能很大程度上的减少复发的程度，延缓复发的时间，但是也没有绝对不复发的这种患者存在。
F	牙齿矫正后复发是可能的，因为牙齿终生都在移动，不可能完全保持矫正结束时的状态。轻微的移动并不代表复发，例如从 100 分保持到 95 分就很不错了。矫正后的复发是绝对的，只是每个人的复发情况不同。因此，为了减少复发的可能性，患者需要在矫正后严格佩戴保持器，并遵循医生的指导。
G	牙齿矫正后有可能复发。复发的原因可能包括矫正效果不佳、牙齿移动速度过快、未正确佩戴保持器、存在口腔不良习惯以及未定期复查等。因此，牙齿矫正后需严格遵医嘱佩戴保持器，并改正不良习惯，定期复查，以降低复发风险。
H	牙齿矫正后可能会反弹，特别是在去除矫正器后的前几年内，如果不按照医嘱佩戴保持器或者不遵循正确的口腔卫生习惯。1、会反弹。牙齿矫正后会反弹的原因主要是因为牙齿和牙周组织需要时间来适应新的位置。在矫正治疗期间，牙齿通过外力移动到理想的位置，但牙齿周围的骨骼和组织需要一段时间来重建和稳定。如果在这个关键期没有正确使用保持器，牙齿很可能会回到原来的位置或产生新的错位。2、不会反弹。如果患者在矫正治疗后严格遵守医嘱，正确佩戴保持器，并保持良好的口腔卫生习惯，牙齿反弹的可能性会大大降低。保持器的作用是帮助牙齿和周围组织适应新的位置，防止牙齿移动。因此，正确使用保持器是防止牙齿反弹的关键。总之，牙齿矫正后是否会反弹取决于患者矫正后的护理和保持器的使用情况。遵循医生的指导和建议，可以有效减少牙齿反弹的风险。

16.牙齿矫正后如何避免复发？	
A	牙齿矫正后避免复发需要注重以下几点：1. **佩戴保持器**：遵从医嘱佩戴保持器，一般需持续 1-2 年，部分病例可能更长。2. **定期复诊**：按时复诊，医生可根据牙齿移动情况调整方案。3. **保持良好习惯**：避免咬硬物、不良口腔习惯（如咬指甲、咬笔头等）。4. **维护口腔卫生**：定期刷牙、使用牙线，预防牙周炎等可能导致牙齿移动的疾病。5. **饮食注意**：避免过多摄入硬、黏性食物，减少对牙齿的压力。坚持上述措施，可有效降低牙齿复发风险。
B	牙齿矫正后避免复发的关键是保持长期的正畸护理。首先，佩戴保持器是必不可少的，通常需要在矫正结束后持续佩戴保持器一段时间，并根据医生建议逐渐减少佩戴时间。其次，定期复诊非常重要，确保牙齿的稳定性，并及时调整保持器的使用。第三，避免不良习惯，如咬硬物、用牙齿开瓶盖等，以免对牙齿造成不必要的压力。此外，保持口腔卫生，定期刷牙和使用牙线，减少牙龈炎和蛀牙的风险，确保牙齿健康。
C	为避免牙齿矫正后复发，需要注意以下几个关键方面：首先，必须严格按医嘱佩戴保持器，初期全天候佩戴，后期遵医嘱逐步调整佩戴时间。其次，保持良好的口腔卫生习惯，每天认真刷牙和清洁保持器。再者，改掉不良口腔习惯，如咬笔、单侧咀嚼等。此外，定期复查很重要，及时发现和解决问题。最后，保持健康饮食习惯，避免过硬食物对牙齿造成额外压力。
D	坚持佩戴保持器：牙齿矫正完成后，佩戴保持器是防止复发的关键。保持器需要按照医生的指导佩戴，一般建议全天佩戴，特别是初期，仅在进食和清洁时取下。随着时间的推移，佩戴时间可以逐渐缩短，但通常建议至少佩戴两年以上，甚至更长时间，以稳固矫正结果。<br>破除口腔不良习惯：咬唇、吐舌等口腔不良习惯是导致复发的重要原因之一。在矫正期间及矫正后，必须积极破除这些不良习惯，可以通过行为干预、心理治疗等方式来辅助。<br>定期复诊与复查：定期复诊是确保矫正结果稳定的重要手段。复诊时，医生可以对牙齿和矫正器进行检查和调整，及时发现并解决问题。同时，患者也应定期进行洗牙，保持口腔健康，防止牙菌斑和牙结石的形成。<br>注意饮食习惯：饮食习惯对牙齿矫正结果的影响也不容忽视。矫正后，应避免食用太硬、过粘的食物，以减少对牙齿和矫正器的损伤。同时，合理调整饮食结构，多摄入富含维生素和矿物质的食物，有助于牙齿健康。<br>科学刷牙与清洁：科学的刷牙和清洁是保持口腔卫生的基础。矫正后，应选择适合矫正后牙齿的软毛牙刷，细心刷洗每一颗牙齿和矫正器。每天至少刷牙两次，每次刷牙持续 2-3 分钟。此外，使用牙线和牙间刷来清洁牙齿之间的空隙，防止食物残渣滞留，减少龋齿和牙龈问题的发生。
E	1、严格按照医嘱佩戴保持器，记得不要“偷工减料”哦，这是防复发的关键。 2、必须戒除吐舌、咬唇等不良口腔习惯，也可以较好的预防牙齿矫正复发、反弹。 3、选择专业的口腔医院以及经验丰富的牙科医生才是矫正效果的最好保障。 4、持之以恒，定时复诊，高度配合医生的诊疗方案。 5、合理饮食，细嚼慢咽，不可进食过硬、过黏的食物。 6、注意口腔清洁卫生，减少牙龈、牙周等口腔疾病的发生。
F	牙齿矫正后避免复发，关键是严格佩戴保持器，初期全天佩戴，之后逐渐减少时间。同时，保持良好的口腔卫生习惯，定期复查，避免不良口腔习惯如咬唇、吐舌等。
G	牙齿矫正后为避免复发，需注意以下几点：破除口腔不良习惯，如咬唇、吐舌等；正确佩戴保持器，佩戴时间需遵医嘱；保持口腔卫生，避免吃过硬食物；定期复查，关注矫正效果；必要时采取过度矫治或手术治疗。
H	预防牙齿矫正后反弹需要使用保持器，坚持佩戴两年以上。牙齿矫正通常能够改善牙

	齿畸形，一般可以使用牙套进行矫正，但是在矫正以后牙齿也有可能会出现反弹的情况，因此矫正完成以后，需要使用保持器，可以预防牙齿反弹，但是需要坚持才能够有效果，一般需要两年左右，效果会比较好。<br>在矫正期间还要注意保持合理的饮食，尽量不要吃一些过于坚硬的食物，减少对牙齿的刺激，还要注意养成良好的睡眠习惯，能够减少口腔疾病的发生。

17.牙齿矫正会造成龋齿吗?	
A	牙齿矫正本身不会直接导致龋齿，但矫正器可能影响口腔卫生的维护，从而增加龋齿风险。固定矫正器容易积存食物残渣和牙菌斑，尤其在托槽、钢丝周围，如果清洁不彻底，细菌繁殖会导致龋齿。预防龋齿的关键是良好的口腔卫生习惯，如饭后刷牙、使用牙线和间隙刷，以及定期使用含氟牙膏或漱口水。同时，定期口腔检查和洁牙也有助于防止龋齿发生。因此，在矫正期间应特别注重口腔清洁和饮食控制，以降低龋齿风险。
B	牙齿矫正本身不会直接导致龋齿，但由于矫正器具的存在，清洁牙齿变得更加困难，容易积聚食物残渣和牙菌斑，增加了龋齿的风险。若口腔卫生不佳，细菌在牙齿表面繁殖，形成酸性物质，进而腐蚀牙釉质，导致龋齿。因此，在矫正期间，需要特别注意口腔清洁，定期使用牙刷、牙线以及漱口水，避免食用粘性或高糖食物，定期去牙科检查，确保牙齿健康。
C	牙齿矫正本身不会直接导致龋齿，但在矫正过程中确实存在增加龋齿风险的因素。矫正器的存在会使清洁牙齿变得更加困难，容易在托槽周围积累菌斑和食物残渣。如果没有养成良好的口腔卫生习惯，确实可能导致龋齿。为预防龋齿，建议：1. 每天认真刷牙至少两次，使用正确的刷牙方法；2. 配合牙线、水牙线等工具清洁牙缝；3. 避免过多甜食；4. 定期复诊检查。只要注意口腔卫生，规范进行矫正治疗，龋齿的风险是完全可控的。
D	在固定式正畸（牙箍）治疗期间，会出现早期龋齿或牙齿上的脱钙病变（demineralised lesions, DLs；也称为白斑病变）。 氟化物可减少易感个体（包括正畸患者）牙齿的腐蚀。
E	其实，矫牙本身是不会导致牙齿龋坏的。 如果在矫治期间患有龋齿，多半是由于 清洁不到位 ，导致 口腔卫生状况差 ，比如进食后，食物残渣留在矫治器和牙齿缝隙里，没有彻底清理，导致细菌的滋生，从而出现牙齿龋坏。 矫牙时没做好口腔清洁，还会引起牙菌斑的形成和堆积，严重的会出现 牙结石 、 牙龈红肿 及 牙周炎 等口腔疾病，进而影响 矫治的进度与效果。
F	牙齿矫正本身不会造成龋齿，但如果矫正期间口腔清洁不到位，容易导致食物残渣堆积和细菌滋生，从而增加龋齿风险。
G	牙齿矫正本身不会直接造成龋齿，但矫正过程中可能增加患龋齿的风险。因为矫正器使用粘结剂固定托槽，易使食物残渣滞留牙缝，若口腔卫生不佳，易滋生细菌导致龋齿。因此，牙齿矫正期间需特别注意口腔卫生，定期清洁口腔，以降低龋齿风险。
H	矫正牙齿容易得蛀牙，需要做好口腔卫生工作。如果牙齿出现了参差不齐的情况，可以选择矫正牙齿的方式进行治疗，主要是佩戴钢丝牙套或者是隐形牙套，通过牙套产生的牵引力，能够使牙齿进行移动，从而能够达到比较好的矫正效果，在矫正牙齿期间，由于佩戴了牙套，很有可能会导致食物残渣的残留，而且不容易清洁，从而会诱发局部的细菌滋生，增加蛀牙的几率。<br>做牙齿矫正的时候选择正规的口腔科门诊，能够提高矫正的成功率。

18.牙齿矫正会导致牙齿松动吗？	
A	牙齿矫正通常不会导致永久性牙齿松动，但在矫正过程中可能出现轻微松动，这是正常现象。矫正时牙齿在正畸力的作用下移动，会伴随牙周膜的重建和骨质的吸收与再生，可能导致短期内牙齿感觉不稳定。但这种松动通常是可逆的，牙齿会在矫正完成后通过骨改建重新稳定。如果正畸力量过大或操作不当，可能对牙周组织造成损害，增加牙齿永久松动的风险。因此，牙齿矫正需在专业正畸医生指导下进行，避免相关并发症。
B	牙齿矫正过程中，牙齿可能会出现轻微松动的情况，但这是正常的生理反应。在矫正过程中，牙齿需要移动到新的位置，牙槽骨和周围组织会逐步适应这些变化。在施加矫正力时，牙齿会暂时松动，随着时间的推移，牙槽骨会重新生长并固守牙齿。这种松动通常是暂时的，并且在牙齿到达最终位置后会逐渐恢复稳定。如果松动情况持续或者有其他不适，应该及时咨询牙医。保持良好的口腔卫生和定期复诊有助于确保矫正过程的顺利进行。
C	牙齿矫正确实会引起暂时性的牙齿松动，这是正常的生理现象。矫正过程中，牙齿周围的牙周膜会受到持续的力量作用，导致牙槽骨重塑，从而使牙齿在骨组织中移动到理想位置。这种松动感通常是轻微的，随着治疗的进行会逐渐稳定。整个矫正过程结束后，牙齿会重新固定在新的位置上，最终达到稳定状态。但如果出现明显的松动或疼痛，应及时咨询正畸医生，调整治疗方案。注意在矫正期间保持良好的口腔卫生，避免剧烈咀嚼，这样可以减少不适感。
D	正畸期间，牙齿处于移动状态，会出现生理性的松动，这是牙槽骨正在进行改建的过程。据统计，正畸治疗结束后仍会有短暂的松动期，一般为几天到几周不等，此阶段称为“松动期”，之后牙齿就会逐渐恢复到原来的稳定状态。 牙齿松动的主要原因是什么？
E	病理性运动：通常定义为牙齿移位，当维持生理牙齿位置的因素之间的平衡受到牙周疾病的干扰时发生（简而言之，是由于疾病引起的松动）。生理性运动：通常是指在牙齿上引起牙齿移位的力（总之，这是受力造成的安全性松动）。我们正常的牙齿也有一定程度的松动。例如，当我们用食指拉动牙齿时，我们显然会感到牙龈疼痛。此时，牙齿已经在轻微移动。这种松动非常轻微，几乎不明显。牙齿矫正是通过外部加力的方式，让不整齐的牙齿按照治疗计划，安全而缓慢地移动，排列整齐，它属于生理运动。这种松动不会对牙齿本身及其支撑组织（即牙龈，牙周膜等）造成病理损害，这是正常现象。
F	牙齿矫正在专业正畸治疗的规范操作下，并不会导致牙齿永久性松动。牙齿在矫正过程中受力移动，会有一定范围的松动，这是正常的生理反应。矫正完成后，牙齿会通过自身的修复能力使牙周膜重新附着而变稳固，不会发生永久性的损伤。因此，正常情况下，牙齿矫正不会导致牙齿松动。
G	牙齿矫正过程中可能会出现牙齿松动，这主要是牙齿移动过程中的正常现象，通常会逐渐消失，牙齿会在新的位置上稳固下来。牙齿松动受到多种因素影响，包括口腔健康状况、矫正方案、矫正器使用、患者配合度及口腔卫生习惯等。因此，应选择正规医院和专业医生进行矫正。
H	一般情况下，牙齿矫正会导致牙齿松动。如果患者牙齿出现排列不齐、错位严重等情况，此时可能需要进行牙齿矫正。通常医生会在患牙部位安装矫正器进行治疗，此类手术的目的是让排列不齐的牙齿，逐渐移动到提前预设好的位置。从而达到让牙齿整齐排列的效果，但是移动过程中牙槽骨内的破骨、成骨等细胞会在牙齿受力后进行骨质吸收以及重建，从而让牙周膜重组，一般牙齿在外力的影响下会出现松动迹象。通常情况下，这是牙齿矫正过程一种正常的现象，不需要特殊处理。当矫正一段时间后，牙周组织得到充分修复，此时牙齿松动迹象会逐渐消除。在术后如果牙齿出现松动较为严重时，可能是由于矫正过程中速度过快、加力过大导致，建议患者及时到医院确诊治疗，防止病情加重。患者术后要尽量食用一些较软的食物，比如面条、小米粥等，避免食用过硬的食物后影响牙齿松动恢复。

19.牙齿矫正会很疼吗？	
A	牙齿矫正并不会一直疼痛，但在某些阶段可能会感到不适。初次戴矫正器时，由于牙齿逐渐适应压力，可能会有轻微酸痛，通常持续几天。此外，每次调整矫正器后，也可能出现短暂的不适。大部分患者表示，这种疼痛是可以忍受的，类似轻微的紧绷感。为减轻不适，可以尝试软质饮食、使用牙蜡防止口腔溃疡或按需使用止痛药。矫正后，牙齿逐渐移动到正确位置，不适感会减轻。良好的护理和与正畸医生的沟通可以帮助缓解不适，使整个过程更加舒适。总之，牙齿矫正虽有轻微不适，但效果值得期待。
B	牙齿矫正会有一定的不适感，但痛感通常是可以忍受的。在矫正初期，特别是戴上托槽和钢丝的头几天，牙齿可能会感觉酸痛或紧绷。这是因为牙齿在逐渐移动，牙齿和牙龈的压力变化会引起不适。随着时间的推移，牙齿适应了矫正力量，疼痛感会逐渐减轻。可以通过使用温盐水漱口、服用非处方止痛药等方法来缓解不适。整体而言，牙齿矫正的痛感是暂时的，耐心适应后，最终会带来美丽的笑容。
C	牙齿矫正的疼痛程度因人而异。刚开始戴牙套的 1-3 天通常会感觉疼痛和不适,这是因为牙齿开始移动,会对牙周组织产生压力。疼痛一般在一周内会明显缓解。每次复诊调整牙套后也可能会短暂疼痛 1-2 天。<br>日常生活中,建议选择软质食物,避免过冷过热的食物刺激。如果疼痛明显,可以遵医嘱服用止痛药。随着治疗的进行,身体会逐渐适应,疼痛感会越来越轻。总的来说,矫正过程的疼痛是可以忍受的,不会影响正常生活。
D	患者会感到不同程度的不适，主要是由于矫治器对牙齿的压力以及托槽与粘膜的摩擦。这取决于安装的专业人员、所需调整的类型，当然也取决于患者的疼痛阈值。 建议至少等待 2 或 3 天，如果疼痛没有减轻，可以进行相应的调整。 最初几天最不舒服，但随着时间的推移，不适感会逐渐减轻。
E	矫正牙齿在短期内有疼痛是很正常的，这是身体的一种正常反应，不用过于担心。 当牙齿接受这种轻微而持续的外力时，牙齿本身是不会痛的，但会使牙周膜受到拥挤，拉扯，所以才会让人感觉疼痛。 痛感维持时间一般不超过 10 天，大多数人 3-5 天即可缓解。 戴牙套前需要拔牙，会有一点点痛。 有些情况下，医生会建议有的患者朋友先进行拔牙再做矫正。 而在拔牙之前会打麻醉针，所以在拔牙过程中完全感受不到一点痛。
F	牙齿矫正过程中可能会有一些不适，但通常不会很疼。初期佩戴牙套时，牙齿可能会感到轻微的紧绷和疼痛，这是因为牙齿正在逐渐移动到新的位置。这种不适感一般在几天内会逐渐减轻。矫正过程中，定期调整牙套时也可能会有短暂的不适，但通常可以通过服用非处方止痛药来缓解。每个人的体验不同，如果感到疼痛难以忍受，应及时咨询牙医。
G	牙齿矫正通常不会产生剧烈疼痛。在矫正初期，患者可能会感到牙齿酸痛或不适，这属于正常现象，一般持续 3-5 天，且大部分人可以忍受。若疼痛持续或加剧，应及时就医，检查是否存在感染或矫治力量过大的情况。
H	矫正牙齿并不是很痛，早期会产生不适，慢慢能够自然。矫正牙齿根据自身牙齿发育情况，选择相关矫正方法，比如普通正畸矫正，选择钢丝牙套，也可选择无托槽隐形矫正器，从外表上并不能看出，有利于达到完全隐形目的增加口腔美观，如果牙齿畸形比较严重，比如上颌后突或者下颌前突，此时需要进行正颌外科牙齿矫正。日常定期到医院复诊，多喝些温热的白开水，避免经常吃高糖食物或者含糖饮料。

20.牙齿矫正后还有牙缝怎么办?	
A	牙齿矫正后仍有牙缝可能与矫正设计、牙齿形态或牙周状况有关。首先，与正畸医生复查，评估矫正计划是否需要调整，如橡皮链或精细调整牙齿位置。其次，若因牙齿形态导致牙缝，可通过牙齿修复手段如树脂贴面或烤瓷冠填补。最后，保持良好的口腔卫生，预防牙周病引发牙齿松动导致缝隙增大。及时处理，避免影响矫正效果和美观。
B	牙齿矫正后出现牙缝的原因可能是矫正过程中牙齿移动导致的，或者原本存在的牙缝没有完全关闭。如果矫正结束后仍有明显的牙缝，建议首先咨询正畸医生，检查牙齿是否恢复稳定。医生可能会根据情况推荐使用保持器维持矫正效果，或进行适当的调整。如果牙缝较大，可能需要考虑进一步的矫正治疗，或者进行修复，如牙齿贴面、填充等。保持良好的口腔卫生，避免不良习惯（如咬唇或咬物）有助于保持矫正效果。
C	牙齿矫正后出现牙缝是比较常见的情况，需要从以下几个方面来解决:<br>首先建议及时咨询正畸医生，评估牙缝的具体原因。可能是由于牙齿移动过程中出现了轻微偏差，或是个人口腔护理习惯的问题。<br>医生可能会建议采取以下措施:一是戴保持器来预防牙齿进一步移位；二是考虑进行微调或填充治疗来修复牙缝；三是调整刷牙和使用牙线的方式，保持良好的口腔卫生习惯。如果牙缝较小且不影响美观和功能，也可以选择继续观察，因为牙齿可能会随着时间慢慢就位。重要的是要遵医嘱定期复查。
D	矫正完牙缝的解决方法 1：全瓷美白贴片。全瓷美白贴片能够将大黑三角牙缝关闭起来，同时改善牙齿颜色、形状、外观。矫正完牙缝的解决方法 2：树脂填补。用树脂填补也可以改善外观，但由于材质特性，长期使用易有色素染色，导致树脂染色后变得黑黑黄黄，与本身牙齿有明显色差。矫正完牙缝的解决方法 3：修磨齿质再将牙齿并拢。医师并不会在矫正前就修磨齿质，而是在矫正中、后期，修磨牙齿之间接触点的齿质，再让牙齿俩俩互相靠拢，藉以关闭牙缝。
E	在矫正完牙齿后，基本上不会存在比较大的缝隙，如果只是比较小的牙缝，可能是医生根据患者口腔情况选择的合适牙间距。 这时存在一定的牙缝，若无其他不适，一般属于正常的情况。 在正畸结束后，如果牙齿之间仍然有比较大的缝隙，或存在其他不适，比如出现牙疼、牙龈红肿、牙齿松动等情况，则属于不正常的情况。 可能与正畸治疗过程中牙套不紧、固定位置不佳、患者习惯不佳等因素有关。
F	牙齿矫正后出现牙缝可以通过以下几种方法解决：1. 正畸治疗，通过调整牙齿位置消除缝隙；2. 瓷贴面，覆盖牙缝改善美观；3. 牙冠修复，适用于缝隙较大或牙齿损伤严重的情况。建议咨询专业牙医，根据具体情况选择合适的修复方式。
G	牙齿矫正后仍有牙缝，可以考虑再次矫正，佩戴牙套如钢丝牙套或隐形牙套以改善牙缝；或者选择牙贴面治疗，通过打磨牙齿并制作牙贴面来恢复牙齿整体形象。平时应保持良好的口腔卫生习惯，定期复诊并遵循医生指导。
H	矫正后牙齿有缝可以通过再次矫正或者是做牙贴面的方式来改善。<br>1、再次矫正：矫正后牙齿有缝一般可以考虑再次佩戴牙套的方式进行改善，比如钢丝牙套或者是隐形牙套，通过牙套产生的拉力能够使牙齿进行移动，从而能够改善牙齿的缝隙。2、牙贴面：如果属于轻度的牙缝情况，也可以通过做牙贴面的方式进行治疗，主要是通过对原有的牙齿进行打磨，去除少量的牙体组织，然后制作牙贴面，从而能够恢复整体的形象以及正常的咀嚼功能。平时还要保持口腔的清洁卫生。

21.牙齿矫正后容貌会有什么变化?	
A	牙齿矫正后，容貌可能会发生显著变化，主要体现在以下几个方面：首先，牙齿排列整齐可以改善笑容，使面部更协调；其次，矫正咬合问题可能改变下颌位置，从而影响脸型，尤其是下颌突出或凹陷的情况；此外，矫正牙齿可能改善唇部形态和闭合状态，使嘴唇更自然；最后，通过调整面部比例，整体面容可能更加立体。效果因人而异，需综合考虑骨骼发育、牙齿状况和矫正方法。
B	牙齿矫正后，容貌通常会发生一些显著的变化。首先，矫正后的牙齿排列整齐，笑容更加自然和自信。牙齿的对称性改善可以使脸部轮廓更加平衡，尤其是颌部和下巴线条的调整，往往能提升面部的和谐美感。其次，口腔健康得到改善，咬合问题得到矫正，减少了因牙齿不整齐导致的面部压力。长远来看，牙齿矫正不仅改善外貌，也有助于提升自信心，给人留下更好的第一印象。
C	牙齿矫正后的容貌变化主要体现在以下方面:面部轮廓会变得更加协调。通过调整牙齿位置,可以改善面部软组织的支撑,使脸型更加匀称。特别是对于龅牙或地包天的患者,矫正后下巴和嘴唇的位置会更加自然。笑容会更加美观。整齐的牙齿排列能让笑容看起来更加迷人,增加面部表情的感染力。不整齐的牙齿往往会让人在社交时感到不自信,矫正后这个问题就能得到改善。口唇关系会得到优化。通过调整牙齿位置,可以改善上下唇的闭合状态,使说话和休息时的表情更加自然。同时还能改善发音,让语言表达更加清晰。
D	1、改善侧面外观。尤其是对地包天，龅牙等情况，在矫正后从侧面看，唇部突出的位置会后缩，看上去比较自然，从侧面来看脸型会比较好看。<br>2、改善脸部宽窄。对于部分现象需要采取拔牙矫正的情况，在经过矫正后脸部的宽窄会有所变化。<br>3、改善脸型。矫正期间会出现饮食不便、牙齿疼痛等影响，由于矫正引起的进食不便、疼痛感和产生的心理压力，会让人变瘦，导致脸部变瘦。
E	"矫正牙齿会对脸型有变化，牙列过度拥挤及明显前凸时，可以拔除智齿及双尖牙，获得充足的矫治间隙。 拔牙创口的牙槽骨逐渐萎缩、吸收后，下颌角度减小，面部线条变柔和。 矫治后期排齐牙列，整平牙弓，关闭剩余间隙后牙弓缩短。 同时前牙唇倾度明显改变，形成协调的面部比例关系。 前牙过度唇倾时，可以进行种植支抗，压低临床牙冠，内收前牙，改善开唇露齿的现象。
F	牙齿矫正后，容貌可能变得更加和谐美观。牙齿排列整齐，笑容更加自然，可能提升面部轮廓，改善咬合功能，甚至对脸型产生微妙影响，如下巴线条可能更清晰。
G	牙齿矫正后容貌可能会有所变化，特别是对面部轮廓有积极影响。矫正可以改善上颌前突、下颌前突等问题，使面部更加协调美观。不过，具体变化因人而异，且矫正主要影响的是牙齿和颌面比例，对整体容貌的改变有限。
H	牙齿矫正后，容貌变化主要源于以下几点：1. 下颌回缩：对于下颌前突（地包天），矫正可使突出的下颌向后缩回，前突程度减轻、恢复到正常位置。2. 上颌回缩：对于上颌突出（龅牙），根据年龄和情况，通过截骨或移动上颌骨改善脸型。3. 嘴巴闭紧、牙龈不外露：矫正可改善双颌前突、双唇明显前突、不能自然紧闭、牙龈外露过多。并非所有的正畸治疗均能改变颌面部形态，若牙齿因素导致面部外观畸形，没有引起颌骨改变，正畸后容貌的变化并不明显。进行正畸治疗后，要注意口腔卫生，避免进食过硬的食物，还需按时复查、及时调整。

22.牙齿矫正有哪些方法？	
A	牙齿矫正方法主要包括以下几种：1. **金属托槽矫正**：传统且经济实用，适合大多数人；2. **陶瓷托槽矫正**：美观性较高，但成本略高；3. **隐形矫正**：如 Invisalign，透明美观，易摘戴，适合轻中度畸形；4. **舌侧矫正**：托槽位于牙齿内侧，隐蔽性强，技术要求高；5. **功能性矫正器**：适用于儿童，矫正骨性问题；6. **外科手术结合矫正**：针对严重骨性畸形。选择需结合个体需求与医生建议。
B	牙齿矫正的方法有多种，常见的包括传统金属牙套、陶瓷牙套、隐形矫正和舌侧矫正。金属牙套是最常见且价格较为亲民的选择，通过金属托槽和弓丝调整牙齿位置。陶瓷牙套与金属牙套相似，但颜色更接近牙齿，较为美观。隐形矫正使用透明的塑料托槽，几乎不被察觉，适合对外观有较高要求的人。舌侧矫正则是将矫正装置安装在牙齿内侧，外部不易看见。每种方法都有其优缺点，选择时需要根据个人需求、牙齿状况和预算综合考虑。
C	牙齿矫正主要有以下几种方法：传统金属托槽矫正器是最基础的选择，价格相对较低；陶瓷托槽外观更美观，但价格较高；舌侧矫正器安装在牙齿内侧，美观性最好；隐形矫正牙套透明可摘戴，舒适度高，但仅适用于轻中度错颌。此外还有扩弓器、功能性矫治器等辅助装置。选择合适的矫正方式需要考虑错颌程度、年龄、预算等因素，建议在正规医院进行诊断后再决定。
D	1.托架托架。 这是最最传统的固定正畸类型。 它们对咬伤甚至打击的抵抗力最强。2.托架陶瓷. 所用的材料使设计比通常的金属牙套更具有美感。3.托架蓝宝石. 它们是最隐蔽的，几乎看不见。4.舌侧正畸. 一种放置在牙齿内侧的隐形正畸，从而减少美观问题。
E	1.数字化无托槽隐形矫正。特点：由高分子材料制成，外表近乎透明，厚度小于 1mm，佩戴舒适，可自由摘戴，吃饭刷牙都可以摘掉，更容易维护口腔健康。2.数字化陶瓷托槽半隐形矫正。特点：半隐形矫正采用的托槽近乎透明，粘在牙齿上隐蔽性较好，陶瓷托槽特有的圆润化处理减少对口腔黏膜的不良刺激，所以佩戴更舒适。3.数字化舌侧托槽隐形矫正。舌侧矫正是把矫正器全部安装在舌侧，隐蔽性极好，对疑难杂症畸形牙又渴望隐形的患者能起到很好的疗效。4.数字化金属托槽隐形矫正。金属托槽矫正是目前使用最广泛的一种方式，由于金属托槽的摩擦力极低，牙齿的移动更为容易，可以有效降低拔牙率。
F	牙齿矫正主要有以下几种方法：传统金属牙套、陶瓷牙套、隐形矫正器（如隐适美）、自锁牙套以及舌侧矫正器。每种方法都有其特点和适应症，需根据个人牙齿状况和需求选择合适的矫正方案。
G	牙齿矫正的方法主要包括：预防性矫正、活动矫治器矫正、固定矫治器矫正（如金属托槽、陶瓷托槽）、隐形矫正以及正颌手术。具体选择哪种方法，需根据患者的牙齿情况和医生建议来确定。
H	1、常规矫正：使用金属脱槽的方式施加轻微的力度进行移动以及排挤达到理想位置和效果。2、自锁托槽矫正：使牙齿慢慢回归到正常位置，通过矫正技术的方式进行优化和改进，较好地保护牙齿。3、隐形矫正：通过一根淡淡的金属丝，可以在不察觉的情况下是牙齿排列整齐，也能够增加咀嚼功能。

23.牙齿矫正前需要做哪些准备？	
A	在进行牙齿矫正前，需要做好以下准备：首先，选择正规的口腔医院或诊所，咨询专业正畸医生，确定矫正方案。其次，进行全面的口腔检查，包括牙齿、牙周和口腔健康情况，处理龋齿、牙结石等问题，确保口腔无炎症。第三，拍摄全景片和头颅侧位片，辅助医生分析咬合关系和骨骼情况。第四，调整日常饮食和清洁习惯，避免过硬食物，学习正确的口腔清洁方法。最后，明确矫正周期、费用及注意事项，准备好心理和时间规划，以积极配合治疗。
B	牙齿矫正前需要进行充分的准备。首先，需预约正畸医生进行详细检查，评估牙齿和口腔健康状况，可能需要拍摄 X 光片和口腔模型。医生会根据检查结果制定个性化的治疗计划。其次，清洁牙齿是必须的，确保没有牙结石和龋齿，避免矫正过程中产生感染。此外，若有严重的咬合问题，可能需要进行拔牙或其他手术治疗。最后，了解治疗过程中的注意事项，如定期复诊、饮食禁忌等，为矫正过程做好全面准备。
C	在开始牙齿矫正前,需要做好以下几个方面的准备:首先要进行全面的口腔检查,包括拍摄口腔 X 光片、口腔 CT 等,评估牙齿、牙龈和颌骨的状况。如有蛀牙或牙周炎需要先进行治疗。其次是进行口腔卫生清理,包括洗牙去除牙结石和牙菌斑,学习正确的刷牙方法和使用牙线。第三,需要和医生详细沟通矫正方案,了解治疗周期、费用和注意事项。选择合适的矫正方式,如传统金属托槽、陶瓷托槽或隐形牙套。最后要调整心态,做好长期治疗的准备,保持耐心和信心。戴牙套初期可能会有不适,要提前做好心理准备。
D	牙医进行初步牙科检查，口腔印模，拍 X 光片，并计划牙齿矫正治疗。 将进行洗牙， 填充蛀牙， 必要拔阻生牙，准备牙齿矫正或者称之为口腔清理。 口头清理完成后， 这将是在患者牙齿上安装牙齿矫正工具，为了对齐牙齿。 每 2-3 个月去看牙医检查， 定期跟进（取决于工具类型）。
E	1、明确自己想通过戴牙套解决什么问题，比如：龅牙？地包天？牙齿不齐？还是因为爱美，想通过矫牙让脸型好看些？<br>2、矫牙原理：人的牙槽骨一生都处于变化之中，医生利用正畸力作用在牙齿上，使牙齿在骨头里面移动，最终使牙齿排列整齐。<br>3、牙齿矫正我们不可避免的一件事就是：戴牙套。我们对牙套的种类、区别、利弊都做好分析后，才能与医生沟通选择适合的方案。
F	牙齿矫正前，需要进行口腔检查，确定矫正方案；拍摄口腔 X 光片，了解牙齿和颌骨状况；做好口腔清洁，治疗牙周病等口腔疾病；与医生沟通，了解矫正过程、预期效果和可能风险。
G	牙齿矫正前需要做以下准备：进行全面的口腔检查，包括牙齿、牙龈、口腔黏膜等；拍摄 X 光片；制取牙齿模型；保持口腔清洁，去除牙齿表面的牙菌斑和牙结石；与医生沟通确定适合的矫正方案；做好心理准备，积极配合治疗。
H	1. 进行检查并确定治疗方案：口腔 X 线片检查，取牙齿咬合模型。<br>2. 进行手术前准备：洗牙、补牙、减数拔牙等。<br>3. 佩戴矫正器：可能会有不适感和磨嘴的情况，大约需要一个星期适应。在矫正期间需维护口腔卫生并定期复查。

24.牙齿矫正可以只矫正半口牙吗?	
A	牙齿矫正是否可以仅矫正半口牙取决于具体情况。一般来说，矫正全口牙齿可以更好地调整上下牙弓的咬合关系，避免因单侧矫正导致牙齿排列失衡或咬合问题。然而，在某些情况下，如仅一侧牙齿出现轻微拥挤或排列异常，且不会对整体咬合和美观造成明显影响时，可以考虑半口矫正。最终方案应根据正畸医师的全面评估制定，患者需结合医生建议和自身需求做出决定，以达到理想的矫正效果。
B	牙齿矫正通常是针对整口牙齿进行调整，但在某些情况下，确实可以只矫正半口牙。这种做法通常适用于那些只有单侧牙齿存在问题的患者，比如一侧有严重的牙齿错位或咬合问题。半口牙矫正的优点是治疗周期较短，费用较低。然而，这种治疗方法需要医生根据具体情况评估是否适合，因为单侧矫正可能会影响整体咬合关系，导致长期的不适或其他问题。因此，在选择是否进行半口牙矫正时，建议咨询专业的牙科医生。
C	牙齿矫正理论上可以只矫正半口牙,但并不建议这样做。主要原因是:1. 上下牙齿是一个整体,相互咬合关系密切。单独矫正半口牙可能会影响整体咬合,导致咬合不当。2. 半口矫正可能造成牙齿受力不均匀,影响矫正效果和稳定性。3. 矫正后的半口牙与未矫正的半口牙在美观上会存在差异。<br>因此,建议进行全口矫正,这样更有利于达到理想的咬合效果和美观效果。具体是否可以半口矫正,还需要根据个人具体情况咨询专业医生评估。
D	在牙齿矫正的医学上表明是可以做半口牙矫正的，但是从临床的数据上发现，只有极少一部分人适合半口牙齿矫正，而且还有的在矫正后牙颌出现了不正常的关系，达不到预期的效果，还要进行二次矫正。
E	口腔正畸医生表示，以专业矫正治疗的立场来说，只做半口牙齿矫正的做法，容易产生上下颌牙齿咬合无法对位的问题，从而导致矫正失败。 比方说，只做上颌矫正，不做下颌，那么上颌在排齐后，牙弓已经形成比较稳定的卵圆形形状，但是下颌还是照样拥挤不整齐，形成不规则的上下咬合关系，可能垂直向和水平向的咬合关系都不会很好，这样的功能性咬合不良会给牙齿矫正的效果大打折扣。
F	牙齿矫正通常不建议只矫正半口牙。因为矫正的目的不仅是排齐牙齿，更重要的是调整咬合关系，确保牙齿功能和健康。仅矫正半口牙可能导致咬合关系紊乱，影响咀嚼功能，甚至导致牙齿松动等问题。因此，专业正畸医生一般不会建议只矫正半口牙齿。
G	牙齿矫正可以只矫正半口牙，但一般不建议，因为这可能对咬合关系产生不良影响，增加矫正后复发的几率。牙齿矫正不仅仅是排齐牙齿，更重要的是维护牙体本身的咀嚼功能。因此，在决定矫正方案时，应充分考虑咬合关系，必要时选择全口矫正。
H	牙齿矫正不能只矫正一半：1. 只矫正一半会影响美观和咬合关系：牙齿矫正需要排列整齐，只矫正一半会影响美观度和咬合关系。2. 只矫正一半会影响口腔健康：只矫正一半会导致咬合关系紊乱，进而影响口腔健康和咀嚼功能。3. 需要选择正规医院和医生：建议选择正规的口腔医院和有经验的医医生进行咨询和治疗。医生会根据具体情况和需求，制定矫正方案，确保牙齿矫正的效果和口腔健康。

25.牙齿矫正器有哪些类型?	
A	牙齿矫正器主要包括以下类型：1. **金属托槽矫正器**：常用传统矫正器，耐用，适用于复杂病例，但外观显眼。2. **陶瓷托槽矫正器**：美观度高，颜色接近牙齿，但价格较高。3. **舌侧矫正器**：安装在牙齿内侧，隐蔽性好，适合注重外观的人群。4. **隐形矫正器**：透明可摘戴，舒适且美观，适合轻中度矫正。5. **功能性矫正器**：常用于青少年，改善骨骼发育问题。<br>选择矫正器需根据牙齿情况、需求及预算决定，建议咨询专业正畸医生。
B	牙齿矫正器主要有以下几种类型：1. **金属托槽矫正器**：传统的矫正方式，采用金属托槽和弓丝，效果显著，但外观相对明显。2. **陶瓷托槽矫正器**：类似金属托槽，但采用透明或接近牙色的陶瓷材料，外观更为美观。3. **舌侧矫正器**：托槽安装在牙齿内侧，外人难以察觉，适合注重外观的人群。4. **隐形矫正器**：通过一系列透明塑料矫治器逐步矫正，几乎不可见，舒适性较好。5. **自锁托槽矫正器**：采用自锁机制，减少了调整次数，提高治疗效率。
C	牙齿矫正器主要分为四大类型：1. 传统金属托槽矫正器：使用金属托槽和钢丝，是最常见且价格相对较低的选择，但外观较明显。2. 陶瓷托槽矫正器：采用陶瓷材料制作，外观较为美观，但价格较高，且容易染色。3. 隐形矫正器：使用透明可摘式牙套，美观且舒适，适合轻中度牙齿不齐，但费用较高。4. 舌侧矫正器：将托槽安装在牙齿内侧，完全隐形，但价格最贵，且初期可能影响发音和舌头适应。选择合适的矫正器类型需要考虑个人牙齿情况、预算和美观需求，建议在专业医生指导下做出决定。
D	牙齿矫正的牙套种类目前主要分为「传统矫正器」、「自锁型矫正器」、「舌侧矫正器」与「隐形牙套」四大类，再根据材质分成「陶瓷」、「树脂」或「金属」三种。
E	1、传统金属矫正器：传统金属矫正器是最常见的矫正器之一，由金属材料制成，包括金属托槽和金属线。这种矫正器可以有效地将牙齿移动到正确的位置，并具有较强的耐用性和可调节性。2、陶瓷矫正器：陶瓷矫正器是一种不可见的矫正器，由透明陶瓷材料制成，可以与牙齿颜色相似，减少了对外观的影响。它比金属矫正器更美观，但相对脆弱一些。3、隐形矫正器：隐形矫正器是一种透明的塑料矫正器，如 Invisalign?。它是一种可拆卸的矫正器，能够自由地摘下和穿戴。由于透明材料的使用，它几乎不可见，对外观影响最小。然而，隐形矫正器适用于轻度到中度的矫正问题，对于复杂的牙齿问题不太适用。4、舌侧矫正器：舌侧矫正器是一种新型的矫正器，与传统矫正器相比，它将托槽放置在牙齿舌侧，减少了对外观的影响。舌侧矫正器较为舒适，并且会降低舌头的残留物。然而，舌侧矫正器需要更长的矫正时间和更高的技术要求。5、自锁矫正器：自锁矫正器是一种特殊设计的矫正器，其托槽内部具有锁定机构，可以自动锁定弓丝，无需使用传统矫正器所需的橡皮圈或金属扣子。自锁矫正器具有较低的摩擦力，能够快速矫正牙齿，减少牙齿矫正过程中的不适感。
F	牙齿矫正器主要有以下几种类型：1. 金属托槽矫正器，技术成熟、价格相对便宜，但美观性较差；2. 陶瓷矫正器，美观性好，适合金属过敏者，但价格较高；3. 隐形矫正器，美观、舒适，可自行摘戴，但价格较高；4. 舌侧矫正器，美观性最好，但对医生技术要求高，价格较高。
G	牙齿矫正器主要有以下几种类型：金属托槽矫正器、陶瓷托槽矫正器、自锁托槽矫正器、舌侧矫正器、无托槽隐形矫正器以及功能性矫正器。选择时，需根据个人牙齿情况、美观需求及医生建议综合考虑。
H	牙齿矫正器的种类：1.固定性矫正器：无法自行取下，可改变牙冠以及牙根位置。2.功能性矫正器：可自行摘戴，适用于替牙期等生长发育期间。3.隐形矫正器：透明光滑，隐蔽性好，佩戴后美观舒适，并可减少对口腔的影响，避免并发症。

26.在进行牙齿矫正时，隐形牙套好还是钢丝牙套好？	
A	隐形牙套和钢丝牙套各有优劣，选择需根据个人需求与牙齿情况决定。隐形牙套美观，适合不希望矫正过程被注意的人群，佩戴舒适且易清洁，但价格较高，适用于轻中度错颌畸形。钢丝牙套适应范围广，能解决复杂牙齿问题，费用相对低廉，但外观较明显，可能影响口腔卫生且佩戴初期较不适。建议咨询专业正畸医生，根据具体牙齿问题和经济状况综合评估选择最适合的方案。
B	隐形牙套和钢丝牙套各有优缺点。隐形牙套的最大优势是美观，佩戴时几乎不易察觉，且可以在进食和清洁时取下，方便卫生。然而，它的价格较高，且需要严格按照医生的指导佩戴，效果可能因患者配合度而有所不同。钢丝牙套的优势是效果显著，适用于大多数牙齿畸形情况，且价格相对较低。不过，由于固定在牙齿上，外观上不如隐形牙套美观，清洁也较为麻烦。总的来说，选择哪种牙套要根据个人的需求、预算和医生的建议来决定。
C	选择隐形牙套还是钢丝牙套需要根据个人情况来决定。隐形牙套的优势在于美观、舒适度高、可摘戴方便清洁，但价格较贵，且不适用于严重错颌畸形的病例。钢丝牙套价格相对较低，矫正效果可靠，适用于各类牙齿畸形，但外观不佳，初期会感到不适，清洁相对困难。建议您根据自己的牙齿问题严重程度、经济预算、生活工作环境等因素，咨询正规医院的正畸专科医生做出选择。
D	对于那些需要复杂矫正方案的患者来说，传统钢丝牙套可能是更好的选择。 由于其固定在牙齿上的性质，患者在饮食上的限制较少，不需要频繁摘戴，从而减少了忘记佩戴或丢失矫治器的风险。 此外，钢丝牙套对牙齿移动的控制能力较强，适合解决一些严重的咬合问题。
E	矫正牙齿时，需要根据患者自律性、年龄来选择牙套，主要根据牙齿移动的类型和方式进行选择， 没有戴哪种牙套好或不好的说法。 如果需要所有牙齿向前移动，可能选择钢丝矫治的效果更好，因为钢丝牙套让牙齿向前移动的效率比较高。 如果需要牙齿向后移动，尤其是后牙需要向后移动，这时候隐形牙套的效率可能比较高。 所以牙齿移动的方式，是决定选哪种牙套比较重要的因素。
F	隐形牙套和钢丝牙套各有优势，选择应根据个人需求。隐形牙套美观、舒适，适合轻度至中度矫正。钢丝牙套力度大，矫正快，适合复杂或严重错颌。
G	隐形牙套和钢丝牙套各有优势。隐形牙套美观舒适，适合轻度到中度矫正，且对口腔卫生影响小，但价格较高。钢丝牙套矫正力度强，适合重度牙齿畸形，价格更实惠，但可能影响美观和舒适度。选择哪种牙套需根据个人牙齿情况和需求综合考虑。
H	牙齿矫正选择隐形牙套还是钢丝牙套：1. 隐形牙套适用于轻度到中度矫正需求的患者，不影响美观和舒适度，但需要患者具有较高的依从性。<br>2. 钢丝牙套适用于牙齿畸形严重的患者，具有更强的矫正力度，能够更有效地控制牙齿的移动方向和速度，通常比隐形牙套更实惠。

27.牙齿矫正期间有什么注意事项？	
A	牙齿矫正期间需注意以下几点：1. **饮食方面**：避免硬、黏性或过甜的食物，如坚果、口香糖和糖果，以免损坏矫正器或导致龋齿；2. **口腔清洁**：每日刷牙至少三次，使用正畸专用牙刷、牙缝刷或冲牙器，保持矫正器和牙齿的清洁；3. **遵医嘱**：定期复诊，佩戴橡皮圈等辅助装置时遵循医生建议；4. **防护**：运动时佩戴牙齿护具，避免外伤；5. **健康习惯**：戒烟限酒，减少对牙齿和矫正效果的不良影响。坚持护理有助于获得理想矫正效果。
B	牙齿矫正期间需要注意以下事项：首先，保持口腔卫生，使用软毛牙刷刷牙，避免食物残留在牙齿和牙套间。其次，避免吃硬、粘性食物，如糖果、坚果和口香糖，以免损坏矫正器。定期按时复诊，确保矫正进度。佩戴矫正器期间，如出现不适或器械松动，及时联系牙医调整。此外，保持良好的饮食习惯，尽量避免过量摄入含糖饮料，减少龋齿风险。最后，遵循牙医建议，佩戴保持器以确保牙齿位置稳定。
C	戴牙套期间要特别注意口腔卫生,每餐后都要认真刷牙和冲洗。避免食用太硬、太粘或易碎的食物,如硬糖、口香糖、坚果等。定期按医生要求复诊调整牙套。如果感到疼痛不适,可以服用止痛药或使用正畸蜡来缓解。矫正初期说话可能不太顺畅,需要一段时间适应。运动时建议戴护齿套保护牙齿和牙套。保持良好作息和饮食习惯,注意补充钙质,有助于牙齿矫正。
D	正确刷牙，养成每次进食后彻底清洁口腔卫生的习惯。可以自行摘戴的矫治辅助装置，应按时戴用。避免咬过硬（防止矫治器脱落损坏，钢丝变形），过粘及甜腻的食物（腐蚀牙齿），少饮用碳酸饮料（酸性物质易使牙齿脱钙）。按时复诊，发现问题（如矫治器脱落）及时与医生交流。
E	1.口腔卫生。矫正牙齿需要佩戴矫正器，这会加大牙齿的清洁难度。因此，矫正期间要更加注意口腔卫生，早晚认真刷牙，饭后及时漱口，并定期洗牙。2.注意饮食。饮食上也要多加注意，尽量避免吃牛肉、金针菇等易塞牙的食物，也应避免吃较黏的食物，容易粘住托槽。3.按时复诊。矫正器调整的时间不宜过长也不宜过短，前期可能需要半个月左右复诊一次，后期的间隔会越来多长，医生会根据每个人的情况给出复诊的时间，一定要遵医嘱复诊。4.戴保持器。牙齿矫正不仅仅是牙齿在移动，还有牙槽骨的改建。牙齿排列整齐后牙槽骨的改建还没有完成，因此需要戴一段时间的保持器让牙齿稳定。
F	牙齿矫正期间，需注意口腔卫生，定期刷牙和使用牙线，避免硬、粘食物以防托槽脱落。定期复诊，遵医嘱调整矫正器。保持饮食均衡，避免影响牙齿移动的食物。
G	牙齿矫正期间需注意：保持口腔卫生，每次进食后刷牙，使用牙线和漱口水；调整饮食，避免过硬、过黏及辛辣刺激性食物；按时复诊，每 4-6 周调整一次矫正装置；避免咬硬物和剧烈运动，以防矫治器脱落；矫治器需正确佩戴和保存，如有损坏及时联系牙医。
H	牙齿矫正需要注意的问题：1. 矫正方案的选择要根据自身情况，在医生指导下选择合适的方案，才会有更好的效果。2. 口腔卫生护理要做好，保持口腔清洁，减少口腔炎症。3. 饮食要适当调整，保持清淡，少吃过硬以及粘的食物。建议选择正规医疗机构进行矫正，并定期复查。

28.牙齿矫正期间如何维护口腔健康？	
A	在牙齿矫正期间，口腔健康维护尤为重要。首先，坚持早晚刷牙和餐后漱口，建议使用含氟牙膏及正畸专用牙刷，确保清洁牙齿和矫正器之间的缝隙。其次，使用牙线或正畸专用间隙刷清除牙缝残留物，预防牙龈炎及龋齿。避免进食过硬、过粘的食物，以防矫正器损坏或松动。定期复诊让医生检查矫正进程和口腔状况，如有不适及时咨询医生。此外，可搭配使用抗菌漱口水，降低菌斑滋生风险。保持健康饮食，少吃甜食，增强牙齿抵抗力，确保矫正效果和口腔健康。
B	在牙齿矫正期间，保持口腔健康尤为重要。首先，要定期刷牙，使用软毛牙刷和正畸专用牙膏，确保牙齿、牙套和周围的牙龈干净。每天刷牙至少两次，每次刷牙时间不少于三分钟。其次，使用牙线或正畸牙线清洁牙套和牙齿之间的缝隙，避免食物残渣堆积。避免食用过硬、黏性强或含糖量高的食物，减少龋齿风险。此外，定期去口腔正畸科进行检查，确保矫正效果良好，及时调整矫正器。保持良好的口腔卫生有助于避免牙龈炎、蛀牙等问题。
C	牙齿矫正期间的口腔护理需要特别注意以下几个方面:首先,建议在每次进食后立即用温水漱口,清除食物残渣。使用软毛牙刷配合专业的正畸牙刷,采用正确的刷牙方法,特别注意清洁托槽和牙齿之间的区域其次,可以使用牙线和水牙线来清洁牙缝,但动作要轻柔,避免损坏矫正器。建议每天使用含氟漱口水消毒杀菌。最后,在饮食方面要避免过硬、粘性强的食物,减少糖分摄入。定期复诊时请医生检查口腔卫生状况,及时调整护理方案。保持良好的口腔卫生习惯,确保矫正效果。
D	1、把牙齿刷干净<br>每次餐后都要刷牙，包括吃零食之后！每次刷牙时间不少于三分钟，确保每一颗牙齿的唇面、舌面和咬合面都能得到清洁。<br>2、配合专业工具<br>冲牙器：冲牙器利用脉冲水流冲击的方式来清洁牙齿、牙缝的工具，能够强力去除牙刷无法到达的地方，有效清洁食物残渣和有害细菌。<br>电动牙刷：一般来说每次刷牙在 3 分钟，正畸矫正的患者建议 3-5 分钟。电动牙刷每分钟数千次的频率清洁牙齿，效率更高。同时配合巴氏刷牙法才能达到清洁口腔的最佳效果。<br>正畸牙线：专业的正畸牙线，一端特别加强了硬度，可以方便进入到牙齿里面清洁。对于“牙套同学”来说，牙线是一个不可缺少的清洁小工具哦。<br>牙间隙刷：将牙缝刷轻轻插入牙齿间隙，前后移动清洁每个牙面的牙垢，深入清洁普通牙刷和牙线无法进入的牙缝内部和弓丝、托槽间隙。<br>漱口水：无法及时刷牙时，代替刷牙使用，暂时抑制菌斑，清新口气。需要提醒的是：漱口不能代替刷牙。口腔清洁的主要途径还是刷牙。<br>牙套也要清洁：如果佩戴的是隐形牙套，需要在做好牙齿清洁的同时，维护好牙套的卫生。早上起床后和晚上睡觉前，可适当用牙刷配合少量牙膏刷干净，搭配清洁片或超声波清洗器，效果更佳，切记不要开水烫牙套，以免变形导致戴不上，影响矫正效果。
E	正畸专用牙刷每副包含两个不同设计的牙刷“V”型刷头和凸型刷头：<br>1.“V”型刷头<br>横刷法：将刷头横跨在矫治器上，左右摆动，清洁托槽、弓丝、结扎丝。<br>45 度角刷法：倾斜刷头，清洁托槽龈方和合方表面。<br>前牙清洁：刷头中部凹槽横跨前牙切端，清洁前牙切缘。<br>2.凸型刷头<br>横刷法：刷头倾斜与牙面呈 45 度角，清洁托槽龈方和合方牙齿表面。

	纵刷法：清洁舌侧牙面、咬合面及牙间隙，使得各个角落得到清洁。<br>牙间隙刷：将刷头深入牙缝，通过前后移动来清洁牙齿邻面和弓丝遮挡区域。<br>漱口水的辅助作用：漱口水含有抗菌成分，能有效辅助刷牙，增强菌斑控制，结合机械性清洁和漱口水的使用，被认为是控制菌斑较为有效的手段。<br>氟化物的预防作用：局部使用氟化物是预防牙釉质脱矿的有效手段。在粘接正畸托槽后，直接将氟保护漆涂于牙面，固化后提供保护，预防脱矿
F	牙齿矫正期间，应保持良好的口腔卫生习惯：1. 每天刷牙两次，使用软毛牙刷和正畸专用牙膏。2. 定期使用牙线和间隙刷清洁牙缝和矫正器。3. 避免硬、粘食物，以防损坏矫正器。4. 定期口腔检查和专业清洁，预防牙周病。5. 使用漱口水辅助清洁，减少细菌滋生。
G	牙齿矫正期间维护口腔健康的方法包括：正确刷牙，使用含氟牙膏，每天至少三次，每次不少于三分钟；使用牙线或冲牙器清洁牙缝；避免过硬、过粘食物；定期使用漱口水；不要用牙齿咬硬物；定期拜访牙医进行检查和清洁。
H	1、注意口腔卫生:矫正期间,由于托槽、弓丝等矫正装置的存在,口腔清洁难度加大。但必须认真刷牙,每次饭后都要刷牙,使用软毛牙刷和含氟牙膏。<br>2、调整饮食:避免食用过硬、过黏的食物,如坚果、硬糖、口香糖等,以免损坏矫正装置。可以多吃富含维生素和蛋白质的食物,如水果、蔬菜、瘦肉等,有助于牙齿和口腔组织的健康。

29.齿矫正期间，矫正器具的清洁方法是什么？	
A	在牙齿矫正期间，保持矫正器具清洁至关重要。每天饭后使用正畸专用牙刷和含氟牙膏，清洁矫正器及牙齿，确保去除食物残渣和牙菌斑。可使用牙间刷清洁矫正器与牙齿之间的缝隙，并配合漱口水减少细菌滋生。对于可摘式矫正器，每日用温水清洗，避免使用热水或刺激性清洁剂，以免损坏器具。同时，定期更换牙刷或牙间刷，确保清洁效果。如有疑问，及时咨询正畸医生以获得专业建议。
B	在牙齿矫正期间，保持矫正器具清洁非常重要。首先，每天早晚使用软毛牙刷和无酒精漱口水清洁牙齿和矫正器具，确保去除牙齿表面和器具上的食物残渣和细菌。其次，使用牙线或专用的矫正器具清洁工具清洁牙齿之间的缝隙。定期用温水清洗矫正器具，避免使用热水，因为高温可能损坏器具。每周可以用专用的清洁片或水溶性清洁剂进行深度清洁。最后，保持矫正器具的干燥，避免细菌滋生。
C	矫正器具的清洁方法主要包括以下几个方面：每次进食后及时用温水冲洗，去除食物残渣；每天早晚使用专用的矫正器清洁刷和含氟牙膏仔细刷洗，特别注意 brackets 周围和牙缝部位；定期使用泡沫清洁片浸泡，去除细菌和异味；避免使用过热的水清洗，以防变形；如果是可摘式矫正器，建议每周用温和消毒剂深度消毒一次。注意清洁时要轻柔，避免用力过猛损坏矫正器。
D	在佩戴矫正器时，不要吃东西或喝水。用隐形正畸治疗吃东西比其他类型的正畸更舒服，因为夹板被移除，使咀嚼更容易。重要的是 切勿在戴着牙套的情况下喝饮料。 水除外，因为它们可能会染色或变质。<br>每天清洗隐形矫治器。即使取出来吃喝，矫正器也会积聚污垢。因此，应该每天清洁它们，确保它们完全没有碎片。 对于清洁，我们建议使用肥皂和水或泡腾片进行日常使用。切勿使用牙膏。清洁它们的最佳时间是在饭前。<br>尊重对准器组的变化。隐形正畸专家强调，要尊重在适当时候更换一套隐形矫治器的需要。这通常是 根据专家的指示，每 7 至 15 天更换一次以便在规定的时间内达到牙片的排列效果。
E	刷牙。如果是摘戴方便的隐形牙套，刷牙时摘下牙套，使用普通牙刷即可;如果是传统托槽矫治器（即我们常说的钢丝牙套），可以使用正畸专用牙刷，它的设计主要是为了在刷牙时有效地避开托槽而设计的，进而有效地帮助你清洁矫治器上积存的食物残渣。
F	牙齿矫正期间，清洁矫正器具可采用以下方法：使用软毛牙刷轻柔刷牙套和牙齿，确保每个角落都清洁到位；使用含氟牙膏增强牙齿抗蛀能力；餐后使用牙线清理牙缝；定期使用漱口水消毒杀菌；避免使用硬质或尖锐工具清洁，以免损坏矫正器具。
G	牙齿矫正期间，矫正器具的清洁方法包括：使用软毛牙刷和牙膏刷洗矫正器表面及牙缝；每周至少用一次专用清洁片或清洁剂浸泡矫正器；饭后漱口；使用牙线清洁牙缝；还可借助冲牙器或超声波清洁器辅助清洁。同时，应定期更换牙刷，保持口腔卫生。
H	1、漱口:牙套通常是矫正牙齿整齐度的工具,能够对牙齿产生一定的拉伸,能够使牙齿进行移动,达到矫正的目的,在平时可能会出现食物残渣的残留,可以通过漱口的方式,能够减少局部的物质堆积。2、使用牙刷:在平时可以使用软毛的牙刷对牙套进行刷牙,也能够起到清洁牙套的效果。

30.牙齿矫正期间，如何选择牙膏和刷牙频率进行有效的口腔清洁？	
A	在牙齿矫正期间，选择含氟牙膏有助于预防龋齿，清洁力强且成分温和的牙膏能更好保护牙釉质和牙龈。避免使用颗粒过大的美白牙膏，以免损伤矫正器或牙釉质。建议每天刷牙至少三次，尤其是餐后和睡前，配合正畸专用牙刷、牙间刷或冲牙器清洁矫正器及牙缝。正确的刷牙方式包括将牙刷与牙龈呈 45 度角，以小幅度轻柔刷动，有效清除菌斑。
B	在牙齿矫正期间，选择牙膏时，应选用含氟成分的牙膏，能够有效预防蛀牙和牙齿敏感。此外，最好选择专为矫正人群设计的牙膏，这些产品能够帮助清洁牙套周围的死角。刷牙频率方面，建议每天刷牙至少三次，尤其是在餐后。使用软毛牙刷，并配合牙线或牙间刷清洁牙套周围的间隙，避免食物残留引发口腔问题。定期更换牙刷或刷头，以确保刷牙效果。最后，每半年定期检查牙齿健康，确保矫正过程顺利进行。
C	在牙齿矫正期间，建议选择含氟牙膏，可以有效预防蛀牙和牙齿脱矿。同时避免使用美白牙膏，因其可能含有过于刺激的磨料成分。特殊牙套专用牙膏也是不错的选择，能更好地清洁牙套周围区域。刷牙频率应该增加到每天 3-4 次，特别是每次进食后都要及时刷牙。建议采用改良版的巴斯刷牙法，将牙刷毛 45 度对准牙龈和牙齿连接处，轻柔地来回震动，确保清洁到牙套周围和牙缝间的食物残渣。每次刷牙时间不少于 3 分钟。
D	牙膏：含氟牙膏有助于预防蛀牙 ，但要选择正规厂家生产的品牌。<br>避免使用漱口水：漱口水中的化学物质可能刺激牙龈，建议少用或不用。<br>使用牙线和间隙刷：这些工具可以帮助清洁牙缝和矫正器下方难以触及的区域。<br>注意饮食：避免过硬、过粘的食物 ，如硬果壳、口香糖等，以免损坏矫正器。<br>细嚼慢咽：进食时要细嚼慢咽，避免因咀嚼不当导致矫正器移位。<br>两侧交替咀嚼：避免长期单边咀嚼，以防矫正器移位或变形。<br>使用冲牙器：冲牙器可以有效清除牙缝和矫正器周围的残留物 ，但要选择震感均匀、频率跨度大的产品。<br>使用电动牙刷：电动牙刷清洁效果更好 ，但要选择适合正畸人群的型号。<br>定期检查：每 3-6 个月进行一次全口检查 ，及时发现并处理口腔问题。<br>使用正畸蜡：涂抹在矫正器上可以减轻口腔溃疡的不适感 。<br>保持口腔湿润：多喝水，使用润唇膏，避免口腔干燥。<br>戒烟限酒：吸烟和饮酒会刺激牙龈，增加口腔疾病风险。
E	患者要认真刷牙，每次用餐以后都要刷牙，而且刷牙的时候使用 Bass 刷牙法，每次刷牙的时间不能少于三分钟，而且在每个区域水平颤动至少要达到十次以上。 最好使用正畸患者专用的牙刷，可以更好地清洁患者托槽周围附着的菌斑、软垢和食物残渣等。同时建议正畸患者使用含氟牙膏，可以防止牙齿脱矿。
F	牙齿矫正期间，建议选择含氟化物的牙膏以预防龋齿，并使用正畸专用牙刷或软毛小头牙刷。刷牙频率应增加，每天至少 3-4 次，每次饭后及睡前，每次刷牙不少于 3 分钟，确保每个牙面都清洁到位。
G	牙齿矫正期间，建议选择含氟牙膏以预防龋齿，刷牙频率应提高，**每天至少刷牙三次**，每次不少于两分钟，确保每个牙面都刷到。餐后也应漱口或刷牙，使用软毛牙刷，配合牙线清洁牙缝，保持口腔卫生，辅助矫正效果。
H	首先要选择一个合适的牙刷,牙刷可以选择小头软毛或者正畸专用牙刷,用含氟牙膏清洁牙齿的各个表面。每次早、中、晚三餐后以及进食一定的零食之后,最好都进行一次口腔清洁,这时候要把牙齿的每个面,包括唇面、舌面,还有咬合面都要清理干净。同时患者还可以配合使用一些牙间隙刷、牙线和冲牙器等辅助工具来更好的进行口腔清洁，刷牙时尤其要注意矫治器周围以及矫治器表面上，粘附的一些食物残渣和软垢要清理

	干净，如果清理不干净，很容易导致牙齿的脱矿，或者是釉面不干净的情况。
**31.牙齿矫正期间如何避免釉质脱矿？**	
A	在牙齿矫正期间，预防釉质脱矿的关键是保持良好的口腔卫生。每日至少刷牙两次，建议使用含氟牙膏，尤其是在餐后彻底清洁牙齿和矫正器周围的区域。辅以使用牙线或冲牙器清理牙缝及难以到达的部位。定期使用含氟漱口水加强釉质保护，减少细菌滋生。避免摄入高糖食品和饮料，以降低菌斑形成的风险。定期复诊时，请医生检查口腔卫生状况并给予专业建议。如果必要，可进行局部氟化物涂布，以进一步防止脱矿的发生。
B	在牙齿矫正期间，为了避免釉质脱矿，保持口腔卫生至关重要。每天刷牙至少两次，使用软毛牙刷和含氟牙膏，确保刷到牙齿每个部位，特别是矫正器周围的区域。同时，使用牙线或正畸专用的牙线棒清洁牙齿间隙。避免食用高糖、高酸食物和饮料，这些食物容易促进口腔酸性环境，导致釉质脱矿。定期进行牙科检查，确保及时发现和解决问题。此外，可考虑使用含氟漱口水，加强对牙釉质的保护。
C	为避免牙齿矫正期间的釉质脱矿，需要注意以下几个方面：首先，要养成正确的刷牙习惯，每次进食后及时清洁牙齿，使用含氟牙膏，采用正确的刷牙方法。其次，控制甜食和碳酸饮料的摄入，这些食物容易导致牙齿酸蚀。再次，可以使用医生推荐的含氟漱口水，帮助预防脱矿。此外，定期进行口腔检查和洁牙，及时发现并处理问题。最后，保持充足的钙质摄入，可以通过食用乳制品和深绿色蔬菜来补充。
D	1、注意饮食，多饮白开水。要注意饮食调养，保护牙齿，防止已经脱矿的牙齿加重。避免进食过冷过硬或者甜食、酸性食物，以免造成牙齿不适感。平常注意多饮白开水，起到清洁口腔的作用。<br>2、减少喝碳酸饮料。注意节制饮用碳酸饮料，比如：可乐、或者是那种运动型的饮料，里面含有多种的有机酸，而这些有机酸能分解钙质，进而侵蚀到牙齿，造成牙齿脱矿。<br>3、正确选择牙刷。需要选择正确的牙刷进行刷牙，虽然说硬毛牙刷清洁效果好，但硬毛牙刷对牙齿的磨损较软毛牙刷的大，如果经常使用的话，会损伤牙釉质，加重脱矿，而且也使得牙齿容易变黄。正确的做法应该是硬毛牙刷和染毛牙刷交替使用，每隔 3 到 4 天使用一次硬毛牙刷，对牙齿进行一次大清理。<br>4、补充钙磷。坚固牙齿，促进牙齿吸收更多的钙，使用安全有效的牙齿再矿化制剂。
E	想要预防脱矿，需要额外注意牙齿清洁，同时在戴牙套期间减少食用饼干类、膨化食品类、碳酸饮料等，还可以辅助使用含氟牙膏。 对于牙周有问题或是已有脱矿现象的，其实更建议佩戴方便清洁的隐形牙套，而在每次复诊的过程中，我们也会做好监控，以防脱矿情况出现。
F	牙齿矫正期间避免釉质脱矿，需注意以下几点：保持口腔卫生，饭后刷牙或漱口；饮食上避免过冷过硬或甜食、酸性食物，多饮白开水；减少碳酸饮料摄入，因其含有有机酸会分解钙质，导致脱矿；使用含氟牙膏，促进牙釉质再矿化。
G	牙齿矫正期间避免釉质脱矿的方法包括：定期刷牙，使用牙线和漱口水；选择含氟牙膏；定期进行专业牙周保健；补充适量钙磷；均衡饮食，多吃富含维生素 C 和 D 的食物；尽量避免喝酸性饮料。若发现牙齿颜色改变或敏感度增加，应及时就医。
H	1.注意保持口腔卫生。每天至少早晚两次刷牙,饭后半小时刷牙最佳,既不会将牙齿表面矿物质刷掉,又能及时清理口腔细菌,在细菌产生酸性物质腐蚀我们的牙齿之前就将其扼杀在摇篮中。其他时候进食完,不刷牙至少也要进行漱口。2.节制饮用碳酸饮料,比如可乐、运动型的饮料,里面含有多种有机酸,而这些有机酸能分解钙质,进而侵蚀到牙齿,造成牙齿脱矿。

32.牙齿矫正期间饮食的注意事项有哪些？	
A	在牙齿矫正期间，饮食需特别注意以保护矫治器和牙齿健康。应避免咀嚼硬物，如坚果、骨头、硬糖等，防止矫治器损坏。尽量少吃粘性食物，如软糖、口香糖，以免食物残留难以清洁。减少酸性和甜食摄入，防止龋齿和牙釉质受损。推荐选择柔软易咀嚼的食物，如蔬菜泥、煮软的米饭、熟透的水果等。饭后使用正畸专用牙刷或牙间刷清洁牙齿和矫治器，保持口腔卫生，避免食物残渣堆积引发牙龈炎或其他口腔问题。定期复诊，及时调整矫治器并咨询饮食相关建议。
B	牙齿矫正期间，饮食要特别注意。首先，应避免食用硬的、粘的或富含糖分的食物，如坚果、糖果和口香糖，以免损坏牙套或引起蛀牙。其次，避免咀嚼过硬或带有骨头的食物，如硬壳食物和骨头。食物最好切成小块，避免使用矫正牙齿的部位过多咀嚼。可以选择软食，如汤、蒸菜、泥状食品等。每次进食后，要认真刷牙，保持口腔清洁，防止食物残渣滞留在牙套中，影响矫正效果和口腔健康。
C	在牙齿矫正期间，饮食注意事项主要包括以下几点：1. 避免过硬、过韧的食物，如坚果、硬糖、口香糖等，以防损坏牙套或导致牙齿疼痛。2. 不要食用粘性食物，如太妃糖、软糖等，这些食物容易粘在牙套上，不仅难以清理，还可能导致牙套脱落。3. 水果和蔬菜最好切成小块食用，避免直接啃咬。4. 建议食用软质食物，如粥、面条、蒸煮充分的肉类等。5. 进食后要立即漱口，并认真做好口腔清洁，以预防蛀牙和牙周炎。6. 尽量避免含糖饮料和酸性食物，减少对牙齿的伤害。
D	1、避免高糖、高粘食物。牙齿矫正期间，糖果、饼干、碳酸饮料、糯米制品等，都是尽量少吃的。因为牙齿矫正期间防蛀是矫正过程的重中之重。矫治器的存在给我们的口腔清洁造成了比较大的麻烦，这些粘性的食物会粘在矫治器上，极难清理。再者，一些丝状且韧性高的食物也要少吃，比如金针菇、韭菜...这些容易塞牙的食物，清理起来也比较费事儿。<br>2、避免吃过硬的食物。如棒冰，排骨，鸡腿，牛肉干，带核带壳的零食，因为吃这类食物必须花费比较大的力气去咬或咀嚼，容易使矫正器咬松脱，也有可能会把矫治钢丝咬变形，影响到牙齿的移动，较为重要的是，牙齿矫治过程中，牙齿处在不断改建的过程中，此时的牙齿本身并不是很牢固，大力的咀嚼容易伤害牙齿。<br>3、避免辛辣刺激食物。辛辣刺激的食物容易引起口腔溃疡，引发牙龈炎等，这些都是不利于牙齿矫正的。<br>4、尽量不喝含糖量高的饮料。可乐、雪碧、果汁等含糖量比较高的食物，在一定的程度上都可能引发龋齿以及牙齿脱矿等危险，不利于矫正。<br>5、 要多吃的食物。多吃富含维生素 C 的食物，因为它有助于骨骼的愈合和牙周韧带的生长。至于种类嘛，橙子、柠檬、葡萄、芒果、菠菜、马铃薯等新鲜的蔬果都可以选择，选什么就看你自己啦。保护牙齿的同时也不要亏待我们的胃。
E	1. 矫正期间坚决不能吃坚硬食物。刚做完矫正的人群如果吃到上述“危险”的食物，会有咬掉托槽的风险。而在矫正中后期托槽掉落还会影响矫正进度。2. 少吃黏食、甜食。比如蛋糕、年糕、口香糖之类。因为托槽表面非常容易粘附食物，这些黏食、甜食尤其不容易清除，久而久之积累在托槽周遭，容易造成牙面脱矿和蛀牙。3. 拒绝碳酸饮料。碳酸饮料会酸蚀牙齿，加快牙齿脱矿。4. 多吃蔬菜水果。矫正期间，因为托槽以及钢丝的存在，口腔黏膜会经常被刮蹭，引发口腔溃疡，患者通常由此感觉不适。5. 可食用流质或半流质食物。刚戴上牙套一到两周，牙齿开始受力，患者通常会觉得牙齿酸软松动无力，属于正常现象，无需惊慌。这时尽量吃煮软的面条、粥之类的流质半流质的食物，避免牙齿咀嚼硬物，加重疼痛不适。6. 矫正期间加强钙质摄入。此时牙槽骨在不断改建，适量钙的摄入还是很重要的。
F	牙齿矫正期间，应避免硬、粘、脆的食物，如坚果、糖果和硬糖，以免损坏矫正器。

	同时，减少高糖食物和饮料的摄入，以防蛀牙。建议多吃软食，如糊状食物、煮熟的蔬菜和水果，以及富含钙和维生素 D 的食物，以促进牙齿健康。
G	牙齿矫正期间饮食需注意：避免过黏如年糕、奶糖，过硬如坚果、螃蟹，过烫食物；选择松软易咀嚼的食物，如面包、牛奶；保持饮食清淡，多吃蔬菜水果，忌食辛辣；进食后需仔细刷牙，保持口腔卫生，定期进行口腔检查。
H	牙齿矫正期间饮食要注意：1. 避免食用辛辣食物：辛辣食物容易刺激口腔黏膜，导致口腔溃疡，造成牙龈疼痛。2. 避免食用黏性较大的食物：太黏的食物易损坏牙套。3. 避免食用质地较硬的食物：容易损伤牙齿，如肉干、红薯干、坚果等。4. 牙齿矫正期间要清淡饮食，多喝水，按时刷牙。牙齿矫正的原理是利用外力改变牙齿生长方向，需避免对牙齿造成额外负担。

33.如何正确佩戴保持器？	
A	保持器的正确佩戴方法如下：首先，确保保持器干净卫生，使用前可用温水轻轻冲洗。佩戴时，将保持器对准牙弓，轻轻按压至完全贴合牙齿，不要用牙齿咬合以避免损坏。每天佩戴时间应遵循医生建议，通常为 22 小时，仅在进食和刷牙时取下。取下后需存放于专用盒中，避免高温或弯折。定期清洗保持器，使用软毛牙刷和清水清洁，不使用牙膏以免刮伤表面。如有不适或损坏，应及时联系医生调整或更换。
B	佩戴保持器时，首先确保牙齿清洁。将保持器轻轻放入口腔，确保它与牙齿完全贴合，不应感到不适或疼痛。佩戴时要注意不咬紧保持器，而是轻松地将其安放在牙齿上。保持器应全天佩戴，特别是晚上睡觉时。如果佩戴期间出现异物感或不适，应及时调整或咨询牙医。此外，保持器应定期清洁，用温水和牙刷轻轻刷洗，避免使用热水或强力清洁剂，以免损坏保持器材质。保持器的佩戴时间应根据医生建议严格遵循。
C	正确佩戴保持器的步骤如下:首先用温水和牙膏把保持器清洗干净,轻轻擦干。然后对照牙齿形状,将保持器放入口腔,用手指轻轻按压直到完全就位。佩戴时间应遵医嘱,通常建议白天和睡觉时都要戴。进食前要取下保持器,放入专用盒中。饭后刷牙清洁后再戴上。每天都要清洗保持器,保持卫生。如果感觉不适或保持器损坏,及时就医调整。
D	如何正确取戴保持器？ 配戴上颌牙套时，双手持保持器，对准相应牙齿位置，大拇指均匀用力向上压。 摘取时，双手食指从后往前均匀用力向下，取出保持器。 配戴下颌牙套时，双手持保持器，对准相应牙齿位置，食指均匀用力向下压。
E	实际上现在分为几种，如果是固定式的，就是常说的舌侧保持器，实际上是不需管，只需观察保持器跟牙面贴合度好不好，控制好牙周问题，控制好口腔卫生问题，其他不需要佩戴。学习怎么佩戴主要是两种，透明的压膜式保持器、钢丝保持器。实际佩戴过程基本差不多，压膜式保持器，戴的过程中，可以先戴前，再戴后，然后先戴左、后戴右，因为现在保持器矫正完后，牙齿状态会有变化，戴的过程当中实际上就涉及到左边跟右边哪边戴会更顺利点，同时一般的牙齿，前牙佩戴起来是最困难的，后牙相对来说容易点，因为前牙有倾斜度。如果要佩戴，一般建议先把前牙这个地方压住，然后再压入后牙。但是要避免以下动作，第一个，佩戴的过程当中，出现摘很费劲，或者戴的过程中不是特别顺利，要注意看看是不是方向问题；第二个，是不是戴的位置不准确，如果出现这个问题，要及时纠正，如果没有出现这个问题，又使用暴力来做，那保持器会出现断裂问题。
F	正确佩戴保持器的步骤如下：首先清洁双手和保持器，然后将其放入口中，从后牙开始，用食指将保持器就位，先下后上。确保保持器完全贴合牙齿，避免咬合过紧。佩戴后，检查保持器是否平整，如有不适，及时联系牙医调整。每天清洁保持器，保持口腔卫生。
G	正确佩戴保持器需先清洁双手和保持器，再将保持器对准牙齿，一般先戴前牙再戴后牙，轻轻按压使其完全就位。佩戴过程中需保持口腔卫生，每天佩戴时间需遵医嘱，一般为每天 8-10 小时，可选择在晚上睡觉时佩戴。
H	1、佩戴前的准备：在佩戴保持器之前，确保双手清洁，然后将保持器对准上下牙齿的位置，从前牙开始轻轻按压，确保保持器与牙齿贴合。2、佩戴方法：透明压膜保持器的戴入方向应从前往后，取下时则从后向前，由里往外。佩戴时应避免使用暴力，以免损坏保持器。3、注意事项：刚戴上保持器时可能会出现干呕、大舌头、口水增多等情况，这是正常现象，几天后会缓解。如果保持器压到粘膜或牙齿导致疼痛，应及时复诊调整。4、佩戴时间：常规病例至少需佩戴两年，前 6-12 个月需 24 小时佩戴，之后可根据医生指导调整佩戴时间。特殊病例如成人或严重牙周病患者可能需要更长时间甚至终身佩戴。5、饮食注意事项：吃饭时需摘下保持器，饭后清洁牙齿后再戴上。佩戴保持器时可以喝温凉水，避免过烫的水或有色的饮料。6、清洁和保存：保持器需用冷水冲洗干净，避免使用热水。可以用柔软的小头牙刷和牙膏清洁，也可以使用保持器清洁片。保持器是塑料制品，容易折断，应妥善保管在保持器盒子里。

34.保持器的佩戴时间多久？	
A	保持器的佩戴时间因个体情况和矫正目标不同而有所差异。一般建议在正畸治疗结束后的头 1-2 年内全天佩戴保持器，以维持牙齿在新位置的稳定性。此后，可根据医生建议逐渐过渡到夜间佩戴。对于存在严重牙列不齐或咬合问题的患者，可能需要更长时间的佩戴，甚至终身夜间佩戴。此外，保持器的定期检查和清洁也十分重要，以确保其功能正常。具体佩戴时长应由专业正畸医生根据患者的口腔情况和骨骼变化特点制定个性化方案，并定期随访以评估效果和调整方案。
B	保持器的佩戴时间因个人情况而异，但通常建议在正畸治疗结束后的前六个月内，每天佩戴保持器至少 22 小时，除了吃饭和刷牙时可以取下。之后，佩戴时间可以逐渐减少，转为夜间佩戴，并维持至少两年。在这段时间内，保持器的佩戴有助于防止牙齿恢复到原来的位置，保持矫正效果。长期佩戴保持器是确保正畸效果稳定的关键，因此即便治疗结束后，也应根据医生的建议定期检查，确保牙齿保持在理想位置。
C	保持器的佩戴时间一般要遵医嘱,通常分为全天候佩戴期和夜间佩戴期两个阶段。第一阶段需要全天候佩戴 3-6 个月,除了吃饭和刷牙时可以取下。第二阶段转为夜间佩戴,持续时间约 6-12 个月。具体佩戴时间需要根据患者的牙齿情况、年龄、治疗效果等因素由医生评估决定。若未按要求佩戴,可能会导致牙齿复发,影响正畸治疗效果。建议患者严格遵医嘱执行。
D	一般情况矫正完成后的 2 年都要坚持佩戴保持器。
E	通常建议在矫正完成后至少全天佩戴保持器一年到两年，这是为了确保牙齿在新的位置上稳定下来。 在这段时间内，保持器几乎需要 24 小时佩戴，除了吃饭和刷牙。 经过初步的全天佩戴后，可以逐渐减少佩戴时间，比如转为夜间佩戴。 一般建议至少在接下来的一到两年内每晚佩戴保持器。 对于某些情况，如严重的错颌畸形、成年后矫正或有复发倾向的患者，医生可能会建议长期或终身佩戴保持器，尤其是在夜间。
F	保持器的佩戴时间一般建议为 2 年左右，其中第一年需要全天佩戴，除了吃饭和刷牙时取下；第二年可以逐渐过渡到仅夜间佩戴。之后，根据个人情况，可能需要逐渐减少佩戴时间，如隔天夜间佩戴等。具体佩戴时间需遵循医生建议，因为每个人的牙齿状况和稳定性不同。
G	保持器的佩戴时间因个体情况和正畸治疗的具体情况而异。一般来说，在取下牙套后的最初几个月，需要全天佩戴保持器，之后可能逐渐过渡到仅夜间佩戴。整体而言，可能需要佩戴保持器一年左右，甚至更长时间，以确保牙齿稳定在新的位置。建议遵循牙医的建议，以确保正畸效果的持久性。
H	保持器的佩戴时间因个体情况而异，通常在 1 年到终身不等。具体时间取决于牙齿的情况、日常口腔护理情况、矫正类型、年龄、牙齿稳定性、保持器类型以及患者依从性等因素。<br>轻度牙齿畸形：通常需要佩戴 1-2 年。这类患者的牙齿排列相对整齐，矫正后较为稳定，佩戴时间较短。<br>中度至重度牙齿畸形：可能需要 2-3 年，甚至更长时间。这类患者的牙齿位置显著异常，矫正后需要更长时间的固定以防止复发。<br>特殊患者群体：如青少年、有不良口腔习惯或牙周组织不健康的成年人，可能需要更长时间甚至终身佩戴。青少年处于生长发育高峰期，成年人有不良习惯或牙周组织不健康，牙齿位置相对不稳定，佩戴时间需相应延长。

35.佩戴保持器时有什么注意事项?	
A	佩戴保持器时应注意以下几点：1. **佩戴时间**：按医嘱佩戴，初期需全天佩戴，进食、刷牙时可取下，后期可逐渐改为夜间佩戴。2. **清洁维护**：每日用软毛刷和清水清洁，不使用牙膏或热水，以免损坏。3. **饮食注意**：避免戴着保持器进食，以防损坏或细菌滋生。4. **定期检查**：定期复诊，确保保持器适合口腔情况。5. **存放妥当**：不用时放入专用盒中，避免丢失或损坏。6. **不适症状**：若感到疼痛或松动，及时联系医生调整。
B	佩戴保持器时，应注意以下几点：首先，要按时佩戴，通常每天佩戴 12-16 小时，确保牙齿稳定。其次，保持器佩戴时不要咀嚼硬物或吃东西，以免损坏。保持器应定期清洁，使用温水和软刷轻轻刷洗，避免热水和强力清洁剂。佩戴时，如果出现不适或疼痛，及时联系牙医调整。此外，要避免将保持器放在潮湿或高温环境中，以免变形。最后，定期复诊，确保治疗效果。
C	佩戴保持器有以下重要注意事项:戴保持器前要刷牙,保证口腔卫生;每天至少佩戴 16-20 小时,按医生要求的时间严格执行;取下保持器时要放在专用盒中,避免损坏或丢失;定期用温水和专用清洁剂清洗保持器;若出现不适或保持器损坏要及时就医;吃东西前要取下保持器,吃完后清洁口腔再戴上;运动时最好取下保持器,以免发生意外;定期复查,遵医嘱调整。
D	如果您有固定保持器，请不要吃会损坏固定保持器的食物。不要把保持器包在餐巾纸中，因为它可能会被意外丢弃。不要把保持器长时间放在容器外。不要把保持器放在水里煮或用酒精清洗（包括含酒精漱口水）。不要用舌头用力舔或者用力咬保持器。不要把保持器放进洗碗机里。
E	1、正畸保持器戴用方法。透明压膜保持器戴入的方向是从前往后，取的方向则是从后向前，由里往外。请按医生指导的方法正确摘、戴保持器。<br>2、正畸保持器不适症状。刚戴上保持器的时候会出现干呕、大舌头、口水增多等情况，不必太过担心，这种情况过几天就会缓解。如发现保持器压到粘膜或牙齿等异常疼痛，请及时复诊，进行适当调整。<br>3、正畸保持器佩戴时间。常规病例保持器至少需佩戴两年时间，前 6-12 个月需 24 小时佩戴，进食、刷牙时暂时取下；之后根据医生安排调整佩戴时间，晚上佩戴或者隔一天带一次，不可以自行减少时间。特殊病例，如成人，严重牙周病等患者，矫正结束后需要保持更长时间，甚至需终身佩戴。<br>4、正畸保持器饮食注意事项。吃饭时要摘下保持器的，吃完饭后清洁牙齿后再戴上，佩戴保持器时可以喝温凉水，不可喝过烫的水或有色饮料和含糖的饮料，不然保持器有可能变形或变色，如果不及时清洁，也可能引起蛀牙。<br>5、正畸保持器清洁要求。保持器需要仔细清理，在取下时用冷水冲洗干净，热水容易烫坏保持器，可用柔软的小头牙刷沾取些许牙膏轻轻刷去保持器的唾液以及可能存在的异物，也可以用保持器清洁片清洁保持器。<br>6、正畸保持器保存方法。保持器是塑料制品，质地较脆，容易折断，请妥善保管好，随身最好携带保持器盒子，特别是外出吃饭取下保持器的时候，不要随意就把保持器乱包在纸巾内，最后可能会和垃圾一起误扔，取下就立刻放在保持器盒子里吧。如果保持器有变形或丢失，需及时前来医院重新取模重新做，费用另收。<br>保持器佩戴较长时间后可能会出现裂痕，或者咬合面咬穿的情况，都不影响佩戴，如果保持器断成两节，也请暂时保持佩戴，尽早来院重新取模重做。<br>7、正畸保持器复诊要求。佩戴保持器后也要定期复诊，不过不像矫正时每月一次，可能频率会降低比如三月一次，半年一次，一年一次，每次复诊都要携带保持器给医生检查，医生会检查保持器是否变形，如有保持器破损变形等突发情况，请及时联系主治医生再做一副。如果没按要求佩戴保持器，没有定期来院让医生复查，导致牙齿畸

	形复发，可能需要重新矫正，按照全额进行收费。
F	佩戴保持器时，注意以下几点：确保保持器清洁，定期用专用清洁液或温水清洗；避免用热水，以免变形。佩戴时避免吃硬、粘食物，以免损坏保持器。定期复查，按医嘱调整。如有不适或损坏，及时就医。保持口腔卫生，预防牙周病。
G	佩戴保持器时需注意：初次佩戴可能影响发音，适应后好转；每天至少佩戴 20 小时以上，吃东西时取下；用牙刷和牙膏清洁保持器，避免使用开水或酒精浸泡；妥善保存在硬质盒中以防丢失；按照医嘱定期复诊，根据情况调整佩戴时间。
H	佩戴保持器期间，需要注意以下几点：1、饮食调整：以柔软、易咀嚼的食物为主，避免辛辣、刺激的食物。2、口腔卫生：保持器需要每天清洁，佩戴时确保完全就位，避免过紧导致压迫牙龈和口腔黏膜。3、遵医嘱：严格按照医生的要求佩戴保持器，注意保持器的清洁卫生，及时更换损坏或丢失的保持器。

36.牙齿矫正期间怀孕，有什么影响？	
A	在牙齿矫正期间怀孕通常是安全的，但需注意以下几点：首先，怀孕期间激素变化可能导致牙龈敏感或炎症，应加强口腔卫生。其次，避免非必要的牙科影像检查，尤其在孕早期，以减少胎儿暴露于辐射的风险。第三，孕期通常避免使用某些药物或材料，如含氟胶等。此外，孕期体位调整也很重要，长时间仰卧可能导致不适或低血压综合征。建议在怀孕前咨询牙科和产科医生，根据具体情况调整矫正计划。总之，保持定期复诊、良好沟通和口腔护理，可最大程度降低影响。
B	牙齿矫正期间怀孕通常不会对孕妇和胎儿造成直接影响，但有几点需要注意。首先，矫正过程中可能需要定期调整牙套，而怀孕期间荷尔蒙变化可能导致牙龈更易肿胀或出血，因此需要特别注意口腔卫生。其次，某些矫正治疗可能需要放射性检查（如 X 光），但孕期应避免进行此类检查。最后，孕妇应与牙科医生沟通，确保治疗方案适合其特殊情况。在怀孕初期或高风险期，最好推迟矫正治疗，等到产后再继续。
C	牙齿矫正期间怀孕需要特别注意以下几个方面：首先，怀孕期间激素变化可能会影响牙齿和牙周组织，使得牙齿移动速度略有变化，但通常不会对矫正效果造成显著影响。建议及时告知正畸医生怀孕情况，以便调整治疗方案。其次，在怀孕期间需要拍摄 X 光片时要格外谨慎。一般建议推迟到分娩后再进行，以避免辐射对胎儿的潜在影响。第三，保持良好的口腔卫生习惯更为重要，因为怀孕期间更容易出现牙龈炎症。建议每天认真刷牙和使用牙线，定期进行口腔检查。如果出现不适症状，应及时就医咨询。总体而言，怀孕和牙齿矫正可以同时进行，但需要更细致的关注和护理。
D	口腔内有许多敏感神经，牙齿矫正、戴矫正器的时候难免会碰触到神经，若孕妈咪因为神经受到刺激而引起宫缩、心跳加快，则有可能影响到胎儿的稳定发育。 另外，第一次装牙齿矫正器，很多人会因为牙齿感到不适、敏感而降低食欲，或无法好好进食，若孕妈咪食欲受到影响，则会无法满足宝宝对于营养的需求唷！
E	现有证据都表明， 牙齿矫正本身对怀孕没有任何影响，所以已经戴上牙套的准妈妈们无需过度担心。 但有以下几点需要在孕期内注意： 1. 一旦发现怀孕要及时告知你的正畸医生，对于某些矫正过程中的操作（如拍摄 X 线片、打骨钉等）医生会根据矫正方案进行适当时间上的调整。 2. 孕期由于体内激素水平发生变化，更容易发生牙龈肿痛，且由于进食习惯和次数的改变，牙齿表面更容易堆积食物残渣和脱矿。
F	牙齿矫正期间怀孕，对胎儿发育没有影响，但医生通常不推荐，因为孕期激素变化可能影响牙齿移动，从而影响矫正效果。若意外怀孕，应及时告知正畸医生，根据牙齿情况判断是否继续矫正。孕期矫牙需重视口腔卫生维护，避免牙龈炎症。
G	牙齿矫正期间怀孕，一般对胎儿没有直接影响，但可能增加孕妇的不适感，如疼痛和焦虑，进而影响孕期健康。同时，矫正器可能增加口腔清洁难度，易引发口腔疾病。因此，建议与正畸医生充分沟通，评估风险，必要时调整矫正计划。
H	怀孕对牙齿矫正影响不大，需注意口腔卫生：1. 牙齿矫正对怀孕不会有不利影响：怀孕期间若发现怀孕，一般也不会对胎儿产生不利影响。2. 保持口腔卫生清洁：需注意个人口腔卫生，刷牙要彻底，避免细菌滋生影响口腔健康。

37.牙齿矫正期间会口齿不清吗？	
A	牙齿矫正期间可能会出现口齿不清的现象，尤其是在初戴矫正器时。这主要是因为口腔内异物感增加，影响舌头的灵活运动和发音。但随着适应过程的进行，多数人能够逐渐恢复正常发音。此外，隐形矫正器相较于传统金属托槽，对发音的影响较小。为减轻不适感，可以通过朗读或刻意练习发音来帮助适应。若口齿不清问题持续严重，建议及时咨询正畸医生以调整矫正器设计，确保矫正效果与日常生活的平衡。
B	在牙齿矫正期间，部分人可能会出现口齿不清的情况。这通常是因为矫正器件（如牙套或隐形矫正器）在口腔中占据了一定空间，影响了舌头的活动，导致发音不够清晰。尤其是在刚戴上牙套的初期，口腔内的不适感可能使说话变得困难。不过，随着适应期的过去，舌头和口腔逐渐适应矫正器，大多数人的发音问题会逐步改善。如果发音问题持续较长时间，建议向牙医咨询，确保矫正器没有影响正常发音。
C	在牙齿矫正初期,确实可能会出现一定程度的口齿不清现象。这主要是因为口腔内装置占用了一定空间,舌头需要适应新的活动空间,发音时可能会受到影响。不过这种情况通常在1-2周内就会得到明显改善,随着舌头逐渐适应矫正器的存在,说话会恢复正常。建议在适应期间可以多做发音练习,比如朗读文章或对着镜子练习发音,这样能加快适应过程。如果佩戴隐形矫正牙套,发音影响会相对较小。
D	您的舌头可能需要几天时间来适应口中的 Invisalign 牙套，您可能会有轻微的口齿不清。
E	其实矫正器并不会引发口齿不清，该类口齿不清之感觉可能是主观的;即便病友有上述感觉该感觉几天内会自动甚至消失。
F	牙齿矫正期间，由于牙齿位置的改变和佩戴矫正器，一些人可能会暂时出现口齿不清的情况。但随着适应矫正器，这种情况通常会逐渐改善。
G	牙齿矫正期间可能会出现口齿不清的情况。这主要是因为矫正器作为异物存在于口腔内，患者尚未适应，以及牙齿移动可能导致咬合关系暂时改变。但通常这只是暂时现象，随着时间的推移和患者的适应，口齿不清的情况会逐渐改善。
H	不会。矫正牙齿本身不会导致口齿不清,但在矫正过程中可能出现咬合问题,从而引起发音不准。矫正牙齿可能会对某些发音产生积极的影响,但不能保证完全纠正所有发音问题,从而达到口齿清晰的目的。

38.牙齿矫正的费用是多少？	
A	牙齿矫正的费用因地区、医院级别、医生资质及矫正方案不同而有所差异。一般来说，传统金属托槽矫正费用约为 1 万-3 万元；陶瓷托槽稍贵，约为 2 万-4 万元；隐形矫正如隐适美，费用较高，通常在 3 万-8 万元之间。此外，还需考虑拍片、模型制作等额外费用。影响价格的因素包括牙齿复杂程度、矫正时长及患者年龄等。在选择时，应综合考虑医生资质与技术，避免因价格低廉选择资质较差的机构，以确保治疗效果与安全性。建议提前咨询专业口腔科医生，获取具体方案与报价。
B	牙齿矫正的费用因地区、医院、矫正方式和治疗周期的不同而有所差异。一般来说，传统金属牙套的费用在 10,000 至 30,000 元人民币之间，而陶瓷牙套的费用稍高，约为 20,000 至 40,000 元人民币。如果选择隐形矫正，如 Invisalign，费用通常在 30,000 至 60,000 元人民币之间。治疗的时间也会影响总费用，通常需要 1 至 2 年的时间。此外，部分医院和诊所可能提供分期付款或优惠活动，因此实际费用可根据个人需求和选择有所调整。
C	牙齿矫正的费用因多种因素而异。传统金属托槽正畸费用一般在 2-3 万元，隐形矫正费用则在 3-6 万元不等。具体价格取决于:矫正方案的选择(传统托槽/隐形矫正)、牙齿问题的严重程度、治疗时长(一般 1-3 年)、医院级别(三甲/私立)、医生资质、所在城市等。建议到正规医院进行专业检查和评估,制定个性化方案,了解详细费用。此外还要考虑复查、保持器等后期费用。
D	牙齿矫正的费用根据不同的矫正方式有所差异，传统牙齿矫正大约在 10 万到 12 万元之间，隐形矫正则在 18 万到 25 万元，正颌手术的价格则会超过 30 万元。实际费用会根据疗程的复杂度、矫正时间、牙材品质和医师技术等因素有所影响。
E	正畸费用看上去很贵，但实际上分散到 1~2 年左右，其实也还是大家能接受的范围。 不同的矫治方法价格有差别，跟治疗所在城市有关——经济越是发达的区域，矫正治疗也就更贵；正畸医生的专业程度越高，设计方案不同，价格也会不同。 比如三线城市医院传统金属矫治需要花费 1~2 万；到二线城市可能就需要 2～3 万；而在一线城市，即便用了最便宜的方案，也需要 3～4 万左右。 所以正畸费用在 1～3 万之间浮动是正常的。
F	牙齿矫正的费用因多种因素而异，包括矫正方式、材料选择、医院等级、医生经验以及患者的口腔状况等。根据 2024 年的数据，传统金属托槽矫正的价格大约在 5000 元至 20000 元不等，金属自锁托槽矫正约 12000 元起，陶瓷托槽矫正约 15800 元起，而隐形矫正的价格范围则在 18000 元至 50000 元不等。具体费用还需根据患者的实际情况和所选方案进行确定。
G	牙齿矫正的费用因多种因素而异，如矫正方法、材料、医院、地区等，一般在 5000 元至 50000 元不等。传统金属托槽矫正价格较低，隐形矫正价格较高。具体费用需根据个人牙齿情况和所选方案确定，建议咨询当地口腔医院获取准确报价。
H	牙齿矫正费用因情况而异：<br>1. 儿童期矫正费用：普通支架约 1 万元，自锁托槽需再加约 3000 元，隐形矫正和陶瓷矫正费用更高。2. 成年人矫正费用：一般 12000 元起，隐形矫正费用可能高达 30000-50000 元。种植体支抗和后期矫正会使费用增加。建议在最佳矫正年龄进行治疗，既可达到最佳治疗效果，也可节约费用。

39.牙齿矫正是费用越高越好吗？	
A	牙齿矫正的费用并非越高越好，效果主要取决于医生的专业水平、矫正方案的合理性以及患者自身的配合度。高费用通常与先进技术、优质材料或知名专家相关，但并不保证一定更适合所有人。选择矫正方案时，应综合考虑个人牙齿情况、经济能力和医疗机构的信誉。建议在多家机构咨询，了解医生资质和矫正方案细节，避免单纯以价格高低作为选择依据。合理选择，科学评估，才能获得满意的矫正效果。
B	牙齿矫正费用的高低并不一定代表效果的好坏。高费用通常与使用的技术、矫正器材的品牌、医生的经验等因素相关。虽然一些高端矫正方案可能提供更精准的治疗和更短的治疗周期，但并不代表适合每个人。治疗效果的关键在于医生的专业能力、治疗方案的选择以及患者的配合度。因此，选择合适的矫正方式才是最重要的。消费者在选择时应根据自身需求、预算和医生的建议综合考虑，而非单纯追求高价。
C	牙齿矫正的费用高低并不能简单等同于治疗效果。选择正畸医生和医院时,最重要的是要考虑以下几个方面:首先是医生的专业资质和临床经验;其次是诊所的医疗设备是否先进完善;再次是采用的矫正材料质量和品牌可靠性;最后是整个治疗方案的科学合理性。不同的病例复杂程度不同,所需费用自然也会有差异。建议选择正规医院,找经验丰富的专业医生进行诊断,根据自身情况制定合适的治疗方案,而不是单纯追求高价。
D	牙齿矫正费用并不是越高越好，矫正价格高的矫正方案也不一定意味著效果更好。 矫正牙齿费用取决于所选的矫正方式、个人牙齿状况以及治疗过程中的复杂性。 每个人的需求和情况不同，选择最适合自己的矫正方式才是最重要的，不建议单靠牙齿矫正费用来决定。
E	因为矫治器只是正畸医生手里的工具。无论哪种矫治器，治疗效果更多的取决于医生的能力和设计的矫正方案。牙齿矫正并不是一做就要做最贵的，能满足自己需求的才是最好的。所以该选择哪种矫正器，医生会根据你的个人综合情况，如预算、年龄、职业、错牙合畸形的严重程度等做出最合理的建议。
F	牙齿矫正并非费用越高越好。矫正效果取决于矫正方案的科学性、医生的专业水平和患者配合度。选择时应考虑医院设施、医生经验和患者自身情况，而非单纯价格。
G	牙齿矫正并非费用越高越好。牙齿矫正的效果主要取决于医生的专业技术、个性化矫正方案以及患者的配合程度。不同材料和方法的矫正费用有所不同，但高价并不等同于高质量。因此，在选择牙齿矫正时，应理性消费，选择适合自己的矫正方案。
H	牙齿矫正器并不是价格越贵就越好,具体的情况需要根据患者的自身情况进行判断。牙齿矫正器是一种常见的牙齿矫正方法,主要是通过矫正器对牙齿进行矫正,从而使牙齿恢复到正常的位置。如果患者本身牙齿比较健康,并且也没有其他牙齿方面的疾病,一般价格会比较便宜,可能在 5000 元左右。如果患者本身牙齿比较脆弱,并且也存在其他牙齿方面的疾病,一般价格会比较贵,可能在 10000 元左右。具体的价格通常与医院的等级、地区等因素有关,建议患咨询专业正畸医师的意见，根据自身的具体情况进行评估，避免盲目追求高价而忽视了适合自己的矫正方案。

40.牙齿矫正需要多长时间?	
A	牙齿矫正所需时间因个体情况而异，通常需要 1 至 3 年左右。具体时间取决于牙齿畸形的类型和严重程度、选择的矫正方式（如传统金属托槽、隐形矫正器等）以及患者的配合程度。轻微的牙齿排列问题可能仅需几个月，而复杂的咬合问题或骨骼发育异常可能需要更长时间。此外，年龄和骨骼可塑性也会影响矫正进度。完成矫正后，大多数患者还需佩戴保持器一段时间以防止牙齿移位。详细治疗计划应由专业正畸医生评估后确定。
B	牙齿矫正的时间因个人情况而异，通常需要 1 到 3 年。具体时长取决于矫正的目标、牙齿的复杂程度、年龄、治疗方式等因素。一般来说，成年人矫正可能需要更长时间，因为牙齿和骨骼的适应能力较差。常见的矫正方式包括传统金属牙套、隐形矫正器等。治疗过程中，患者需要定期复诊，调整牙套。治疗结束后，还需佩戴保持器以确保矫正效果持续。虽然治疗时间较长，但耐心和配合会帮助达到理想的效果。
C	牙齿矫正的时间因人而异,通常需要 1-3 年不等。影响因素包括:牙齿错位程度、骨骼发育情况、年龄大小、治疗方案选择以及患者的配合度等。轻度错位可能半年到 1 年就能完成,而严重错颌、地包天等情况则需要 2-3 年。矫正过程中需要定期复诊调整,保持良好的口腔卫生习惯。值得注意的是,摘掉牙套后还需要戴保持器一段时间,以巩固矫正效果。建议在开始治疗前,与正规医院的正畸医生详细沟通,制定个性化方案。
D	一般的矫正治疗大约需要二年左右的时间。
E	牙齿矫正需要的时间取决于患儿的年龄、错(牙合)畸形类型、矫正手段等，具体如下：<br>1、对于比较拥挤的牙齿，通过拔牙矫正排齐牙列，关闭拔牙的缝隙，矫正时间一般需要 2 年左右；2、如果患者年龄比较小，错(牙合)畸形相对不严重，乳牙反(牙合)以牙性为主，矫正时间可能需要 3-6 个月；<br>3、如果年龄比较大，牙周出现问题，矫正时间相对较长，可能需要 2-3 年；<br>4、隐形矫治，传统的矫正是用钢丝来矫正，力度相对较大，而隐形矫正表达和控制牙的能力相对较要弱，矫正时间相对较长，大约需要 3-4 年，比传统矫正增加 50%-100%的时间。<br>因此，牙齿矫正时间要因人而异、因病而异、因矫治器而异。
F	牙齿矫正的时间因个体差异而异，通常需要 1.5 至 2 年左右。青少年由于骨骼和牙齿仍在发育，矫正时间相对较短，一般为 1-2 年。成年人由于骨骼发育已完成，矫正时间可能会延长至 2-3 年。具体时间还受牙齿状况、矫治器种类、治疗预期、配合程度等因素影响。
G	牙齿矫正的时间因年龄、牙齿畸形程度和矫正方式而异。青少年矫正时间相对较短，一般需要 1-2 年；成年人矫正时间较长，可能需要 2-3 年。轻微畸形矫正需 1 年左右，严重畸形则需 2 年以上。矫正方式如隐形牙套、金属牙套等所需时间也各不相同。
H	牙齿矫正时间因年龄不同而异：1. 替牙期儿童矫正时间：反颌、拥挤等需要 3-6 个月。2. 替牙期活动矫治器：只能矫正大概的咬合关系，无法排齐牙齿。3. 成人矫正时间：一般需要 1 年半到 2 年，加上 2 年左右的保持器。4. 隐形矫治时间：一般需要 2 年半左右时间，相对更长一些。

41.牙齿矫正期间，如果没有时间复诊，半途放弃有什么后果？	
A	牙齿矫正期间半途放弃或未按时复诊可能导致多种不良后果。首先，矫正器持续施力可能导致牙齿位置异常移动，出现咬合紊乱或牙齿排列不齐的情况，甚至比矫正前更糟糕。其次，长时间不复诊可能引发口腔卫生问题，如龋齿、牙龈炎或牙周病，因为矫正器易藏污纳垢。最后，未完成的矫正可能导致治疗失败，浪费已投入的时间和金钱。建议与医生沟通调整计划，尽量按时复诊，以确保治疗效果。
B	牙齿矫正期间如果没有时间复诊或半途放弃，可能会导致多个后果。首先，矫正进程会中断，无法按计划达到理想的牙齿排列，甚至可能导致牙齿移位回原位。其次，牙齿矫正设备（如托槽、矫正器）可能因不及时调整而引发疼痛、口腔溃疡等问题，影响口腔健康。最后，未按时复诊可能导致矫治效果不稳定，增加矫正周期和治疗成本。为了确保效果，建议按时复诊并遵循医生的指导进行治疗。
C	半途放弃牙齿矫正可能会带来严重后果。首先，已经移动的牙齿会快速回到原位，前期投入的时间和金钱将付诸东流。其次，牙齿不规则移动可能导致咬合关系变得更糟，引起疼痛、咀嚼困难等问题。更严重的是，可能造成牙根吸收、牙周组织损伤，影响口腔健康。此外，重新开始矫正时，治疗难度会增加，费用也会相应提高。建议即使再忙，也要按时复诊，确保治疗效果。
D	牙齿矫正中途放弃的危害有可能会影响到矫正的效果，还有可能会导致牙齿出现松动的现象。
E	1、整体咬合错乱 矫正的过程是调整牙齿咬合系统的过程，中途放弃会导致上下牙合的错乱； 2、易复发 看似已经矫正整齐的牙齿实际牙齿的修复并未完善，若半途放弃极易导致牙齿复发成矫正前的样子； 3、牙齿歪斜 牙齿拥挤矫正需要拔牙，若间隙未关闭就放弃治疗，会导致其他牙齿向牙齿空隙非正常移动，导致牙齿歪斜影响咬合甚至容貌。
F	牙齿矫正期间如果半途放弃，可能会导致整体咬合错乱、易复发、牙齿歪斜和牙周病等问题。
G	牙齿矫正期间如没有时间复诊或半途放弃，可能导致矫治效果不稳定，牙齿松动甚至脱落，口腔感染，以及影响牙齿美观度。此外，还可能引发不可逆的牙齿问题，影响咀嚼功能。因此，建议坚持矫正，按时复诊。
H	牙齿矫正中途放弃可能会导致牙齿出现松动的情况, 而且可能会使牙齿倾斜的程度更加严重。在矫正期间最好不要放弃, 否则可能会对牙齿造成一定的伤害。牙齿矫正通常不可以中途放弃, 可能会影响矫正的效果。牙齿矫正通常需要坚持, 才能达到正畸的效果, 如果在中途停止之后, 牙齿还可能会恢复到原来的位置。

42.牙齿矫正需要拔牙吗？	
A	牙齿矫正是否需要拔牙取决于个体的口腔状况和治疗目标。通常，以下情况可能需要拔牙：牙齿拥挤严重，无法通过其他方式腾出空间；牙齿前突明显，影响美观或咬合功能；存在阻生牙或多生牙影响排列。但并非所有患者都需拔牙，医生会根据具体情况设计个性化方案，如使用扩弓或片切技术避免拔牙。在选择是否拔牙时，需综合考虑美观、功能和长期稳定性，与医生充分沟通后决定。
B	牙齿矫正是否需要拔牙取决于具体的口腔情况。通常，拔牙是为了给牙齿排列腾出空间，尤其是在牙齿拥挤、排列不整齐时。常见的拔牙原因包括前牙过于突出、牙齿过度拥挤或下颌前突等情况。然而，并非所有矫正治疗都需要拔牙，很多情况下可以通过其他方法如扩展牙弓或使用隐形矫正器来避免拔牙。因此，是否拔牙需要根据口腔医生的评估结果，综合考虑牙齿的排列情况、面部结构等因素。总之，拔牙并非矫正治疗的必选项。
C	牙齿矫正是否需要拔牙取决于个人的具体情况。主要考虑因素包括:牙齿拥挤程度、面部轮廓、咬合关系等。轻度拥挤可能通过其他方法解决,无需拔牙;中重度拥挤往往需要拔牙来腾出空间,通常会拔除第一前磨牙。此外,严重龅牙或反颌也可能需要拔牙来改善面型。建议先到正规医院进行专业检查,医生会根据口腔情况、X 光片等全面评估后,制定最适合的个性化方案。不是所有病例都需要拔牙,关键是遵医嘱。
D	正畸拔牙是为了改善牙齿排列、咬合关系和面部美观的一种治疗方法。 拔牙与否并不是医生的单方面决定，取决于患者的具体情况，经过医生的全面评估、反复斟酌，与患者充分地沟通，制定出最适合的正畸方案。 虽然拔牙会带来一些不适和疼痛，但是经过正确的治疗和护理后，取而代之的是更健康、更美丽的笑容。
E	1.牙床和牙齿配套，牙床有足够的空间容纳牙齿。这种情况，即使牙齿局部有不整齐，也是可以排整齐的，因此，不需要拔牙。2.牙床长度 ＞ 牙齿宽度（专业术语为牙列长度与颌骨长度），牙床有足够的空间容纳牙齿。<br>3.牙床长度 ＜ 牙齿宽度，牙床没有足够的空间容纳牙齿。如果强行把牙齿排齐，牙齿就会超出牙床的空间，这是行不通的，这时，我们就需要拔牙。
F	牙齿矫正是否需要拔牙取决于个人牙齿状况和矫正目标。并非所有矫正都需要拔牙，但在一些情况下，如牙齿过度拥挤或面部前突，拔牙可以帮助创造空间，改善咬合关系和面部轮廓。因此，是否拔牙应由正畸医生根据患者的具体情况分析后决定。
G	牙齿矫正是否需要拔牙取决于个体情况。如果牙齿稀疏状态正常或存在门牙外突、牙缝大等问题，通常不需要拔牙。若牙齿过度拥挤，可能需要拔牙以腾出空间。是否需要拔牙应由专业正畸医生评估后决定。
H	牙齿矫正是否需要拔牙取决于具体情况：1. 对于轻微排列不整齐的牙齿，咬合关系协调且牙齿之间有足够缝隙，一般不需要拔牙。2. 如果牙齿拥挤、缝隙不足，或咬合关系不协调、上牙突出，一般需要拔除一定数量的牙齿，以保证矫正过程中有足够缝隙，且矫正后的咬合关系良好。

43.进行牙齿矫正时，医生建议拔牙，如果不拔会有什么影响？	
A	在牙齿矫正中，拔牙通常用于解决牙齿排列不齐、拥挤或咬合问题。如果不拔牙，可能会导致矫正效果受限，牙齿难以排齐或咬合关系无法改善。此外，不拔牙可能导致牙齿过于前突，影响美观和功能。拥挤的牙齿也增加了清洁难度，可能引发龋齿或牙周病。但需根据具体情况判断是否拔牙，医生会综合考虑骨骼结构、牙齿排列和患者需求，制定最佳矫正方案。
B	在牙齿矫正过程中，如果医生建议拔牙，通常是为了给牙齿提供足够的空间来移动。若不拔牙，可能会导致牙齿拥挤、排列不整齐，甚至影响咬合功能。牙齿过于拥挤会使矫正效果不理想，治疗周期延长，甚至可能无法达到理想的美观和功能状态。此外，牙齿拥挤还可能导致口腔清洁困难，增加牙齿龋齿和牙龈疾病的风险。拔牙能够优化矫正空间，提高治疗效果，确保最终结果更加健康、持久。
C	不进行拔牙矫正可能会带来以下影响:首先，由于空间不足，牙齿拥挤的情况无法得到彻底改善，即使进行了矫正，牙齿也可能会出现重叠或排列不整齐的情况。其次，过度拥挤的牙齿会增加清洁难度，容易导致食物残渣堆积，引发蛀牙和牙周疾病。此外，不拔牙矫正可能会造成面部轮廓的改变，如面部突出或嘴唇外凸。更重要的是，不合理的牙齿排列会影响咬合功能，长期可能引起颞下颌关节疾病，出现咀嚼困难、关节疼痛等症状。因此建议遵医嘱进行合理的拔牙治疗。
D	正畸拔牙不會對身體健康造成影響，而醫生也會根據你的身體情況判斷你是否可以拔牙，拔牙以後直至矯正完成都不會對身體健康有任何影響。 甚至有一些牙齒是非拔不可的，比如已經壞死到神經的牙、成年後萌發的阻生智齒等等，不拔掉的話產生的疼痛、發炎等情況會非常影響患者的日常生活和工作。
E	当牙齿严重拥挤、前突或者咬合关系异常时，不拔牙很难达到理想的矫正效果。 例如牙齿过度拥挤会导致牙齿排列不齐，容易滋生细菌，引发龋齿和牙周疾病。 通过拔牙可以为其他牙齿的移动提供足够的空间，使牙齿能够排列整齐，改善咬合关系，提高口腔健康水平。 同时，对于一些因牙齿前突而影响面部美观的人来说，拔牙矫正可以使前突的牙齿内收，改善面部轮廓，提升颜值。
F	不拔牙进行牙齿矫正可能会导致拥挤未得到有效解决，影响矫正效果和面部美观。同时，可能增加矫正时间，提高复发风险。
G	进行牙齿矫正时，如果医生建议拔牙而不拔，可能会导致牙齿排列不齐、牙缝变大、咬合异常、牙齿松动、牙根吸收及牙龈炎症等问题。具体影响因个体情况而异，建议在专业医生指导下，结合个人实际情况进行决策。
H	矫正牙齿需视情况决定是否需要拔牙：1. 牙列拥挤、面部前凸、咬合关系不佳等病情严重时，需通过拔牙减少牙齿数目，以获得矫正牙齿所需的空间，有利于矫正效果稳定，畸形不易复发。2. 牙齿情况较好者，一般在矫正牙齿时无需拔牙。3. 拔牙对身体及咀嚼一般无明显影响，可选择拔除坏牙、松动牙、蛀牙或智齿等，尽量保留其他牙齿。

44.牙齿矫正如果需要拔牙，可能会有什么风险？	
A	牙齿矫正过程中拔牙可能带来以下风险：1. **疼痛与肿胀**：拔牙后可能出现短暂的不适或肿胀，需要时间恢复；2. **感染**：伤口若未妥善护理，可能引发感染；3. **牙根吸收**：矫正过程中牙齿移动可能导致邻牙牙根吸收；4. **牙槽骨损伤**：拔牙可能对牙槽骨结构造成轻微损害；5. **邻牙损伤**：操作不当可能损伤邻近牙齿或神经；6. **正畸后复发**：若后续保持不到位，牙齿可能再次移位。与专业医生充分沟通，选择合适的治疗方案可有效降低风险。
B	牙齿矫正过程中如果需要拔牙，可能存在以下风险：首先，拔牙后可能导致咬合问题或咬合不适，影响口腔功能。其次，拔牙可能引发感染、肿胀或出血，尤其是术后护理不到位时。再者，拔牙后，牙齿周围的空间可能需要时间来恢复，治疗周期可能延长。此外，某些情况下拔牙可能对面部外观产生影响，导致面部轮廓改变。因此，在进行拔牙前，应咨询专业牙医，全面评估风险与收益，确保矫正方案的可行性与安全性。
C	牙齿矫正需要拔牙可能存在以下风险：1. 手术相关风险：局部麻醉可能引起过敏反应；拔牙过程中可能造成牙槽骨损伤或周围软组织损伤；术后可能出现出血、疼痛和肿胀。2. 并发症风险：拔牙部位可能发生感染、干槽症（拔牙创面愈合不良）；邻近牙齿可能受损；极少数情况下可能影响下颌神经。3. 美学风险：拔牙后可能出现面型轻微改变，如脸颊凹陷；但通过正确的矫正方案，这种情况通常可以避免。4. 功能风险：可能暂时影响发音和咀嚼功能，但一般会在适应期后恢复。建议选择经验丰富的医生，严格遵医嘱进行术后护理。
D	正畸案例中，一般可能会牺牲前磨牙(双尖牙)和智齿，前磨牙在所有牙齿中贡献较小，拔了不会影响前牙的美观、切咬功能，也不会影响后磨牙的咀嚼功能。
E	牙齿矫正拔牙无危害性，但操作不当会诱发牙槽骨炎、牙神经损伤及其他并发症。 拔牙后未及时消炎牙槽骨，可能出现炎症反应，拔牙出现剧烈性疼痛。 拔牙后牙神经损伤包括内侧牙神经损伤和外侧牙神经损伤。 拔牙引起牙神经损伤较少见，是暂时性症状可恢复，但部分患者拔牙后牙神经损伤严重，可能永久存在，需及时修复牙神经。 拔牙后长期出血、肿胀，是牙齿矫正拔牙常见并发症，通过悉心护理，在 3~7 天左右可逐渐消失。
F	牙齿矫正拔牙可能存在的风险包括：咀嚼效率降低、影响食物消化吸收；牙齿排列不齐、影响美观和咬合功能；面部轮廓变化，如脸颊部位显得凹陷或扁平；牙齿敏感、不适或冷热刺激敏感；牙龈萎缩、牙齿松动；心理压力增加，影响心理健康和学习生活。此外，还可能引发口腔感染、牙槽骨吸收、颞下颌关节紊乱和牙齿移位等并发症。因此，选择经验丰富的正畸医生和遵循医嘱进行术后护理是降低风险的关键。
G	牙齿矫正如果需要拔牙，可能存在的风险包括局部损伤、炎症、牙龈损伤、神经损伤、邻牙松动、咬合问题以及影响面容等。因此，应选择正规医疗机构，由专业医生进行操作，并在术后注意口腔卫生和护理。
H	牙齿矫正拔牙有风险，需注意以下问题：1. 操作需要谨慎：牙齿矫正拔牙属于一种有创操作，需要到专业医疗机构，找有经验的医生来操作。2. 可能出现并发症：拔牙后可能会有炎症、牙龈损伤、神经损伤等并发症，需要注意术后护理。3. 需要检查健康状况：拔牙之前需要拍 X 光片检查，了解口腔整体健康状况，需要在无菌的情况下拔牙，将拔牙风险降到最低。

45.拔牙会影响其他牙齿吗？	
A	拔牙可能会影响其他牙齿的位置和功能，尤其是如果缺失的牙齿未及时修复。邻近牙齿可能会向缺牙空间倾斜或移动，导致咬合不正，增加龋齿和牙周病的风险。此外，对侧的牙齿可能会伸长，进一步破坏口腔的咬合平衡。长期缺牙还可能导致骨质流失，影响牙槽骨稳定性。因此，拔牙后建议及时进行修复，例如种植牙、桥修复或活动义齿，以避免对其他牙齿和口腔功能的不良影响。
B	拔牙可能会影响其他牙齿的健康和排列。首先，拔牙后，周围的牙齿可能会发生移动，尤其是如果是后牙的拔除，可能导致咬合关系发生改变，甚至影响整个牙列的稳定性。其次，长期空隙未填补可能导致相邻牙齿倾斜或牙齿过度磨损。拔牙后，如果不及时采取合适的治疗措施，比如佩戴牙桥、种植牙或使用活动假牙，可能会加剧咬合问题。为了避免这些不良影响，建议定期就诊，跟进医生的专业建议，确保口腔健康。
C	拔牙确实可能会对其他牙齿产生一些影响。首先，拔牙后形成的空隙会导致周围的牙齿逐渐向空隙方向倾斜或移动，影响牙齿排列和咬合关系。其次，缺失的牙齿无法承担咀嚼力，会增加其他牙齿的负担，可能加速牙齿磨损。此外，长期缺牙还可能引起对侧牙齿过度萌出，影响咬合平面。为了避免这些问题，建议在拔牙后及时进行修复，比如种植牙、烤瓷牙等，以维持口腔的整体健康。不过，如果是智齿，由于位置特殊，拔除后一般不会对其他牙齿造成明显影响。
D	不。牙齿拔除后，大概三个月左右，牙槽窝内就会填满新形成的牙槽骨，对邻牙的牙槽骨并无影响。<br>相反，当患牙有炎症，而通过治疗无法痊愈的，随着炎症范围的扩大，往往会影响到邻牙的牙槽骨，造成邻牙的炎症。<br>所以，当存在无法保留的患牙时，我们要尽早拔除。这不仅能防止患牙影响到健康的牙齿，在后期义齿修复时也能保留更多的牙槽骨，更好地进行种植修复。
E	拔牙是否会影响其他牙齿，要看是拔的哪个牙。如果是拔的牙弓最后端的智齿，对相邻的牙影响比较小，但会影响对颌的牙，如果拔除了下颌智齿，上颌智齿会慢慢伸长，有的伸长到可以咬到下颌牙龈。如果是拔除牙列内的某一个牙齿，相邻的牙齿会向缺隙处倾斜，对颌的牙齿会向缺隙处伸长。<br>这是人体自发的保护机制，会自动的关闭间隙。但是，如果邻近的牙齿倾斜，对颌牙伸长，会使得牙齿的咬合关系紊乱，使得牙弓的斯皮氏曲线和横颌曲线都发生改变，影响咬合关系。
F	拔牙可能会对邻近的牙齿产生影响。拔牙后，邻近牙齿可能会向拔牙区域移动，导致牙齿排列不齐。此外，拔牙后骨质可能减少，影响牙槽骨健康。但通过适当的护理和正畸治疗，可以减少这些影响。
G	拔牙一般不会直接影响其他牙齿，因为牙齿在解剖结构上是独立的。但如果拔的是普通恒牙且未进行适当修复，可能会导致相邻牙齿移位或咬合问题。拔智齿通常对其他牙齿无影响。拔牙后需注意口腔卫生和适当护理，以降低潜在风险。
H	拔掉正常恒牙对口腔及身体有多种影响：<br>1. 承担的咬合功能会缺失，导致消化负担加重，可能引发肠胃疾病。<br>2. 相邻牙齿逐渐向空位倾斜，导致咬合关系改变，可能引起颞颌关节紊乱。<br>3. 口腔卫生难以保持，可能引发其他牙周疾病。<br>解决方法：尽早进行口腔修复，如种植、活动义齿、烤瓷桥等，以解决以上问题。如果可能，建议尽量保留正常恒牙，如需拔除则尽快修复。
