# Supplementary material for: Comparing large language models and search engine responses to common orthodontic questions
Source: PLoS One. 2026 Jan 2;21(1):e0339908. doi: 10.1371/journal.pone.0339908 (PMC12758715; doi:10.1371/journal.pone.0339908)
Supplement: S8 Appendix — A indicates GPT-4o; B, GPT-4o mini; C, Claude 3.5 Sonnet; E, Kimi AI; F, ERNIE Bot; F, Google; G, Microsoft Bing; and H, Baidu. The midline indicates the median (50% percentile); the box, 25% and 75% percentile; the whiskers, 5% and 95% percentile; and the density distribution plot represents the probability density of the response score distribution. (PDF) [file pone.0339908.s008.pdf]

Medical accuracy (A), Completeness (B), Focus (C), Emotional Empathy (D), Cognitive Empathy (E), specialized vocabulary (F), and Logical Clearity (G) of LLMs and search engine responses to questions. A indicates GPT-4o; B, GPT-4o mini; C, Claude 3.5 Sonnet; E, Kimi AI; F, ERNIE Bot; F, Google; G, Microsoft Bing; and H, Baidu. The midline indicates the median (50% percentile); the box, 25% and 75% percentile; the whiskers, 5% and 95% percentile; and the density distribution plot represents the probability density of the response score distribution.

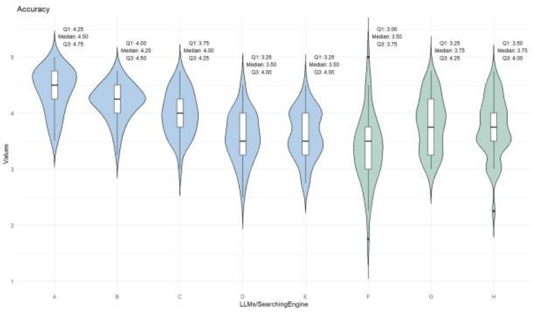

(A)

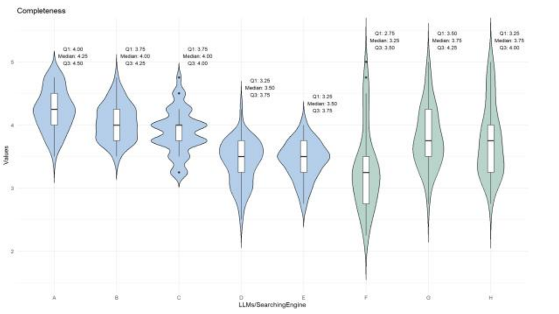

(B)

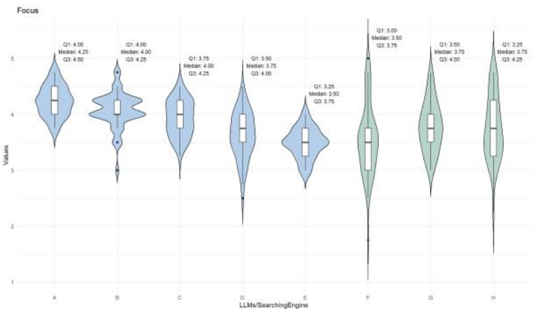

(C)

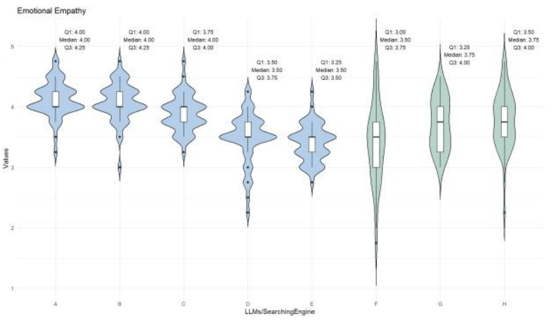

(D)

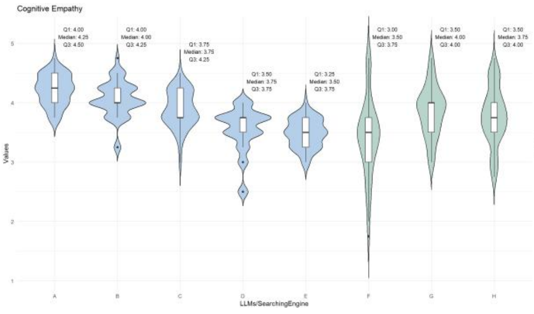

(E)

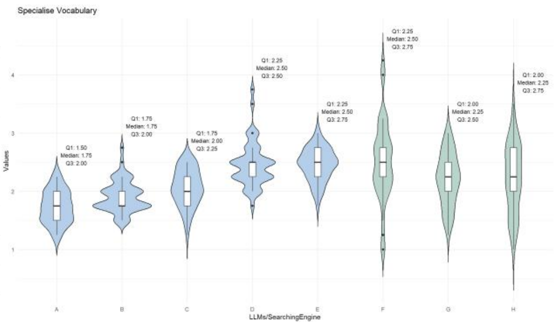

(F)

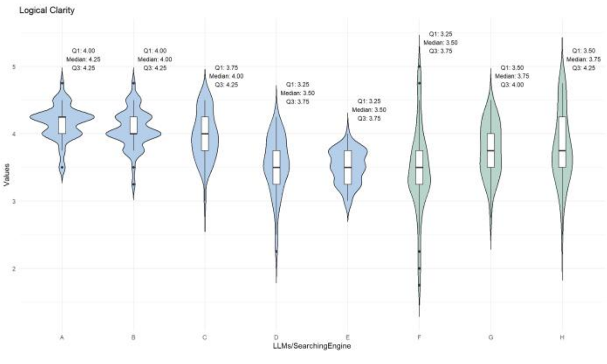
Logical Clarity
Q1: 4.00
Median: 4.25
Q3: 4.25
Q1: 4.00
Median: 4.00
Q3: 4.25
Q1: 3.75
Median: 4.00
Q3: 4.25
Q1: 3.25
Median: 3.50
Q3: 3.75
Q1: 3.25
Median: 3.50
Q3: 3.75
Q1: 3.25
Median: 3.50
Q3: 3.75
Q1: 3.50
Median: 3.75
Q3: 4.00
Q1: 3.50
Median: 3.75
Q3: 4.25
Values
5
4
3
2
A
B
C
D
E
F
G
H
LLMs/SearchingEngine

(G)
