# Supplementary material for: Comparing large language models and search engine responses to common orthodontic questions
Source: PLoS One. 2026 Jan 2;21(1):e0339908. doi: 10.1371/journal.pone.0339908 (PMC12758715; doi:10.1371/journal.pone.0339908)
Supplement: S9 Appendix — p-value: conducting statistical significance tests on the score differences between LLMs and search engines. (PDF) [file pone.0339908.s009.pdf]

Quality, Empathy, Readability, and Satisfaction Scores of LLMs and search engine responses to questions. p-value: conducting statistical significance tests on the score differences between LLMs and search engines.

	Response, median (IQR)		
	LLMs	Search Engines	p-value
Rater-evaluated quality score			
Medical accuracy	4.00(3.50,4.25)	3.50(3.25,4.00)	＜0.001
Completeness	3.75(3.50,4.25)	3.50(3.25,4.00)	＜0.001
Focus	4.00(3.50,4.25)	3.75(3.25,4.00)	＜0.001
Quality overall score	4.00(3.50,4.25)	3.50(3.25,4.00)	＜0.001
Rater-evaluated empathy score			
Emotional empathy	3.75(3.50,4.00)	3.50(3.25,4.00)	0.003
Cognitive empathy	3.75(3.50,4.25)	3.75(3.50,4.00)	0.007
Empathy overall score	3.75(3.50,4.00)	3.50(3.25,4.00)	＜0.001
Rater-evaluated readability score			
Specialize vocabulary	2.00(1.75,2.50)	2.25(2.00,2.75)	＜0.001
Logical clarity	4.00(3.50,4.25)	3.75(3.25,4.00)	＜0.001
Readability overall score	4.00(3.50,4.25)	3.75(3.25,4.00)	＜0.001
Rater-evaluated satisfaction score			
Satisfaction overall score	8.00(7.25,8.50)	7.25(6.75,8.13)	＜0.001
