# Supplementary material for: Comparing large language models and search engine responses to common orthodontic questions
Source: PLoS One. 2026 Jan 2;21(1):e0339908. doi: 10.1371/journal.pone.0339908 (PMC12758715; doi:10.1371/journal.pone.0339908)
Supplement: S16 Appendix — (PDF) [file pone.0339908.s016.pdf]

Summary of Supplementary Files (S1–S15)

Supplement	Content Description	Corresponding Section
S1 Appendix	Expert interview protocol	Methods: Question pool development
S2 Appendix	Basic information for clients and experts	Methods: Question pool development
S3 Appendix	45 common orthodontic questions	Methods: Question pool development
S4 Appendix	Sample response content table	Methods: Data extraction
S5 Appendix	Evaluation indicators and scoring criteria	Methods: Evaluation instrument
S6 Appendix	Self-designed multidimensional evaluation questionnaire	Methods: Evaluation instrument
S7 Appendix	Original Chinese-language model outputs	Data Collection
S8 Appendix	Indicator-level distributions	Results: Figure 2
S9 Appendix	Quality, empathy, readability, satisfaction scores	Results: Table 1
S10 Appendix	Bonferroni-adjusted comparisons	Results: Table 1
S11 Appendix	Effect sizes	Results: Table 1
S12 Appendix	Friedman test results	Results: Statistical robustness
S13 Appendix	Numerical data for heatmap	Results: Figure 3
S14 Appendix	Minimal anonymized dataset	Data Availability
S15 Appendix	R script for data analysis	Methods: Statistical analysis
